# Ramifications of generalized Feller theory

**DOI:** 10.1007/s00028-025-01148-9

**Published:** 2026-01-16

**Authors:** Christa Cuchiero, Tonio Möllmann, Josef Teichmann

**Affiliations:** 1 Department of Statistics and Operations Research, Data Science @ Uni Vienna, Vienna University, Kolingasse 14-16 1, 1090 Wien, Austria; 2 Department of Mathematics, ETH Zurich, Rämistrasse 101, 8092 Zurich, Switzerland; 3 Berlin, Germany

**Keywords:** Infinite-dimensional stochastic processes, Weighted spaces, Generalized Feller processes, Path properties, Transport equations on weighted spaces, 60G07, 60J25, 35Q49

## Abstract

Generalized Feller theory provides an important analog to Feller theory beyond locally compact metric state spaces. This is very useful for solutions of certain stochastic partial differential equations, Markovian lifts of fractional processes, or infinite-dimensional affine and polynomial processes which appear prominently in the theory of signature stochastic differential equations. We extend several folklore results related to generalized Feller processes, in particular on their construction and path properties, and provide the often quite sophisticated proofs in full detail. We also introduce the new concept of extended Feller processes and compare them with classical and generalized ones as well as with Doob’s h-transform. A key example relates generalized Feller semigroups of algebra homomorphisms via the method of characteristics to transport equations and continuous semiflows on weighted spaces, i.e., a remarkably generic way to treat differential equations on weighted spaces. We also provide a counterexample, which shows that no condition of the basic definition of generalized Feller semigroups can be dropped.

## Introduction

Feller processes on locally compact metric spaces are based on the well-developed analytic theory of strongly continuous semigroups, which simplifies considerably on the Banach space of continuous functions vanishing at infinity which is the function space of interest. There are two major shortcomings of this classical theory: First, the analytic frame appears too narrow, as the semigroups act on continuous functions vanishing at infinity, and second, the local compactness of the underlying state space is often too restrictive. This concerns important applications where the spatial dynamics is modeled via stochastic partial differential equations (see [19, 31]) or signed measure-valued processes, as, for instance, Markovian lifts of Volterra processes (see [1, 2, 17, 18]). Another recent application is in the area of infinite stochastic covariance modeling where processes take values in the cone of positive Hilbert–Schmidt operators (see [12, 24]). Signature stochastic differential equations (SDEs), where affine processes and machine learning methods meet, constitute another class of promising applications (see [4, 15] and also the related paper [25]). There, one has to deal with state spaces corresponding to subsets of the so-called group-like elements of the extended tensor algebra.

To accommodate such infinite-dimensional state spaces, the so-called *generalized Feller semigroups* have been introduced in [19, 31]. Based on these previous works, the goal of this article is to provide a comprehensive and self-contained presentation of the theory of *generalized Feller processes* and the new concept of *extended Feller processes.*

The theory of generalized Feller processes is built in analogy to classical Feller processes, i.e., via strongly continuous semigroups acting on certain Banach spaces of functions. With respect to the classical setting, the space of functions vanishing at infinity is replaced by the so-called ${\mathscr {B}}^{\rho }(E)$-functions. Indeed, here *E* is a weighted space equipped with some *admissible weight function* $\rho$. Moreover, ${\mathscr {B}}^{\rho }(E)$ contains functions whose growth is controlled by $\rho$ and which lie in the closure of continuous bounded functions with respect to a weighted supremum norm induced by $\rho$. Therefore—unlike Feller semigroups—generalized Feller semigroups act also on unbounded functions; however, other basic properties remain, in particular the definition is very similar: A generalized Feller semigroup is a family of positive linear bounded operators from ${\mathscr {B}}^{\rho }(E)$ to ${\mathscr {B}}^{\rho }(E)$ such that the semigroup properties are satisfied, the norm of the operators remains uniformly bounded for small times, and for any map *f* in ${\mathscr {B}}^{\rho }(E)$, the image under the semigroup converges pointwise to *f* as *t* approaches 0.

The important feature of generalized Feller theory is that the underlying state spaces go beyond local compactness and also beyond metrizability and separability. For recent related work which also allows for such state spaces, we refer to [26], which treats not necessarily linear strongly continuous semigroups on weighted spaces of continuous functions equipped with the so-called mixed topology. Note that already in simple situations an appropriate theory for non-locally compact state spaces is crucial: Indeed, take a Hilbert space *E* and consider *d* bounded linear operators $A_1,\ldots ,A_d \in L(E)$. Consider, in addition, a *d*-dimensional Brownian motion *B* and the stochastic differential equation

$$\begin{aligned} d \lambda _t = \sum _{i=1}^d A_i \lambda _t dB^i_t, \end{aligned}$$

starting at $\lambda _0 \in E$ that defines a family of stochastic processes $\lambda$. The state space of the Markov process $\lambda$ is *E*, which is not locally compact. Therefore, no results of Feller theory are applicable even though, due to Ito calculus, we know plenty about continuous trajectories, strong uniqueness, boundedness properties of the solutions, linearity, etc. Since bounded linear operators are also weakly continuous, we can replace the norm topology on *E* by the weak topology. With respect to the weak topology *E* is $\sigma$-compact (since balls are weakly compact) and actually a weighted space with weight, for example $\rho (\lambda )=1+ {\Vert \lambda \Vert }^2$. Therefore, $\lambda$ defines a generalized Feller process associated with the generalized Feller semigroup

$$\begin{aligned} P(t)f(\lambda _0): = \mathbb {E}[f(\lambda _t)] \end{aligned}$$

for $t \ge 0$ and $f \in {\mathscr {B}}^{\rho }(E)$. It would be fairly non-trivial to construct other strongly continuous semigroups associated with $\lambda$ due to the absence of local compactness or tractable invariant measures.

In the following, we explain the main contributions of this article. In Sect. 2, we recall important notions and results of generalized Feller theory, in particular the Riesz representation theorem proved in [19], allowing to characterize the dual space of ${\mathscr {B}}^{\rho }(E)$ as signed Radon measures whose total variation measure integrates the weight function $\rho$. We prove several lemmas in this section and provide in Proposition 2.6 a *weighted space version of the Stone–Weierstrass theorem* on ${\mathscr {B}}^{\rho }(E)$ in the spirit of Leopoldo Nachbin [29], see also [14, Theorem 3.6] for a version where the elements of the algebra can be unbounded.

This fundamental approximation result is needed in the *proof of the existence* of generalized Feller processes, given in Theorem 3.3. A similar statement was already formulated in [18, Theorem 2.11], but the full proof with all details is given in the current article. Theorem 3.3 yields stochastic processes whose conditional expectations are given by a strongly continuous semigroup (a generalized Feller one) even in cases when the space *E* is neither separable nor metric nor locally compact. This is a crucial difference to the theory of Feller processes and thus one of the main results of this article. With respect to the literature, let us remark that in [6] impressive general results beyond locally compact metric spaces are obtained given resolvents of transition semigroups, however, less emphasis is put on the functional analytic properties of the semigroups.

For the theory developed in the current paper, let us mention a subtle point, namely that generalized Feller semigroups act on $\mathscr {B}^{\rho }(E)$ functions which are, in general—as limits of continuous functions—only Baire-measurable, see Remark 3.2 for further details. The proof of Theorem 3.3 is based on a general version of the Kolmogorov extension theorem (see Theorem 15.26 in [3]). In order to apply it, we construct a projective family of probability measures. Here, the Riesz representation theorem extended to $\mathcal {B}^{\rho }(E\times E \times \cdots \times E)$ is essential to express continuous linear functionals on the sub-level sets of the admissible weight function by a (sub-)probability measure. As we let the sub-level sets of the admissible weight function converge to the whole space, such a sequence of (sub-)probability measures converges to a probability measure on $E\times E \times \cdots E$, yielding a projective family of probability measures with the desired properties. Then the generalized Feller processes is the canonical process on the product space when equipped with a product measure according to the general version of the Kolmogorov extension theorem.

Having constructed generalized Feller processes, we treat their path regularity. This is subject of Theorem 3.4, where it is shown that generalized Feller processes admit a càdlàg version if certain technical conditions are met (thereby closing a gap in a statement in [18]).

Starting from a generalized Feller semigroup, there are more ways than one to construct a related stochastic process. Indeed, in Definition 4.1 we introduce a new class of processes coined *extended Feller processes* that build on generalized Feller semigroups, but in contrast to generalized Feller processes, the weight function enters in the definition of the respective conditional expectations. For some Baire-measurable function *f*, they are given by the quotient between the generalized Feller semigroup applied to the function $f \cdot \rho$ and the weight function $\rho$ and can thus be compared to Doob’s h-transform. The existence of these extended Feller processes is then proved in Theorem 4.3 and Corollary 4.5, under the condition that the generalized Feller semigroup is quasi-contractive.

We then compare extended Feller processes with generalized Feller processes and notice in Proposition 4.8 that if both exist, their induced laws are equivalent measures. In Sect. 5, we establish a connection between generalized/extended Feller processes with classical ones. To be precise, we slightly generalize the classical definition of *classical Feller processes* to *Feller processes* when the conditions of metrizability and separability of the underlying state spaces are dropped. In this sense, we then get the following two relations: First, on locally compact spaces a Feller process is generalized Feller if the Feller semigroup applied to the admissible weight function remains bounded for small times (see Proposition 5.1); second, if the admissible weight function is continuous, then *E* is automatically locally compact and extended Feller processes can be reduced to Feller processes (see Theorem 5.6). In general, when $\rho$ is not continuous, extended Feller processes thus generalize the notion of Feller processes to spaces *E* that are only $\sigma$-compact, which explains their name.

As important examples of generalized Feller processes, we consider in Sect. 6.1 deterministic processes induced by semigroups of transport type. Indeed, we first show that generalized Feller semigroups of smooth operator algebra homomorphisms, precisely introduced in Definition 6.1, are given by

$$\begin{aligned} P(t)f=f \circ \psi _t, \quad t \ge 0, \, f \in \mathscr {B}^\rho (E), \end{aligned}$$

where $\psi$ is a continuous semiflow in time (and also in space when restricted to compact sets). The associated infinitesmial generator of such a generalized Feller semigroup is a smooth derivation, also called transport operator. This allows to treat transport equations on weighted spaces via strongly continuous semigroup theory. Moreover, if a transport operator generates a generalized Feller semigroup, the associated generalized Feller process corresponds to $(\psi _t)_{t \in \mathbb {R}_+}$, which is actually the solution of a differential equation in the weighted space. In Sect. 6.2, we consider then a stochastic setting with classical affine and polynomial processes on finite-dimensional state spaces and show in Proposition 6.10 and Corollary 6.12 that under certain minor conditions polynomial and affine processes are generalized Feller processes. This adds to the existing theory, since to date it is not known whether affine or polynomial processes on general state spaces are classically Feller or not. Finally, in Sect. 6.3 we also provide a counterexample, which shows that the additional condition, called **P4**, used to define generalized Feller semigroups and not needed for classical Feller processes *cannot* be dropped, as then strong continuity does not necessarily hold true anymore.

### Notation and basic definitions

Here, we introduce notation needed throughout the paper. For a topological space *E* we denote its Borel $\sigma$-algebra by $\mathcal {B}(E)$. We write $C_{b}(E)$, $C_{0}(E)$ and $C_{c}(E)$ for the spaces of continuous maps that are bounded, vanish at infinity and have compact support, respectively. Moreover, $\text {Id}$ denotes the identity operator on a given space.

A *transition kernel* $\kappa$ from a measurable space $\left( \Omega ,\mathcal {F}\right)$ to a measurable space $\left( E,\mathcal {\mathcal {E}}\right)$^1^ is a map $\kappa : \Omega \times \mathcal {E} \rightarrow [0,\infty ]$ such that for any fixed $B \in \mathcal {E}$, $\omega \rightarrow \kappa (\omega , B)$ is $\mathcal {F}$-measurable and every fixed $\omega \in \Omega$, $B \rightarrow \kappa (\omega , B)$ is a measure. It is called a *transition probability* if $\kappa (\omega , E)=1$ for all $\omega \in \Omega$.

If $\left( \Omega ,\mathcal {F}\right) =\left( E,\mathcal {\mathcal {E}}\right)$, we simply speak of a transition kernel/probability on $\left( E,\mathcal {\mathcal {E}}\right)$. A family $\left( p(t)\right) _{t\in \mathbb {R}_{+}}$ of transition probabilities on $\left( E,\mathcal {E}\right)$ is called a $semigroup$ $of$ $transition$ $probabilities$ on $\left( E,\mathcal {E}\right)$ if for all $x\in E$, for all $s,t\in \mathbb {R}_{+}$ and all $A\in \mathcal {E}$

$$\begin{aligned} p(s+t)(x,A)=\int _{E}p(s)(y,A)p(t)(x,dy) \end{aligned}$$

and

$$\begin{aligned} p(0)(x,\cdot )=\delta _{x} \end{aligned}$$

hold. Here, $\delta _{x}$ denotes the Dirac measure. Similar notions apply of course to transition kernels which do not satisfy $\kappa (x, E) =1$ for all $x \in E$. For transition kernels $\kappa$ with $\kappa (x,E)\le 1$ for all $x\in E$, one can add a so-called *cemetery state* $\triangle$ to *E*, and define on $E_{\triangle }:=E\cup \left\{ \triangle \right\}$ a transition probability

$$\begin{aligned} \kappa ':E_{\triangle }\times \sigma \left( \mathcal {\mathcal {\mathcal {E}}},\left\{ \triangle \right\} \right) \rightarrow \left[ 0,1\right] \end{aligned}$$

by

$$\begin{aligned} \left. \kappa '\right| _{E\times \mathcal {\mathcal {E}}}=\kappa \end{aligned}$$

and for any $x\in E$ and for any $A\in {\mathcal {E}}$

$$\begin{aligned} \kappa '\left( \triangle ,\{ \triangle \right\} )&=1\\ \kappa '\left( \triangle ,A\right)&=0 \\ \kappa '\left( x,\{ \triangle \} \right)&=1-\kappa (x,E) . \end{aligned}$$

For any function *f* on *E*, the convention is to extend it to $E_{\triangle }$ by setting $f(\triangle )=0$. Usually, the precise distinction between $\kappa '$ and $\kappa$ will not be made and $\kappa '$ will simply be called $\kappa$. Finally, for $t\in \mathbb {R}_{+}$ we define the $translation$ $operator$ via

$$\begin{aligned} \theta _{t}:\,E^{\mathbb {\mathbb {R}}_{+}}\rightarrow E^{\mathbb {\mathbb {R}}_{+}}, \quad \left( x(s)\right) _{s\in \mathbb {\mathbb {R}}_{+}}\rightarrow \left( x(s+t)\right) _{s\in \mathbb {\mathbb {R}}_{+}}. \end{aligned}$$

## Notions of generalized Feller theory

We here recall the essential notions of generalized Feller theory developed in particular in [19] and prove some basic lemmas as well as the weighted Stone–Weierstrass theorem which will be used later on.

Let *E* be a completely regular space (c.f. Definition 4 in Chapter IX of [9]). A map $\rho :\,E\rightarrow \left( 0,\infty \right)$ is called an $admissible$ $weight$ $function$ if the sets

$$\begin{aligned} K_{R}:=\left\{ x\in E:\,\rho (x)\le R\right\} \end{aligned}$$

are compact for all $R\ge 0$. The pair $\left( E,\,\rho \right)$ is called a $weighted$ $space$. An admissible weight function is clearly lower semicontinuous and attains its minimum.

As proved in the subsequent lemma, the product space of weighted spaces is again a weighted space. This will be essential for the proof of the existence of generalized Feller processes in Theorem 3.3.

### Lemma 2.1

Let $\left( E_{i},\rho _{i}\right)$, $i\in \left\{ 1, \ldots ,n\right\}$ be weighted spaces. Then

$$\begin{aligned} \left( E_{1}\times \cdots \times E_{n},\rho \right) \end{aligned}$$

is a weighted space, where

$$\begin{aligned} \rho \left( x_{1}, \ldots ,x_{n}\right) :=\rho _{1}\left( x_{1}\right) \cdot \cdot \cdot \rho _{n}\left( x_{n}\right) . \end{aligned}$$

### Proof

It is well known (see, e.g., [34, Proposition 6.14]) that the product space $E_{1}\times \cdots \times E_{n}$ is completely regular. Without loss of generality, let $\rho _{i}\ge 1$ for $i\in \left\{ 1, \ldots ,n\right\}$ and let $R>0$ be arbitrary. Then

$$\begin{aligned}&\left\{ \left( x_{1}, \ldots ,x_{n}\right) \in E_{1}\times \cdots \times E_{n}:\,\rho _{1}\left( x_{1}\right) \cdot \cdot \cdot \rho _{n}\left( x_{n}\right) \le R\right\} \\&\subset \left\{ x_{1}\in E_{1}:\,\rho _{1}\left( x_{1}\right) \le R\right\} \times \cdots \times \left\{ x_{n}\in E_{n}:\,\rho _{n}\left( x_{n}\right) \le R\right\} . \end{aligned}$$

Since the right-hand side is compact, we only need to show closedness of the left-hand side. For $y=\left( y_{1}, \ldots ,y_{n}\right)$ such that $\rho \left( y_{1}, \ldots ,y_{n}\right) >R$, by lower semicontinuity of $\rho _{1}$, ...$\rho _{n}$ for any $\varepsilon >0$ there exist open neighborhoods $U_{y_{1}}^{\varepsilon }\subset E_{1}$ of $y_{1}$, ..., $U_{y_{n}}^{{\varepsilon }}\subset E_{n}$ of $y_{n}$ such that for any $u_{i}\in U_{y_{i}}^{{\varepsilon }}$, $i\in \left\{ 1, \ldots ,n\right\}$

$$\begin{aligned} \rho _{i}(u_{i})>\rho _{i}(y_{i})-\varepsilon . \end{aligned}$$

Therefore, for $$u\in U_{y_{1}}^{\varepsilon }\times \cdots \times U_{y_{n}}^{{\varepsilon }}$$

$$\begin{aligned} \rho (u)>\left( \rho _{1}(y_{1})-\varepsilon \right) \cdot \cdot \cdot \left( \rho _{n}(y_{n})-\varepsilon \right) \end{aligned}$$

and the right-hand side is larger than *R* for $\varepsilon$ small enough. $\square$

Following [19], for an admissible weight function $\rho$, a Banach space *Z* and $f:\,E\rightarrow Z$ we define the map

$$\begin{aligned} \left\| \cdot \right\| _{\rho }:\,f\rightarrow \underset{x\in E}{\sup }\,\frac{\left\| f(x)\right\| }{\rho (x)} \end{aligned}$$

and

$$\begin{aligned} B^{\rho }(E;Z):=\left\{ f:\,E\rightarrow Z:\,\left\| f(x)\right\| _{\rho } <\infty \right\} . \end{aligned}$$

By standard arguments, it follows that $B^{\rho }(E;Z)$ is a Banach space with respect to the norm $\left\| \cdot \right\| _{\rho }$. Furthermore, we set

$$\begin{aligned} {\mathscr {B}}^{\rho }(E;Z):= \overline{C_{b}\left( E,Z\right) }^{\rho } \end{aligned}$$

and ${\mathscr {B}}^{\rho }(E):= {\mathscr {B}}^{\rho }(E;\mathbb {R})$. The elements of ${\mathscr {B}}^{\rho }(E)$ are called *functions with growth controlled by *$\rho$.

It was proved in [19] that the space ${\mathscr {B}}^{\rho }(E)$ is closely related to the space of continuous maps on a compact space. (Here, we use the convention $\sup \emptyset = 0$.)

### Theorem 2.2

Let $f:\,E\rightarrow \mathbb {R}$. Then $f\in {\mathscr {B}}^{\rho }(E)$ if and only if

(i) for all $$R>0$$

$$\begin{aligned} \left. f\right| _{K_{R}}\in C_{b}(K_{R},\mathbb {R}), \end{aligned}$$

and

(ii)

$$\begin{aligned} \underset{R\rightarrow \infty }{\lim }\underset{x\in E\setminus K_{R}}{\sup }\frac{\left| f(x)\right| }{\rho (x)}=0. \end{aligned}$$

In case of continuous admissible weight functions this relationship can be further specified, as stated in Lemma 2.3. This will be in important in Sect. 5 when we establish a relation to Feller processes. In particular, continuity of $\rho$ automatically implies local compactness of *E*.

### Lemma 2.3

If the admissible weight function $\rho$ is continuous, then

(i) E is locally compact,

(ii) $${\mathscr {B}}^{\rho }(E)\subset C(E)$$,

(iii) $$f\in C_{0}(E)$$ implies $$f\cdot \rho \in {\mathscr {B}}^{\rho }(E)$$,

(iv) $$f\in {\mathscr {B}}^{\rho }(E)$$ implies $$\frac{f}{\rho }\in C_{0}(E)$$.

### Proof

(i) If $\rho$ is continuous, then every point has a compact neighborhood of type $\{\rho \le R \}$ for some $R > 0$, whence E is locally compact (for a converse statement under convexity see [18, Remark 2.2]).
(ii) For $f\in {\mathscr {B}}^{\rho }(E)$ by the definition of ${\mathscr {B}}^{\rho }(E)$, $\frac{f}{\rho }$ is the uniform limit of $\left( \frac{g_{n}}{\rho }\right) _{n\in \mathbb {N}}$ for some $\left( g_{n}\right) _{n\in \mathbb {N}}\subset C_{b}(E)$. Hence, $\frac{f}{\rho }$ is continuous and therefore also *f*.
(iii) If $f\in C_{0}(E)$, then $f\cdot \rho$ is continuous and $\underset{n\in \mathbb {N}}{\bigcup }\left\{ \rho <n\right\}$ is an open cover of *E*; hence, for any $\varepsilon >0$ finitely many such sets suffice to cover the compact set $\left\{ \left| f\right| \ge \varepsilon \right\}$. Thus, for any $\varepsilon >0$ there exists $R_{\varepsilon }>0$ such that $\left| f\right| <\varepsilon$ on $E\setminus K_{R_{\varepsilon }}$ and by Theorem 2.2$f\cdot \rho \in {\mathscr {B}}^{\rho }(E)$.
(iv) From (i), it follows that $\frac{f}{\rho }$ is continuous. By Theorem 2.2, for any $\varepsilon >0$ there is some $R'_{\varepsilon }>0$ such that $\left\{ \left| \frac{f}{\rho }\right| \ge \varepsilon \right\} \subset K_{R'_{\varepsilon }}$. Therefore, by closedness $\left\{ \left| \frac{f}{\rho }\right| \ge \varepsilon \right\}$ is compact and
  $$\begin{aligned} \frac{f}{\rho }\in C_{0}(E). \end{aligned}$$

$$\square $$

The following Riesz representation theorem was proved in [19]. Here we add the observation that the precise statement of [10, §5 Proposition 5] that was used in the proof also yields the uniqueness of the signed Radon measure. This allows one to characterize the dual space of ${\mathscr {B}}^{\rho }(E)$.

### Theorem 2.4

(Riesz representation for ${\mathscr {B}}^{\rho }(E)$) Let $\ell :{\mathscr {B}}^{\rho }(E)\rightarrow \mathbb {R}$ be a continuous linear map. Then, there exists a unique signed Radon measure $\mu : \mathcal {B}(E) \rightarrow [-\infty , \infty ]$ such that

2.1 $$\begin{aligned} \ell (f)=\int _{E}f(x)\mu (dx)  &   \text {for all }f\in {\mathscr {B}}^{\rho }(E). \end{aligned}$$

Additionally,

$$\begin{aligned} \left\| \ell \right\| _{L\left( {\mathscr {B}}^{\rho }(E),\mathbb {R}\right) }=\int _{E}\rho (x)\left| \mu \right| (dx). \end{aligned}$$

Conversely, for any signed Radon measure $\mu$ for which

$$\begin{aligned} \int _{E}\rho (x)\left| \mu \right| (dx) < \infty \end{aligned}$$

holds, the linear map ${\mathscr {B}}^{\rho }(E) \rightarrow \mathbb {R}, \quad f \rightarrow \int _{E}f(x)\mu (dx)$ is continuous.

### Definition 2.5

We denote the space of signed Radon measures satisfying the condition in the last display of Theorem 2.4 by $\mathcal {M}^{\rho }(E)$. This characterizes the dual space of ${\mathscr {B}}^{\rho }(E)$.

The above results, in particular Theorem 2.2, show that the space ${\mathscr {B}}^{\rho }(E)$ is closely related to continuous functions on compacts. For these kinds of function spaces, the Stone–Weierstrass theorem holds true. We now show that a weighted version thereof also holds for ${\mathscr {B}}^{\rho }(E).$

### Proposition 2.6

(Stone–Weierstrass for ${\mathscr {B}}^{\rho }(E)$)

Let $A\subset C_{b}(E)$ be an algebra with respect to pointwise multiplication that contains $1_{E}$ and that separates points. Then *A* is dense in ${\mathscr {B}}^{\rho }(E)$ with respect to $\left\| \cdot \right\| _{\rho }$.

### Proof

The idea of the proof is to approximate elements of ${\mathscr {B}}^{\rho }(E)$ by continuous bounded maps on *E* which in turn can be approximated on $K_{R}$ for any $R>0$ via the standard Stone–Weierstrass theorem [33] by elements in *A* that are restricted to $K_{R}$. However, such an element in *A*, albeit bounded, may have an arbitrary large bound that depends on *R*. Thus, it may not approximate with respect to $\left\| \cdot \right\| _{\rho }$ on all of *E*. Therefore, it is rescaled by a suitable polynomial such that an element in *A* is obtained whose bounds do not depend on *R*. This yields an approximation with respect to $\left\| \cdot \right\| _{\rho }$.

In the following, this idea will be made rigorous. Let $h\in {\mathscr {B}}^{\rho }(E)$ and $\varepsilon >0$. By definition of ${\mathscr {B}}^{\rho }(E)$, there exists $g_{\varepsilon }\in C_{b}(E)$ such that

$$\begin{aligned} \left\| g_{\varepsilon }-h\right\| _{\rho }<\varepsilon . \end{aligned}$$

Set

$$\begin{aligned} R_{\varepsilon }:=\max \left( \frac{\left\| g_{\varepsilon }\right\| _{\infty }}{\varepsilon },1\right) . \end{aligned}$$

The set $A_{\varepsilon }\subset C_{b}(K_{R_{\varepsilon }})$ defined as

$$\begin{aligned} A_{\varepsilon }:=\left\{ \left. f\right| _{K_{R_{\varepsilon }}}:\,f\in A\right\} \end{aligned}$$

is an algebra that contains $1_{K_{R_{\varepsilon }}}$ and that separates points; hence, by the standard Stone–Weierstrass theorem $A_{\varepsilon }$ is dense in $C(K_{R_{\varepsilon }}).$ Thus, there is $f_{\varepsilon }\in A$ such that

$$\begin{aligned} \underset{x\in K_{R_{\varepsilon }}}{\sup }\left| f_{\varepsilon }(x)-g_{\varepsilon }(x)\right| <\varepsilon . \end{aligned}$$

Clearly,

$$\begin{aligned} \alpha _{\varepsilon }:=\underset{x\in K_{R_{\varepsilon }}}{\sup }\left| f_{\varepsilon }(x)\right| \le \underset{x\in E}{\sup }\left| g_{\varepsilon }(x)\right| +\varepsilon =:\beta _{\varepsilon }. \end{aligned}$$

Set

$$\begin{aligned} \gamma _{\varepsilon }:=\underset{x\in E}{\sup }\left| f_{\varepsilon }(x)\right| . \end{aligned}$$

By the Tietze–Urysohn theorem (see, e.g., [28]), there exists a continuous map

$$\begin{aligned} \varphi _{\varepsilon }:\,\left[ -\gamma _{\varepsilon },\gamma _{\varepsilon }\right] \rightarrow \left[ -\beta _{\varepsilon },\beta _{\varepsilon }\right] \end{aligned}$$

such that

$$\begin{aligned} \varphi _{\varepsilon }(y)={\left\{ \begin{array}{ll} y &  \text {for }y\in \left[ -\alpha _{\varepsilon },\alpha _{\varepsilon }\right] \\ \beta _{\varepsilon } &  \text {for }\left| y\right| \ge \beta _{\varepsilon }. \end{array}\right. } \end{aligned}$$

Again by the standard Stone–Weierstrass theorem, on a compact set the space of polynomials is dense in the space of continuous maps. This means that there is a polynomial $p_{\varepsilon }$ on $\left[ -\gamma _{\varepsilon },\gamma _{\varepsilon }\right]$ such that

$$\begin{aligned} \underset{y\in \left[ -\gamma _{\varepsilon },\gamma _{\varepsilon }\right] }{\sup }\left| p_{\varepsilon }(y)-\varphi _{\varepsilon }(y)\right| <\varepsilon ; \end{aligned}$$

hence,

$$\begin{aligned} \underset{x\in E}{\sup }\left| \frac{\left( p_{\varepsilon }\circ f_{\varepsilon }\right) (x)-\left( \varphi _{\varepsilon }\circ f_{\varepsilon }\right) (x)}{\rho (x)}\right| \le \frac{\varepsilon }{\underset{x\in E}{\inf }\rho (x)}. \end{aligned}$$

Since *A* is an algebra $p_{\varepsilon }\circ f_{\varepsilon }\in A$ and

$$\begin{aligned}&\left\| h-p_{\varepsilon }\circ f_{\varepsilon }\right\| _{\rho } \le \left\| h-g_{\varepsilon }\right\| _{\rho }+\left\| g_{\varepsilon }-\varphi _{\varepsilon }\circ f_{\varepsilon }\right\| _{\rho }+\left\| \varphi _{\varepsilon }\circ f_{\varepsilon }-p_{\varepsilon }\circ f_{\varepsilon }\right\| _{\rho }\\&\quad \le \varepsilon +\underset{x\in K_{R_{\varepsilon }}}{\sup }\left| \frac{g_{\varepsilon }(x)-\left( \varphi _{\varepsilon }\circ f_{\varepsilon }\right) (x)}{\rho (x)}\right| \\&\qquad +\underset{x\in E\setminus K_{R_{\varepsilon }}}{\sup }\left| \frac{g_{\varepsilon }(x)-\left( \varphi _{\varepsilon }\circ f_{\varepsilon }\right) (x)}{\rho (x)}\right| +\frac{\varepsilon }{\underset{x\in E}{\inf }\rho (x)}\\&\quad \le \varepsilon +\underset{x\in K_{R_{\varepsilon }}}{\sup }\left| \frac{g_{\varepsilon }(x)-f_{\varepsilon }(x)}{\rho (x)}\right| \\&\qquad +2\,\underset{x\in E}{\sup }\left| \frac{\left| g_{\varepsilon }(x)\right| +\varepsilon }{R_{\varepsilon }}\right| +\frac{\varepsilon }{\underset{x\in K_{R_{\varepsilon }}}{\inf }\rho (x)}\\&\quad \le \varepsilon +\frac{\varepsilon }{\underset{x\in K_{R_{\varepsilon }}}{\inf }\rho (x)}+2\left( \varepsilon +\varepsilon \right) +\frac{\varepsilon }{\underset{x\in K_{R_{\varepsilon }}}{\inf }\rho (x)}, \end{aligned}$$

and *A* is dense in ${\mathscr {B}}^{\rho }(E)$ . $\square$

We now turn to $$ generalized Feller semigroup s$$, which have been introduced by Röckner and Sobol in [31] in a specific setting which in turn has been extended in [19] to general $${\mathscr {B}}^{\rho }(E)$$-spaces.

### Definition 2.7

Let $\left( P(t)\right) _{t\in \mathbb {R}_{+}}$ be a family of bounded linear operators on ${\mathscr {B}}^{\rho }(E)$. We call the family $\left( P(t)\right) _{t\in \mathbb {R}_{+}} generalized$ $Feller semigroup$ on ${\mathscr {B}}^{\rho }(E)$ if the following conditions hold true:

**P1** $$P(0)=\text {Id}$$, on $${\mathscr {B}}^{\rho }(E)$$,

**P2 **   $$ P(t+s)=P(s)\circ P(t) \,\,$$ for all $$ s,t\in \mathbb {R}_{+}$$,

**P3** for all $f\in {\mathscr {B}}^{\rho }(E)$ and all $x\in E$

$$\begin{aligned} \underset{t\searrow 0}{\lim }\,P(t)f(x)=f(x), \end{aligned}$$

**P4** there exist $\varepsilon >0$ and $C>0$ such that for all $t\in \left[ 0,\varepsilon \right]$

$$\begin{aligned} \left\| P(t)\right\| _{L\left( {\mathscr {B}}^{\rho }(E)\right) }\le C, \end{aligned}$$

**P5** *P*(*t*) is a positive linear operator for all $t\in \mathbb {R}_{+}$.

As proved in [19], this definition yields strong continuity, as stated in the next theorem.

### Theorem 2.8

Let $\left( P(t)\right) _{t\in \mathbb {R}_{+}}$ be a generalized Feller semigroup on ${\mathscr {B}}^{\rho }(E)$. Then $\left( P(t)\right) _{t\in \mathbb {R}_{+}}$ is strongly continuous on ${\mathscr {B}}^{\rho }(E)$.

Since we know the dual space of ${\mathscr {B}}^{\rho }(E)$, we can connect generalized Feller semigroups to a family of positive finite Radon measures on $\left( E,\mathcal {B}(E)\right)$.

### Lemma 2.9

Let $\left( P(t)\right) _{t\in \mathbb {R}_{+}}$ be a generalized Feller semigroup on ${\mathscr {B}}^{\rho }(E)$. Then there exists a unique family of positive finite Radon measures

$$\begin{aligned} \left( p(t)(x,\cdot )\right) _{t\in \mathbb {R}_{+},x\in E} \end{aligned}$$

on $\left( E,\mathcal {B}(E)\right)$ such that for all $x\in E$, $t\in \mathbb {R}_{+}$ and $f\in {\mathscr {B}}^{\rho }(E)$

$$\begin{aligned} P(t)f(x)=\int _{E}f(y)p(t)(x,dy), \end{aligned}$$

and $$p(t)(x,\cdot )\in \mathcal {M^{\rho }}(E)$$.

### Proof

By the definition of generalized Feller semigroups, for any $t\in \mathbb {R}_{+}$ and $x\in E$ the map

$$\begin{aligned} {\mathscr {B}}^{\rho }(E)&\rightarrow \mathbb {R}\\ \ell _{t,x}:\,f&\rightarrow P(t)f(x) \end{aligned}$$

is positive, linear and continuous. Thus, by Theorem 2.4 for any $t\in \mathbb {R}_{+}$ and any $x\in E$ there is a unique positive finite Radon measure $p(t)(x,\cdot )\in \mathcal {M}^{\rho }(E)$ such that

$$\begin{aligned} \left( P(t)f\right) (x)=\int _{E}f(y)p(t)(x,dy) \end{aligned}$$

holds true. $$\square $$

Let us also recall the following observation made in [18] Remark 2.8.

### Remark 2.10

Let $\left( P(t)\right) _{t\in \mathbb {R}_{+}}$ be a generalized Feller semigroup on ${\mathscr {B}}^{\rho }(E)$. Then it follows from standard semigroup theory that there exists some $M\ge 1$ and $\omega \in \mathbb {R}$ such that for any $t\in \mathbb {R}_{+}$

$$\begin{aligned} \left\| P(t)\right\| _{L\left( {\mathscr {B}}^{\rho }(E)\right) } \le Me^{\omega t}. \end{aligned}$$

For all $x\in E$ and $t\in \mathbb {R}_{+}$, then one obtains the bounds

$$\begin{aligned} P(t)\rho (x)=\underset{\begin{array}{c} f\in C_{b}(E)\\ \left| f\right| \le \rho \end{array}}{\sup }\left| \left( P(t)f\right) (x)\right| \le \rho (x)\left\| P(t)\right\| _{L\left( {\mathscr {B}}^{\rho }(E)\right) }\le \rho (x)Me^{\omega t}. \end{aligned}$$

Notice that $\rho$ appears to be an $\omega$-superaveraging function in the terminology of [11] if $M=1$.

The following proposition states that with respect to the Baire $\sigma$-algebra $\mathcal {B}_{0}(E)$ (see Definition A.3) the family of positive finite Radon measures from Lemma 2.9 turns out to be a semigroup of transition kernels.

### Proposition 2.11

Let $\left( P(t)\right) _{t\in \mathbb {R}_{+}}$ be a generalized Feller semigroup on ${\mathscr {B}}^{\rho }(E)$.

The family $\left( \hat{p}(t)\right) _{t\in \mathbb {R}_{+}}$ defined as the restriction

$$\begin{aligned} \hat{p}(t)=\left. p(t)\right| _{E\times \mathcal {B}_{0}(E)}  &   \text { for } t\ge 0 \end{aligned}$$

is a semigroup of transition kernels on $(E, \mathcal {B}_{0}(E))$, i.e., with respect to the Baire $\sigma$-algebra.

### Remark 2.12

Note that if *E* is metrizable, the Borel and the Baire $\sigma$-algebra coincide (see Corollary 6.3.5 in [8]). Hence, in this case $\left( p(t)\right) _{t\in \mathbb {R}_{+}}$ is also a semigroup of transition kernels on $(E, \mathcal {B}(E))$, i.e., with respect to the Borel $\sigma$-algebra. Another instance when the Borel and the Baire $\sigma$-algebra coincide is the case where *E* is a (not necessarily metrizable) Lusin space (i.e., a space which is measurably isomorphic to a Borel set of a complete separable metric space) that contains a countable point separating family of functions from ${\mathscr {B}}^{\rho }(E)$. This statement follows from Lusin’s or Kuratowski’s measurability theorem. For further conditions implying the coincidence of the Borel and the Baire $\sigma$-algebra we refer to Chapter 6.3 of [8].

### Proof

For any $C_{b}(E)$-open set $A\in \mathcal {B}_{0}(E)$ (see Definition A.1), there exists a sequence $\left( f_{n}^{A}\right) _{n\in \mathbb {N}}\subset C_{b}(E)$ such that $f_{n}^{A}\nearrow 1_{A}$ pointwise. Hence, by dominated convergence for any $t\in \mathbb {R}_{+}$

$$\begin{aligned} x\rightarrow p(t)(x,A)=\underset{n\rightarrow \infty }{\lim }P(t)f_{n}^{A}(x) \end{aligned}$$

is measurable with respect to Baire $\sigma$-algebra $\mathcal {B}_{0}(E)$ as limit of maps that lie in ${\mathscr {B}}^{\rho }(E)$ and are therefore Baire-measurable (by virtue of being pointwise limits of $C_{b}(E)$ functions). This property extends to all sets *A* in the Dynkin system generated by the $C_{b}(E)$-open sets. Since the system of $C_{b}(E)$-open sets is intersection stable, the property holds true also for the $\sigma$-algebra generated by the $C_{b}(E)$-open sets. By Lemma A.2, this is precisely $\mathcal {B}_{0}(E)$.

Furthermore, for any $C_{b}(E)$-open set *A* and any $s,t\in \mathbb {R}_{+}$ by dominated convergence

$$\begin{aligned} \int _{E}p(s)(y,A)p(t)(x,dy)&=\underset{n\rightarrow \infty }{\lim }P(t)P(s)f_{n}^{A}\\&=\underset{n\rightarrow \infty }{\lim }P(s+t)f_{n}^{A}\\&=p(s+t)(y,A). \end{aligned}$$

Since the system of sets such that this equations holds is a Dynkin system, it follows that

$$\begin{aligned} \int _{E}p(s)(y,A)p(t)(x,dy)&=p(s+t)(x,A) \end{aligned}$$

holds true for any $A\in \mathcal {B}_0(E)$. $\square$

## Generalized Feller processes

This section is mainly dedicated to prove existence and path properties of generalized Feller processes. Throughout $\left( E,\,\rho \right)$ denotes a weighted space. We let *I* be some index set and let $J\subset I$ be a finite subset. We denote the number of elements in *J* by |*J*| and consider the product space

$$\begin{aligned} E^{J}:=\underbrace{E \times \cdots \times E}_{|J|-\text {times}}, \end{aligned}$$

whose elements are denoted by $x_{J}:=\left( x_{1},\ldots ,x_{|J|}\right)$. We recall that by Lemma 2.1 , $\left( E^{J},\rho ^{\otimes |J|}\right)$ is a weighted space with $\rho ^{\otimes |J|}(x_{J}):=\rho \left( x_{{1}}\right) \cdots \rho \left( x_{|J|}\right) .$ Let us start by formally introducing generalized Feller processes.

### Definition 3.1

Let $\left( P(t)\right) _{t\in \mathbb {R}_{+}}$ be a generalized Feller semigroup on ${\mathscr {B}}^{\rho }(E)$, let $\nu \in \mathcal {M}^{\rho }(E)$ be a probability measure, and let $\left( \lambda _{t}\right) _{t\in \mathbb {R}_{+}}$ be an adapted *E*-valued stochastic process on the filtered probability space

$$\begin{aligned} \left( E^{\mathbb {R}_{+}},\mathcal {B}(E)^{\mathbb {R}_{+}},\left( \mathcal {F}_{t}\right) _{t\in \mathbb {R}_{+}},\mathbb {P}_{\nu }\right) . \end{aligned}$$

If for any $t\ge s\ge 0$ and any $f\in {\mathscr {B}}^{\rho }(E)$

3.1 $$\begin{aligned} \mathbb {E}_{\nu }\left[ \left. f(\lambda _{t})\right| \mathcal {F}_{s}\right] =P\left( t-s\right) f(\lambda _{s}) \end{aligned}$$

holds true $\mathbb {P}_{\nu }$-almost surely and

$$\begin{aligned} \mathbb {P}_{\nu }\circ \lambda _{0}^{-1}=\nu \end{aligned}$$

then $\left( \lambda _{t}\right) _{t\in \mathbb {R}_{+}}$ is called a $generalized$ $Feller process$ with respect to $\left( \mathcal {F}_{t}\right) _{t\in \mathbb {R}_{+}}$ and $\left( P(t)\right) _{t\in \mathbb {R}_{+}}$ with initial distribution $\nu$.

We make the convention $\mathbb {P}_{x}:=\mathbb {P}_{\delta _{x}}$ for any $x\in E$.

### Remark 3.2

Note that (3.1) is only required to hold for $f\in \mathscr {B}^{\rho }(E)$, thus only for Baire-measurable functions and therefore in general not necessarily for Borel-measurable maps *f*. This is due to the fact that indicator functions of Borel sets can be approximated with continuous bounded functions by Corollary A.8 only almost everywhere with respect to one (or countably many) measure(s), but not necessarily simultaneously with respect to the entire family of measures $\left( \left( p(t-s)\right) (x,\cdot )\right) _{\,x\in E}$ on $\left( E,\mathcal {B}(E)\right)$ obtained by Lemma 2.9. However, for indicator functions of $C_{b}(E)$-open sets (see Definition A.1) Eq. (3.1) holds true by dominated convergence. Since by Lemma A.2 the $C_{b}(E)$-open sets generate the Baire $\sigma$-algebra $\mathcal {B}_{0}(E)$ (see Definition A.3), we conclude again by dominated convergence that Eq. (3.1) holds true for any indicator function of sets in $\mathcal {B}_{0}(E)$. Thus, in this sense a generalized Feller process $\left( \lambda _{t}\right) _{t\in \mathbb {R}_{+}}$ with initial distribution $\nu$ is a Markov process only with respect to the measurable space $(E^{\mathbb {R}_{+}},\mathcal {B}_{0}(E)^{\mathbb {R}_{+}}),$ and the probability measure $\mathbb {P}_{\nu }$ restricted to this space.

In cases where the Borel and the Baire $\sigma$-algebra coincide (such as for metrizable spaces, see Remark 2.12), there is of course no difference.

One important result of the current paper is the following proof of the existence of generalized Feller processes. A similar statement was already formulated in [18, Theorem 2.11], but the full proof with all details is given below.

### Theorem 3.3

Let $\left( P(t)\right) _{t\in \mathbb {R}_{+}}$ be a generalized Feller semigroup on ${\mathscr {B}}^{\rho }(E)$ such that for all $t\in \mathbb {R}_{+}$

$$\begin{aligned} P(t)1=1. \end{aligned}$$

Then on the measurable space $(E^{\mathbb {R}_{+}},\mathcal {B}(E)^{\mathbb {R}_{+}})$, for any probability measure $\nu \in \mathcal {M}^{\rho }(E)$ there exists a measure $\mathbb {P}_{\nu }$ and a right continuous filtration $\left( \mathcal {F}_{t}\right) _{t\in \mathbb {R}_{+}}$, e.g., the right continuous version of the natural filtration of the canonical process $\left( \lambda _{t}\right) _{t\in \mathbb {R}_{+}}$, such that $\left( \lambda _{t}\right) _{t\in \mathbb {R}_{+}}$ is adapted with respect to $\left( \mathcal {F}_{t}\right) _{t\in \mathbb {R}_{+}}$, and for any $t\ge s\ge 0$ and any $f\in {\mathscr {B}}^{\rho }(E)$, there exists a set of $\mathbb {P}_{\nu }$-measure 1 such that

3.2 $$\begin{aligned} \mathbb {E}_{\nu }\left[ \left. f(\lambda _{t})\right| \mathcal {F}_{s}\right] =P\left( t-s\right) f(\lambda _{s}) \end{aligned}$$

and

$$\begin{aligned} \mathbb {\mathbb {P}}_{\nu }\circ \lambda _{0}^{-1}=\nu . \end{aligned}$$

When we speak of generalized Feller processes, we mean those obtained via Theorem 3.3. We remind the reader that while $\mathcal {B}(E^{\mathbb {R}_{+}})\supset \mathcal {B}(E)^{\mathbb {R}_{+}}$ holds true (because on $\mathcal {B}(E^{\mathbb {R}_{+}})$ every projection is continuous, hence measurable with respect to $\mathcal {B}(E^{\mathbb {R}_{+}})$), the inclusion $\mathcal {B}(E^{\mathbb {R}_{+}})\subset \mathcal {B}(E)^{\mathbb {R}_{+}}$ is in general not true when the topology of *E* does not have a countable base.

### Proof

The proof has three steps. In the first step, we construct a projective family of probability measures on

$$\begin{aligned} \left( E^{J},\mathcal {B}\left( E^{J}\right) \right) _{J\subset \mathbb {R}_{+},\text { finite}}. \end{aligned}$$

In the second step, we use the generalized Kolmogorov extension theorem (Theorem 15.26 in [3]) and obtain a probability measure on $\left( E^{\mathbb {R}_{+}},\mathcal {B}(E)^{\mathbb {R}_{+}}\right)$. The coordinate process $\left( \lambda _{t}\right) _{t\in \mathbb {R}_{+}}$ on this space then yields for any $t\ge s\ge 0$ and any $f\in {\mathscr {B}}^{\rho }(E)$

3.3 $$\begin{aligned} \mathbb {E}_{\nu }\left[ \left. f(\lambda _{t})\right| \mathcal {F}_{s}^{0}\right] =P\left( t-s\right) f(\lambda _{s}), \end{aligned}$$

where $\left( \mathcal {F}_{t}^{0}\right) _{t\in \mathbb {R}_{+}}$ is the natural filtration of the coordinate process. In the third step, we take the right continuous version of this filtration and show Eq. (3.2)

*Step 1:* Fix some probability measure $\nu \in \mathcal {M}^{\rho }(E)$. For any $r\ge 0$, let $p_{\nu }(r)(\cdot )$ be the unique measure in $\mathcal {M}^{\rho }(E)$ given by Theorem 2.4 via

$$\begin{aligned} \int _{E}P(r)f(y)\nu (dy)=\int _{E}f(y)p_{\nu }(r)(dy). \end{aligned}$$

Using the Riesz representation theorem on $E\times E$ and Lemma A.9, we define, for $0\le r_{1}<r_{2}$ and $R >0$, the unique measure $\mu _{\nu }^{R,\left\{ r_{1},r_{2}\right\} }\in \mathcal {M}^{\rho ^{\otimes 2}}(E\times E)$ via the continuous functional

$$\begin{aligned} f_{r_{1}}\cdot f_{r_{2}}&\rightarrow \int _{E}\left( 1_{\left\{ \rho (y)<R\right\} }\cdot f_{r_{1}}(y)\cdot P(r_{2}-r_{1})f_{r_{2}}(y)\right) p_{\nu }(r_{1})(dy) \end{aligned}$$

for $f_{r_{1}}, f_{r_2} \in \mathscr {B}^{\rho }(E)$ such that

$$\begin{aligned}&\int _{E}\left( 1_{\left\{ \rho (y)<R\right\} }\cdot f_{r_{1}}(y)\cdot P(r_{2}-r_{1})f_{r_{2}}(y)\right) p_{\nu }(r_{1})(dy)\\&\quad = \,\int _{E\times E}f_{r_{1}}(y)f_{r_{2}}(z)\mu _{\nu }^{R,\left\{ r_{1},r_{2}\right\} }(dy,dz). \end{aligned}$$

By $P(r_{2}-r_{1})1=1$, we obtain

$$\begin{aligned} \mu _{\nu }^{R,\left\{ r_{1},r_{2}\right\} }(E\times E)&=p_{\nu }(r_{1})\left( \left\{ \rho (y)<R\right\} \right) \le p_{\nu }(r_{1})(E) = \nu (E) = 1. \end{aligned}$$

Then for any $A\in \mathcal {B}\left( E\times E\right)$ by monotonicity and boundedness, we can define

$$\begin{aligned} p_{\nu }^{\left\{ r_{1},r_{2}\right\} }(A):=\underset{R\rightarrow \infty }{\lim }\mu _{\nu }^{R,\left\{ r_{1},r_{2}\right\} }(A). \end{aligned}$$

One can easily show that the convergence is uniform in the sets *A* (i.e., we obtain convergence in total variation) and that $p_{\nu }^{\left\{ r_{1},r_{2}\right\} }$ is a probability measure on $\left( E\times E,\mathcal {B}\left( E\times E\right) \right) .$ Furthermore, for any $r_{3}>r_{2}$ by Lemma A.9 we can define a continuous functional $j^{R,\left\{ r_{1},r_{2},r_{3}\right\} }$ on ${\mathscr {B}}^{\rho ^{\otimes 3}}(E\times E\times E)$ by setting for any $f_{r_{i}}\in {\mathscr {B}}^{\rho }(E)$, $i=1,2,3$

$$\begin{aligned} f_{r_{1}}\cdot f_{r_{2}}\cdot f_{r_{3}}\rightarrow &   \int _{E_{r_{1}}\times E_{r_{2}}}1_{\left\{ \rho _{r_{1}}(y)<R\right\} }\cdot 1_{\left\{ \rho _{r_{2}}(z)<R\right\} }\cdot f_{r_{1}}(y)\cdot f_{r_{2}}(z)\\    &   \times \cdot P(r_{3}-r_{2})f_{r_{3}}(z)p_{\nu }^{\left\{ r_{1},r_{2}\right\} }(dy,dz). \end{aligned}$$

Then again by the Riesz representation theorem, we define $\mu _{\nu }^{R,\left\{ r_{1},r_{2},r_{3}\right\} }$ as the unique measure in $\mathcal {M}^{\rho ^{\otimes 3}}(E\times E\times E)$ such that for any $f\in {\mathscr {B}}^{\rho ^{\otimes 3}}(E\times E\times E)$

$$\begin{aligned} j^{R,\left\{ r_{1},r_{2},r_{3}\right\} }(f)=\int _{E\times E \times E}f(x,y,z)\mu _{\nu }^{R,\left\{ r_{1},r_{2},r_{3}\right\} }(dy,dy,dz). \end{aligned}$$

Again for any $A\in \mathcal {B}\left( E\times E\times E\right)$ by monotonicity and boundedness

$$\begin{aligned} p_{\nu }^{\left\{ r_{1},r_{2},r_{3}\right\} }(A):=\underset{R\rightarrow \infty }{\lim }\mu _{\nu }^{R,\left\{ r_{1},r_{2},r_{3}\right\} }(A). \end{aligned}$$

Proceeding inductively in this way, we can define via $\mu _{\nu }^{R,J}\in \mathcal {M}^{\rho ^{\otimes |J|}}(E^{J})$ a family of probability measures $\left( p_{\nu }^{J}\right) _{J\subset \mathbb {R}_{+},\,\text {finite }}$ on the respective measurable spaces

$$\begin{aligned} \left( E^{J},\mathcal {B}\left( E^{J}\right) \right) _{J\subset \mathbb {R}_{+},\,\text {finite }}. \end{aligned}$$

Again, one can easily show that the convergence of $\mu _{\nu }^{R,J}$ to $p_{\nu }^{J}$ is uniform for each finite $J\subset \mathbb {R}_{+}$. Using this property, inner regularity of the measure $p_{\nu }^{J}$ for each finite $J\subset \mathbb {R}_{+}$ follows from inner regularity of $\mu _{\nu }^{R,J}\in \mathcal {M}^{\rho ^{\otimes |J|}}(E^{J})$ for each $R>0$ and each finite $J\subset \mathbb {R}_{+}$. Hence, $p_{\nu }^{J}$ is a Radon measure.

In order to apply the generalized Kolmogorov extension theorem (Theorem 15.26 in [3]), we need to show that this family is projective, i.e., for any finite *J* and $i\in J$ and any $A\in \mathcal {B}\left( E^{J\setminus \left\{ i\right\} } \right)$

$$\begin{aligned} p_{\nu }^{J}(A\times E)=p_{\nu }^{J\setminus \left\{ i\right\} }(A). \end{aligned}$$

We show this property by induction and start with the case $J=\left\{ r_{1},r_{2}\right\}$. For $f\in C_{b}(E)$

$$\begin{aligned} \int _{E}f(y)\mu _{\nu }^{R,\left\{ r_{1},r_{2}\right\} }(dy \times E)=\int _{E}f(y)1_{K_{R}}(y)p_{\nu }(r_{1})(dy), \end{aligned}$$

which implies by uniqueness of the Radon measure (see Proposition A.4) for any $A\in \mathcal {B}\left( E\right)$

$$\begin{aligned} \mu _{\nu }^{R,\left\{ r_{1},r_{2}\right\} }(A \times E)=p_{\nu }(r_{1})(A\cap K_{R}). \end{aligned}$$

Thus,

$$\begin{aligned} p_{\nu }^{\left\{ r_{1},r_{2}\right\} }(A\times E)&=\underset{R\rightarrow \infty }{\lim }\mu _{\nu }^{R,\left\{ r_{1},r_{2}\right\} }(A\times E) =p_{\nu }(r_{1})(A). \end{aligned}$$

Furthermore, by interchangeability of the limits due to the uniform convergence of the measures $(\mu _{\nu }^{R,\left\{ r_{1},r_{2}\right\} })_R$ and dominated convergence we get for any $f\in C_{b}(E)$

$$\begin{aligned} \int _{E \times E}1(y)f(z)p_{\nu }^{\left\{ r_{1},r_{2}\right\} }(dy,dz)&= \,\underset{R\rightarrow \infty }{\lim }\int _{E\times E}1(y)f(z)\mu _{\nu }^{R,\left\{ r_{1},r_{2}\right\} }(dy,dz)\\&= \,\underset{R\rightarrow \infty }{\lim }\int _{E}1_{\left\{ \rho (y)<R\right\} }P(r_{2}-r_{1})f(y)p_{\nu }(r_{1})(dy) \\&= \,P(r_{1})P(r_{2}-r_{1})f(x)= \,P(r_{2})f(x)\\  &= \,\int _{E}f(z)p_{\nu }(r_{2})(dz). \end{aligned}$$

Since the functionals $f \rightarrow \int _{E}f(z)p_{\nu }^{\left\{ r_{1},r_{2}\right\} }(E \times dz)$ and $f \rightarrow \int _{E}f(z)p_{\nu }(r_{2})(dz)$ coincide on $C_{b}(E)$ and satisfy the conditions of Proposition A.4, we obtain by uniqueness of the Radon measure that for any $A\in \mathcal {B}\left( E\right)$

$$\begin{aligned} p_{\nu }^{\left\{ r_{1},r_{2}\right\} }(E\times A)=p_{\nu }(r_{2})(A). \end{aligned}$$

This implies in particular that for any $f_{r_{2}}\in \mathscr {B}^{\rho }(E)$

$$\begin{aligned} \int _{E\times E}1(y)f_{r_{2}}(z)p_{\nu }^{\left\{ r_{1},r_{2}\right\} }(dy,dz)<\infty . \end{aligned}$$

Next, we assume that for $N\in \mathbb {N}$, $n\le N$, any arbitrary index set $J_{n}:=\left\{ r_{1}, \ldots ,r_{n}\right\} \in \mathbb {R}_{+}^{n}$ with , $0\le r_{1}< \cdots <r_{n}$, any $i\in \left\{ 1, \ldots ,n\right\}$ and any $A\in \mathcal {B}\left( E^{J_{n}\setminus \left\{ r_{i}\right\} }\right)$ we have

$$\begin{aligned} p_{\nu }^{J_{n}}(A\times E)=p_{\nu }^{J_{n}\setminus \left\{ r_{i}\right\} }(A), \end{aligned}$$

and

$$\begin{aligned} \int _{E^{J_n}}1(y_{1})\cdot ...1(y_{n-1})\cdot f_{r_{n}}(y_{n})p_{\nu }^{J_{n}}(dy_{1},...,dy_{n})<\infty \end{aligned}$$

for any $f_{r_{n}}\in \mathscr {B}^{\rho }(E)$. We want to show the analogous assertions for any $J_{N+1}:=\left\{ r_{1}, \ldots ,r_{N+1}\right\} \in \mathbb {R}_{+}^{N+1}$ with $r_{N+1}> \cdots >r_{1}\ge 0$, for any $i\in \left\{ 1,...,N+1\right\}$ and any $A\in \mathcal {B}\left( E^{J_{N+1}\setminus \left\{ r_{i}\right\} }\right)$. For $i=N+1$ and for $f_{r_{i}}\in C_b(E)$, we have by the definition of the measures and dominated convergence

$$\begin{aligned}&\int _{E^{J_{N+1}}}f_{r_{1}}(y_{1})\cdot ...\cdot f_{r_{N}}(y_{N})1(y_{N+1})p_{\nu }^{J_{N+1}}(dy_{1},...,dy_{N+1})\\&\quad = \underset{R\rightarrow \infty }{\lim }\int _{E^{J_{N+1}}}f_{r_{1}}(y_{1})\cdot ...\cdot f_{r_{N}}(y_{N})1(y_{N+1})\mu _{\nu }^{R,J_{N+1}}(dy_{1},...,dy_{N+1})\\&\quad =\int _{E^{J_{N}}}f_{r_{1}}(y_{1})\cdot ...\cdot f_{r_{N}}(y_{N})p_{\nu }^{J_{N}}(dy_{1},...,dy_{N}), \end{aligned}$$

and we conclude that for any $A\in \mathcal {B}\left( E^{J_{N+1}\setminus \left\{ r_{N+1}\right\} }\right)$

$$\begin{aligned} p_{\nu }^{J_{N+1}}(A\times E)=p_{\nu }^{J_{N+1}\setminus \left\{ r_{N+1}\right\} }(A), \end{aligned}$$

by Proposition A.4. Furthermore,

$$\begin{aligned}&\int _{E^{J_{N+1}}}1(y_{1})\cdot ...1(y_{N})\cdot f_{r_{N+1}}(y_{N+1})p_{\nu }^{J_{N+1}}(dy_{1},...,dy_{N+1})\\&\quad = \int _{E^{J_{N+1}}}1(y_{1})\cdot ...1(y_{N})\cdot P(r_{N+1}-r_{N})f_{r_{N+1}}(y_{N})p_{\nu }^{J_{N}}(dy_{1},...,dy_{N}) < \,\infty , \end{aligned}$$

by assumption.

In case $i=N$ and for $f_{r_{i}}\in C_{b}(E)$ and by the same arguments as above

$$\begin{aligned}&\int _{E^{J_ {N+1}}}f_{r_{1}}(y_{1})\cdot ...\cdot f_{r_{N-1}}(y_{N-1})1(y_{N})f_{r_{N+1}}(y_{N+1})p_{\nu }^{J_{N+1}}(dy_{1},...,dy_{N+1})\\&\quad = \underset{R\rightarrow \infty }{\lim }\int _{E^{J_ {N+1}}}f_{r_{1}}(y_{1})\cdot ...\cdot f_{r_{N-1}}(y_{N-1})\cdot f_{r_{N+1}}(y_{N+1})\mu _{\nu }^{R,J_{N+1}}(dy_{1},...,dy_{N+1})\\&\quad =\int _{{E^{J_{N}}}}f_{r_{1}}(y_{1})\cdot ...\cdot f_{r_{N-1}}(y_{N-1})\cdot P(r_{N+1}-r_{N})f_{r_{N+1}}(y_{N})p_{\nu }^{J_{N}}(dy_{1},...,dy_{N})\\&\quad = \int _{E^{J_{N-1}}}f_{r_{1}}(y_{1})\cdot ...\cdot f_{r_{N-1}}(y_{N-1})\cdot P(r_{N+1}-r_{N-1})f_{r_{N+1}}(y_{N-1})p_{\nu }^{J_{N-1}}(dy_{1},...,dy_{N-1})\\&\quad =\int _{{E^{J_{N}}}}f_{r_{1}}(y_{1})\cdot ...\cdot f_{r_{N-1}}(y_{N-1})\cdot f_{r_{N+1}}(y_{N+1})p_{\nu }^{J_{N+1}\setminus \left\{ r_{N}\right\} }(dy_{1},...,dy_{N-1},dy_{N+1}), \end{aligned}$$

and we can conclude as before. For $i\in \left\{ 1, \ldots ,N-1\right\}$ the desired properties follow in the same way by the definition of $p_{\nu }^{J_{N+1}}$ and from the assumption that the properties hold true for any $n\le N$. Thus, by induction it follows that for any $m\in \mathbb {N}$ and any arbitrary finite index set $J_{m}:=\left\{ r_{1}, \ldots ,r_{m}\right\} \in \mathbb {R}_{+}^{m}$ , $0\le r_{1}< \cdots <r_{m}$ for any $i\in \left\{ 1, \ldots ,m\right\}$ and any $A\in \mathcal {B}\left( E^{J_{m}\setminus \left\{ r_{i}\right\} }\right)$

$$\begin{aligned} p_{\nu }^{J_{m}}(A\times E)=p_{\nu }^{J_{m}\setminus \left\{ r_{i}\right\} }(A). \end{aligned}$$

Therefore, the family $\left( p_{\nu }^{J}\right) _{J\subset \mathbb {R}_{+},\,\text {finite }}$ is projective.

*Step 2:* In order to construct a measure $\mathbb {P}_{\nu }$ on $\left( E^{\mathbb {R}_{+}},\mathcal {B}(E)^{\mathbb {R}_{+}}\right)$ for any $\nu \in \mathcal {M}^{\rho }(E)$, we shall use Theorem 15.26 in [3]. For this purpose, we have to find a compact class (see Definition A.5) $\mathcal {C}$ in *E* such that for each $t\in \mathbb {R}_{+}$ and $A\in \mathcal {B}(E)$

3.4 $$\begin{aligned} p_{\nu }^{\left\{ t\right\} }(A)=\sup \left\{ p_{\nu }^{\left\{ t\right\} }(C):\,C\subset A\text {\,and }C\in \mathcal {C}\right\} . \end{aligned}$$

We prove this for the class

$$\begin{aligned} \mathcal {C}:=\left\{ C:\,C\text { compact},\,C\subset K_{R}\text { for some }R\ge 0\right\} . \end{aligned}$$

To prove that $\mathcal {C}$ is a compact class, we choose some arbitrary sequence $\left\{ C_{l}\right\} _{l\in \mathbb {N}}\subset \mathcal {C}$ such that

$$\begin{aligned} \underset{l\in \mathbb {N}}{\bigcap }\, C_{l}=\emptyset . \end{aligned}$$

For $C_{1}$, we choose $R_{1}\ge 0$ such that $C_{1}\subset K_{R_{1}}.$ Then $\underset{l\in \mathbb {N}}{\bigcup }\, E{\setminus } C_{l}\supset K_{R_{1}}$ is an open cover of the compact set $K_{R_{1}}$; hence, finitely many sets, say without loss of generality $\left\{ E\setminus C_{2},E\setminus C_{2}, \ldots ,E\setminus C_{m}\right\} ,$ suffice to cover it. Thus,

$$\begin{aligned} \underset{l\in \left\{ 2, \ldots ,m\right\} }{\bigcap }C_{l}\cap K_{R_{1}}=\emptyset \end{aligned}$$

and $C_{1}\subset K_{R_{1}}$ yields that $\underset{l\in \left\{ 1, \ldots ,m\right\} }{\bigcap }C_{l}=\emptyset$. Hence, $\mathcal {C}$ is a compact class. Since $\underset{R\ge 0}{\bigcup }K_{R}=E,$ for any $\varepsilon >0$ and $t\in \mathbb {R}_{+}$ there is some $R_{\varepsilon }\ge 0$ such that

$$\begin{aligned} p_{\nu }^{\left\{ t\right\} }\left( E\right) -p_{\nu }^{\left\{ t\right\} }\left( K_{R_{\varepsilon }}\right) <\varepsilon , \end{aligned}$$

and by inner regularity of the Radon measure $p_{\nu }^{\left\{ t\right\} }$ for any $A\in \mathcal {B}(E)$ there is a compact set $A_{\varepsilon }\subset A$ such that

$$\begin{aligned} p_{\nu }^{\left\{ t\right\} }\left( A\right) -p_{\nu }^{\left\{ t\right\} }\left( A_{\varepsilon }\right) <\varepsilon . \end{aligned}$$

Hence,

$$\begin{aligned} p_{\nu }^{\left\{ t\right\} }\left( A\right) -p_{\nu }^{\left\{ t\right\} }\left( A_{\varepsilon }\cap K_{R_{\varepsilon }}\right) <2\varepsilon , \end{aligned}$$

and Eq. (3.4) holds true. Thus, the conditions of Theorem 15.26 in [3] are satisfied. By applying this theorem, we obtain a probability measure $\mathbb {P}_{\nu }$ on the measurable space $\left( E^{\mathbb {R}_{+}},\mathcal {B}(E)^{\mathbb {R}_{+}}\right)$ such that for any finite $J\subset \mathbb {R}_{+}$ and $A\in \mathcal {B}(E^J)$ the probability is given by

3.5 $$\begin{aligned} \mathbb {P}_{\nu }\left( \left( \Pi _{J}^{\mathbb {R}_{+}}\right) ^{-1}\left( A\right) \right) =p_{\nu }^{J}(A), \end{aligned}$$

with $\Pi _{J}^{\mathbb {R}_{+}}$ being the projection from $E^{\mathbb {R}_{+}}$ on $E^{J}$. Let now $\left( \lambda _{t}\right) _{t\in \mathbb {R}_{+}}:=\left( \Pi _{t}^{\mathbb {R}_{+}}\right) _{t\in \mathbb {R}_{+}}$ be the coordinate process on $\left( E^{\mathbb {R}_{+}},\mathcal {B}(E)^{\mathbb {R}_{+}}\right)$. Then, by definition

$$\begin{aligned} \mathbb {P}_{\nu }\circ \left( \lambda _{0}\right) ^{-1}=p_{\nu }^{\left\{ 0\right\} }=\nu , \end{aligned}$$

and for any $f\in {\mathscr {B}}^{\rho }(E)$

$$\begin{aligned} \mathbb {E}_{\nu }\left[ f(\lambda _{t})\right] =\int _{E}\left( P(t)f\right) (y)\nu (dy)<\infty . \end{aligned}$$

We now denote by $\left( \mathcal {F}_{t}^{0}\right) _{t\in \mathbb {R}_{+}}$ the natural filtration of $(\lambda _t)_{t \in \mathbb {R}_+}$ and show Eq. (3.3), i.e., 

$$\begin{aligned} \mathbb {E}_{\nu }\left[ f(\lambda _{t})\cdot 1_{F}\right] =\mathbb {E}_{\nu }\left[ P\left( t-s\right) f(\lambda _{s})\cdot 1_{F}\right] \end{aligned}$$

for any $f\in {\mathscr {B}}^{\rho }(E)$, $0\le s<t$, $F\in \mathcal {F}_{s}^{0}$ and probability measure $\nu \in \mathcal {M}^{\rho }(E)$. By $\mathbb {E}_{\nu }\left[ f(\lambda _{t})\right] <\infty$ it is enough to check

$$\begin{aligned} \mathbb {E}_{\nu }\left[ f(\lambda _{t})\cdot 1_{G}\right] =\mathbb {E}_{\nu }\left[ P\left( t-s\right) f(\lambda _{s})\cdot 1_{G}\right] \end{aligned}$$

for all $G\in \mathcal {G}$ of an intersection stable generator $\mathcal {G}\subset \mathcal {F}_{s}^{0}$. The set

$$\begin{aligned} \left\{ \underset{j\in J}{\bigcap }\left( \lambda _{j}\right) ^{-1}\left( O_{j}\right) :\,J\subset \left[ 0,s \right] \,\text { finite, }O_{j}\subset E\text { open for all}\,j\in J\right\} \end{aligned}$$

is such an intersection stable generator. We fix $k\in \mathbb {N}$ and $0\le r_{1}\le r_{2}\le \cdots \le r_{k}\le s$ and a set $J:=\left\{ r_{1},r_{2}, \ldots ,r_{k},s,t\right\} .$ For $O_{r_{i}}\in E$ open, we have by definition

$$\begin{aligned}&\mathbb {E}_{\nu }\left[ f(\lambda _{t})\cdot 1_{O_{r_{1}}}\left( \lambda _{r_{1}}\right) \cdot ...\cdot 1_{O_{r_{k}}}\left( \lambda _{r_{k}}\right) \right] \\&\quad =\int _{E^{J}}\left( f(x_t)\cdot 1_{O_{r_{1}}}\left( x_{1}\right) \cdot ...\cdot 1_{O_{r_{k}}}\left( x_{k}\right) \cdot 1_{E}(x_{s})\right) p_{\nu }^{J}(dx_{1},...,dx_{k},dx_s,dx_t) \end{aligned}$$

and

$$\begin{aligned}&\mathbb {E}_{\nu }\left[ P\left( t-s\right) f(\lambda _{s})\cdot 1_{O_{r_{1}}}\left( \lambda _{r_{1}}\right) \cdot ...\cdot 1_{O_{r_{k}}}\left( \lambda _{r_{k}}\right) \right] \\&\quad =\int _{E^{J\setminus \left\{ t\right\} }}\left( P\left( t-s\right) f(x_s)\cdot 1_{O_{r_{1}}}\left( x_{1}\right) \cdot ...\cdot 1_{O_{r_{k}}}\left( x_{k}\right) \right) p_{\nu }^{J \setminus \left\{ t\right\} }(dx_{1}, \ldots ,dx_{k},dx_s). \end{aligned}$$

By invoking again Proposition A.4, it is therefore enough to show that the right-hand sides of the above equations coincide on $C_{b}(E)$. By Corollary A.8, there exist sequences of maps $\left( b_{l}^{i}\right) _{l\in \mathbb {N},i\in \left\{ 1, \ldots ,k\right\} }$ where $b_{l}^{i}\in C_{b}\left( E\right)$ for any $l\in \mathbb {N}$ and $i\in \left\{ 1, \ldots ,k\right\}$ such that $\underset{i\in \left\{ 1, \ldots ,k\right\} }{\prod }b_{l}^{i}$ tends to $1_{O_{r_{1}}\times \cdots \times O_{r_{k}}\times E_{s}\times E_{t}}$ as $l \rightarrow \infty$ $p_{\nu }^{J}$-almost surely and $\underset{i\in \left\{ 1, \ldots ,k\right\} }{\prod }b_{l}^{i}$ to $1_{O_{r_{i}}\times \cdots \times O_{r_{k}}\times E_{s}}$, $p_{\nu }^{J{\setminus }\left\{ t\right\} }$-almost surely. For $f\in C_{b}(E_{s})$ by the assumption $P\left( t-s\right) 1=1$, the map $P\left( t-s\right) f$ remains bounded and

$$\begin{aligned}&\int _{E^{J}}\left( f(x_{t})\cdot 1_{O_{x_{1}}}\left( x_{1}\right) \cdot ...\cdot 1_{O_{x_{k}}}\left( x_{k}\right) \right) p_{\nu }^{J}(dx_{1},...,dx_{k},dx_{s},dx_{t})\\&\quad = \underset{l\rightarrow \infty }{\lim }\int _{E^{J}}\left( f(x_t)\cdot b_{l}^{1}\left( x_{1}\right) \cdot ...\cdot b_{l}^{k}\left( x_{k}\right) \right) p_{\nu }^{J}(dx_{1},...,dx_{k},dx_{s},dx_{t})\\&\quad = \underset{l\rightarrow \infty }{\lim }\, \underset{R\rightarrow \infty }{\lim }\int _{E^{J}}1_{\left\{ \rho _{r_{1}}(x_1)<R\right\} }\cdot ...\cdot 1_{\left\{ \rho _{r_{k}}(x_k)<R\right\} }\cdot ...\\&\qquad \cdot \left( f(x_t)\cdot b_{l}^{1}\left( x_{1}\right) \cdot ...\cdot b_{l}^{k}\left( x_{k}\right) \right) \mu _{\nu }^{R,J}(dx_{1},...,dx_{k},dx_{s},dx_{t})\\&\quad = \underset{l\rightarrow \infty }{\lim }\,\underset{R\rightarrow \infty }{\lim }\int _{E^{J\setminus \left\{ x_t\right\} }}1_{\left\{ \rho _{r_{1}}(x_1)<R\right\} }\cdot ...\cdot 1_{\left\{ \rho _{r_{k}}(x_k)<R\right\} }\cdot ...\\&\qquad \cdot \left( P\left( t-s\right) f(x_s)\cdot b_{l}^{1}\left( x_{1}\right) \cdot ...\cdot b_{l}^{k}\left( x_{k}\right) \right) \mu _{\nu }^{R,J\setminus \left\{ t\right\} }(dx_{1},...,dx_{k},dx_{s})\\&\quad = \underset{l\rightarrow \infty }{\lim }\int _{E^{J\setminus \left\{ t\right\} }}\left( P\left( t-s\right) f(x_s)\cdot b_{l}^{1}\left( x_{1}\right) \cdot ...\cdot b_{l}^{k}\left( x_{k}\right) \right) p_{\nu }^{J\setminus \left\{ t\right\} }(dx_{1},...,dx_{k},dx_{s})\\&\quad = \int _{E^{J\setminus \left\{ t\right\} }}\left( P\left( t-s\right) f(x_s)\cdot 1_{O_{r_{1}}}\left( x_{1}\right) \cdot ...\cdot 1_{O_{x_{k}}}\left( x_{k}\right) \right) p_{\nu }^{J\setminus \left\{ t\right\} }(dx_{1},...,dx_{k},dx_{s}). \end{aligned}$$

This shows Eq. (3.3).

*Step 3:* We now show that for the right continuous version of the filtration $\left( \mathcal {F}_{t}\right) _{t\in \mathbb {R}_{+}}:=\left( \mathcal {F}_{t+}^{0}\right) _{t\in \mathbb {R}_{+}}$ the equation

$$\begin{aligned} \mathbb {E}_{\nu }\left[ \left. f(\lambda _{t})\right| \mathcal {F}_{s}\right] =P\left( t-s\right) f(\lambda _{s}) \end{aligned}$$

holds $\mathbb {P}_{\nu }$- almost surely for $f\in {\mathscr {B}}^{\rho }(E)$, $t\ge s\ge 0$ and any probability measure $\nu \in \mathcal {M}^{\rho }(E)$. We fix $f\in {\mathscr {B}}^{\rho }(E)$. It is a standard regularity result that

3.6 $$\begin{aligned} \mathbb {E}_{\nu }\left[ \left. f(\lambda _{t})\right| \mathcal {F}_{s}\right] =\underset{r\searrow s}{\lim }\,\mathbb {E}_{\nu }\left[ \left. f(\lambda _{t})\right| \mathcal {F}_{r}^{0}\right] \end{aligned}$$

holds true $\mathbb {P}_{\nu }$- almost surely for any $t\ge s\ge 0$. Thus, it is sufficient to show

$$\begin{aligned} P\left( t-s\right) f(\lambda _{s})=\underset{r\searrow s}{\lim }\,P\left( t-r\right) f(\lambda _{r}) \end{aligned}$$

$\mathbb {P}_{\nu }$- almost surely for any $t\ge s\ge 0$.

We fix $x_{0}\in E$ and first show the special case of $\nu =\delta _{x_0}$ and $s=0$. In this case, since the left-hand side is deterministic, it is sufficient to show convergence in law, i.e., 

3.7 $$\begin{aligned} \underset{r\searrow 0}{\lim }\,\mathbb {E}_{x_{0}}\left[ h\left( P\left( t-r\right) f(\lambda _{r})\right) \right] =h\left( P\left( t\right) f(x_{0})\right) . \end{aligned}$$

for any $h\in C_{b}(\mathbb {R})$. The maps $r \rightarrow P\left( t-r\right) f$ and $P\left( t-r\right) f \rightarrow h\circ \left( P\left( t-r\right) f\right)$ are continuous by Theorem 2.8 and Lemma A.10, respectively. Hence, by strong continuity of $\left( P(t)\right) _{t\in \mathbb {R}_{+}}$, Lemma I.5.2 in [21] yields

$$\begin{aligned}&\underset{r\searrow 0}{\lim }\,\left( \mathbb {E}_{x_{0}}\left[ h\left( P\left( t-r\right) f(\lambda _{r})\right) \right] -\left( h\circ P\left( t\right) f\right) (x_{0})\right) \\&\quad = \underset{r\searrow 0}{\lim }\,\left( P\left( r\right) \left( h\circ P\left( t-r\right) f\right) (x_{0})-\left( h\circ P\left( t\right) f\right) (x_{0})\right) \\&\quad = P\left( 0\right) \left( h\circ P\left( t\right) f\right) (x_{0})-\left( h\circ P\left( t\right) f\right) (x_{0})\\&\quad = 0. \end{aligned}$$

Therefore, Eq. (3.7) holds and $P\left( t\right) f(\lambda _{0})=\underset{r\searrow 0}{\lim }\,P\left( t-r\right) f(\lambda _{r})$ in $\mathbb {P}_{x_{0}}$- probability for $t\ge 0$. We still need to show the equation for arbitrary $t\ge s\ge 0$, i.e.,

$$\begin{aligned} P\left( t-s\right) f(\lambda _{s})=\underset{r\searrow s}{\lim }\,P\left( t-r\right) f(\lambda _{r}) \end{aligned}$$

$\mathbb {P}_{x_{0}}$-almost surely. By definition of $\mathscr {B}^{\rho ^{\otimes 2}}\left( E\times E\right)$, there exists $\left( f_{n}\right) _{n\in \mathbb {N}}\subset C_{b}\left( E \times E \right)$ such that $f_{n}\left( x,y\right) \rightarrow P\left( t-s\right) f\left( x\right) -P\left( t-r\right) f\left( y\right)$ for any $\left( x,y\right) \in E \times E$. Then by dominated convergence, we have for $\varepsilon >0$,

$$\begin{aligned} \underset{r\searrow s}{\lim }\,\mathbb {E}_{x_{0}}\left[ 1_{\left| P\left( t-s\right) f(\lambda _{0})-P\left( t-r\right) f(\lambda _{r-s})\right|>\varepsilon }\circ \theta _{s}\right] =\, \underset{r\searrow s}{\lim }\,\underset{n\rightarrow \infty }{\lim }\,\mathbb {E}_{x_{0}}\left[ 1_{\left| f_{n}(\lambda _{0},\lambda _{r-s})\right| >\varepsilon }\circ \theta _{s}\right] . \end{aligned}$$

The set $O_{n}:=\left| f_{n}(\lambda _{0},\lambda _{r-s})\right| >\varepsilon$ is open; hence, by Corollary A.8 there is a sequence $\left( h_{n,m}\right) _{m\in \mathbb {N}}\subset C_{b}\left( E\times E\right)$ such that $\mathbb {P}_{x_{0}}$-almost surely $1_{O_{n}} =\underset{m\rightarrow \infty }{\lim }h_{n,m},$ and $0\le h_{n,m}\le 1_{O_{n}}.$ By Lemma A.9, we can approximate $h_{n,m}$ by cylinder functions, and by Proposition 2.11 and the Markov property, we obtain

$$\begin{aligned}&\underset{r\searrow s}{\lim }\,\mathbb {E}_{x_{0}}\left[ 1_{\left| P\left( t-s\right) f(\lambda _{0})-P\left( t-r\right) f(\lambda _{r-s})\right|>\varepsilon }\circ \theta _{s}\right] \\&\quad = \, \underset{r\searrow s}{\lim }\,\underset{n\rightarrow \infty }{\lim }\,\underset{m\rightarrow \infty }{\lim }\,\mathbb {E}_{x_{0}}\left[ h_{n,m}\circ \theta _{s}\right] \\&\quad =\, \underset{r\searrow s}{\lim }\,\underset{n\rightarrow \infty }{\lim }\,\underset{m\rightarrow \infty }{\lim }\,\mathbb {E}_{x_{0}}\left[ \mathbb {E}_{\lambda _{s}}\left[ h_{n,m}\right] \right] \\&\quad \le \, \underset{r\searrow s}{\lim }\,\underset{n\rightarrow \infty }{\lim }\,\mathbb {E}_{x_{0}}\left[ \mathbb {E}_{\lambda _{s}}\left[ 1_{O_{n}}\right] \right] \\&\quad =\, \underset{r\searrow s}{\lim }\,\mathbb {E}_{x_{0}}\left[ \mathbb {E}_{\lambda _{s}}\left[ 1_{\left| P\left( t-s\right) f(\lambda _{0})-P\left( t-r\right) f(\lambda _{r-s})\right| >\varepsilon }\right] \right] = 0. \end{aligned}$$

This yields $P\left( t-s\right) f(\lambda _{s})=\underset{r\searrow s}{\lim }\,P\left( t-r\right) f(\lambda _{r})$ $\mathbb {P}_{x_{0}}$- almost surely, and thus,

$$\begin{aligned} \mathbb {E}_{x_{0}}\left[ \left. f(\lambda _{t})\right| \mathcal {F}_{s}\right] =P\left( t-s\right) f(\lambda _{s}). \end{aligned}$$

Last, we show the general case for any probability measure $\nu \in \mathcal {M}^{\rho }(E)$. By definition of the Baire $\sigma$-algebra, the map

$$\begin{aligned} (x_s,x_r)\rightarrow 1_{\left| P\left( t-s\right) f(x_{s})-P\left( t-r\right) f(x_{r})\right| >\varepsilon } \end{aligned}$$

is measurable with respect to $\mathcal {B}_{0}(E)\times \mathcal {B}_{0}(E)$ . Then by the definition of the probability measure $\mathbb {P}_{\nu }$ and dominated convergence

$$\begin{aligned}&\underset{r\searrow s}{\lim }\,\mathbb {E}_{\nu }\left[ 1_{\left| P\left( t-s\right) f(\lambda _{s})-P\left( t-r\right) f(\lambda _{r})\right|>\varepsilon }\right] \\  &\quad =\int _{E}\left( \underset{r\searrow s}{\lim }\,\mathbb {E}_{x_{0}}\left[ 1_{\left| P\left( t -s\right) f(\lambda _{s})-P\left( t-r\right) f(\lambda _{r})\right| >\varepsilon }\right] \right) d\nu (x_{0}) =0, \end{aligned}$$

and thus,

$$\begin{aligned} P\left( t-s\right) f(\lambda _{s})=\underset{r\searrow 0}{\lim }\,P\left( t-r\right) f(\lambda _{r}) \end{aligned}$$

$$ \mathbb {P}_{\nu }$$-almost surely. $$\square $$

We next state results regarding regularity of the paths of generalized Feller processes and close a gap in [18]. The conditions to get càdlàg versions are actually quite subtle. We refer in this respect to the recent paper [5] where the problem of nonexistence of càdlàg Markov processes associated with classical strong Feller semigroups is addressed. By Theorem 2.8, a generalized Feller semigroup is strongly continuous, so we can define its infinitesimal generator *A*, and for $\beta$ in its resolvent set, we write for the resolvent $R(\beta ,A):=(\beta -A)^{-1}$. Moreover, recall from Remark 2.10 that for strongly continuous semigroups $(P(t))_{t \in \mathbb {R}_+}$ one can always find constants $M\ge 1$ and $\omega \in \mathbb {R}$ such that

3.8 $$\begin{aligned} \left\| P(t)\right\| \le Me^{\omega t}, \quad \text { for all } t\in \mathbb {R}_{+}. \end{aligned}$$

### Theorem 3.4

Let $\left( P(t)\right) _{t\in \mathbb {R}_{+}}$ be a generalized Feller semigroup on ${\mathscr {B}}^{\rho }(E)$ such that for any $t\in \mathbb {R}_{+}$ we have $P(t)1=1$. Let *A* be the generator of $\left( P(t)\right) _{t\in \mathbb {R}_{+}}$. Let $\left( \lambda _{t}\right) _{t\in \mathbb {R}_{+}}$ be the generalized Feller process from Theorem 3.3 on the measurable space $(E^{\mathbb {R}_{+}},\mathcal {B}(E)^{\mathbb {R}_{+}})$ with any initial distribution $\nu \in \mathcal {M}^{\rho }(E)$, and filtration $\left( \mathcal {F}_{t}\right) _{t\in \mathbb {R}_{+}}$, being its right continuous version of the natural filtration as in Theorem 3.3, additionally *augmented* by sets from $\mathcal {F}_\infty$, which are $\mathbb {P}_{\nu }$-nullsets for all $\nu \in \mathcal {M}^{\rho }(E)$. Then the following assertions hold true:

(i) Let $\left( f_{n}\right) _{n\in \mathbb {N}}\subset {\mathscr {B}}^{\rho }(E)$ be a fixed countable family and let $\beta >\omega$ with $\beta \in \mathbb {N}$ be elements of the resolvent set. Then, for the family of stochastic processes $\left( Z_{t}^{\beta ,n}\right) _{t\in \mathbb {R}_{+}}$ defined as
  $$\begin{aligned} Z_{t}^{\beta ,n}:=\beta R(\beta ,A)f_{n}(\lambda _{t}) \end{aligned}$$
  there exists a family of $\left( \mathcal {F}_{t}\right) _{t\in \mathbb {R}_{+}}$-adapted stochastic processes $\left( \bar{Z}_{t}^{\beta ,n}\right) _{t\in \mathbb {R}_{+}}$ with càdlàg paths, such that simultaneously for all $\nu \in \mathcal {M}^{\rho }(E)$
  $$\begin{aligned} { \mathbb {P_{\nu }}[Z_{t}^{\beta ,n}=\bar{Z}_{t}^{\beta ,n}]=1 \quad } \end{aligned}$$
  for all $t \in \mathbb {R}_+$ and $n,\beta \in \mathbb {N}$ with $\beta >\omega$.
(ii) Let $\rho$ be Baire-measurable. If the constant *M* in (3.8) can be chosen to be 1, then
  $$\begin{aligned} \left( \exp \left( -\omega t\right) \rho (\lambda _{t})\right) _{t\in \mathbb {R}_{+}} \end{aligned}$$
  with $\omega$ as in (3.8) is a $\mathbb {P_{\nu }}$-supermartingale for any $\nu \in \mathcal {M}^{\rho }(E)$. If $t\rightarrow P(t)\rho (x)$ is right continuous for any $x\in E$, then the supermartingale has an $(\mathcal {F}_t)_{t \in \mathbb {R}_+}$ -adapted version with càdlàg paths, simultaneously for all $\nu \in \mathcal {M}^{\rho }(E)$.
(iii) If additionally to the assumptions in (i) and (ii) there exists a countable family $\left( f_{n}\right) _{n\in \mathbb {N}}\subset {\mathscr {B}}^{\rho }(E)$ whose span is dense in ${\mathscr {B}}^{\rho }(E)$, then $\left( \lambda _{t}\right) _{t\in \mathbb {R}_{+}}$ has an $(\mathcal {F}_t)_{t \in \mathbb {R}_+}$ - adapted version with càdlàg paths, again simultaneously for all $\nu \in \mathcal {M}^{\rho }(E)$.

### Proof

(i) Recall that *A* denotes the infinitesimal generator of the generalized Feller semigroup $(P(t))_{t \in \mathbb {R}_+}$. By the integral representation of the resolvent, we have for $\beta >\omega$ with $\omega$ as in (3.8) and for all $f\in {\mathscr {B}}^{\rho }(E)$

$$\begin{aligned} \left( \beta -A\right) ^{-1}f:=R(\beta ,A)f=\int _{0}^{\infty }e^{-\beta s}P(s)f\,ds. \end{aligned}$$

In order to apply Theorem II.65.1 and Theorem II.66.2 in [32] (or Theorem II.2.9 in [30]), we first need to find a suitable supermartingale with some $L^1$-path regularity. For this purpose, we fix $0\le f\in {\mathscr {B}}^{\rho }(E)$, and $\beta >\omega$ and we define the stochastic process $\left( Y_{t}^{\beta ,f}\right) _{t\in \mathbb {R}_{+}}$ by

$$\begin{aligned} Y_{t}^{\beta ,f}:=\exp \left( -\beta t\right) R(\beta ,A)f(\lambda _{t}). \end{aligned}$$

We show that $\left( Y_{t}^{\beta ,f}\right) _{t\in \mathbb {R}_{+}}$ is a supermartingale with respect to $\left( \mathcal {F}_{t}\right) _{t\in \mathbb {R}_{+}}$. Let $0\le s\le t$ and calculate

$$\begin{aligned} \mathbb {E}_{\nu }\left[ \left. Y_{t}^{\beta ,f}\right| \mathcal {F}_{s}\right]&=\exp \left( -\beta t\right) \mathbb {E}_{\nu }\left[ \left. \int _{0}^{\infty }\exp \left( -\beta u\right) P(u)f(\lambda _{t})du\right| \mathcal {F}_{s}\right] . \end{aligned}$$

By definition of the Riemann integral that takes values in the Banach space ${\mathscr {B}}^{\rho }(E)$, positivity of the semigroup $\left( P(t)\right) _{t\in \mathbb {R}_{+}}$, and monotone convergence for conditional expectations and thanks to $\left( \lambda _{t}\right) _{t\in \mathbb {R}_{+}}$ being a generalized Feller process, we obtain

$$\begin{aligned}&\mathbb {E}_{\nu }\left[ \left. \int _{0}^{\infty }\exp \left( -\beta u\right) P(u)f(\lambda _{t})du\right| \mathcal {F}_{s}\right] \\&\quad = \mathbb {E}_{\nu }\left[ \left. \underset{n\rightarrow \infty }{\lim }\,\underset{m\rightarrow \infty }{\lim }\left( n/m\right) \cdot {\sum _{i=0}^{m-1}}\exp \left( -\beta in/m\right) P(in/m)f(\lambda _{t})\right| \mathcal {F}_{s}\right] \\&\quad = \underset{n\rightarrow \infty }{\lim }\,\underset{m\rightarrow \infty }{\lim }\left( n/m\right) \cdot {\sum _{i=0}^{m-1}}\exp \left( -\beta in/m\right) \mathbb {E}_{\nu }\left[ \left. P(in/m)f(\lambda _{t})\right| \mathcal {F}_{s}\right] \\&\quad = \underset{n\rightarrow \infty }{\lim }\,\underset{m\rightarrow \infty }{\lim }\left( n/m\right) \cdot {\sum _{i=0}^{m-1}}\exp \left( -\beta in/m\right) P(in/m+t-s)f(\lambda _{s})\\&\quad = \exp \left( \beta \left( t-s\right) \right) \underset{n\rightarrow \infty }{\lim }\,\underset{m\rightarrow \infty }{\lim }\left( n/m\right) \\  &\qquad \cdot {\sum _{i=0}^{m-1}}\exp \left( -\beta \left( in/m+t-s\right) \right) P(in/m+t-s)f(\lambda _{s}). \end{aligned}$$

Thus,

$$\begin{aligned} \mathbb {E}_{\nu }\left[ \left. Y_{t}^{\beta ,f}\right| \mathcal {F}_{s}\right]&=\exp \left( -\beta s\right) \int _{t-s}^{\infty }\left( \exp \left( -\beta r\right) P(r)f(\lambda _{s})\right) dr\\&\le Y_{s}^{\beta ,f}, \end{aligned}$$

where the last inequality follows again from $f\ge 0$ and positivity of the semigroup $\left( P(t)\right) _{t\in \mathbb {R}_{+}}$. Furthermore,

$$\begin{aligned} \mathbb {E}_{\nu }\left[ Y_{t}^{\beta ,f}\right] =\exp \left( -\beta s\right) \int _{t-s}^{\infty }\left( \exp \left( -\beta r\right) \mathbb {E}_{\nu }\left[ P(r)f(\lambda _{s})\right] \right) dr, \end{aligned}$$

which implies continuity of $t\rightarrow \mathbb {E}_{\nu }\left[ Y_{t}^{\beta ,f}\right] .$

We can now apply Theorem II.65.1 in [32] (or Theorem II.2.9 in [30]) based on upwards crossing inequalities to obtain a set $\Omega _{\beta ,f}^{'}\in \mathcal {B}(E)^{\mathbb {R}_{+}}$ with $\mathbb {P}_{\nu }\left( \Omega _{\beta ,f}^{'}\right) =1$ for every $\nu \in \mathcal {M}^{\rho }(E)$ on which

$$\begin{aligned} \left( Y_{s}^{\beta ,f} \right) _{s \in \mathbb {Q}_+} \end{aligned}$$

has a càdlàg extension to $\mathbb {R}_+$, which will be denoted by $\left( \bar{Y}_{t}^{\beta ,f}\right) _{t\in \mathbb {R}_{+}}$ (compare also the discussion in [32] on page 245). Outside of $\Omega _{\beta ,f}^{'}$, we set $\bar{Y}^{\beta ,f}$ equal to 0. Moreover, by Theorem II.66.2 in [32] and the fact that $\mathcal {F}_0$ contains all sets which are $\mathbb {P}_{\nu }$-nullsets for all $\nu$ simultaneously, the process $\left( \bar{Y}_{t}^{\beta ,f}\right) _{t\in \mathbb {R}_{+}}$ is adapted to $(\mathcal {F}_t)_{t\in \mathbb {R}_{+}}$ and we have

$$\begin{aligned} \mathbb {P}_{\nu }[Y_{t}^{\beta ,f}= \bar{Y}_{t}^{\beta ,f}]=1, \quad \text { for all } t \in \mathbb {R}_+ \text { and } \nu \in \mathcal {M}^{\rho }(E), \end{aligned}$$

implying that $(\bar{Y}_{t}^{\beta ,f})_{t\in \mathbb {R}_{+}}$ is a a version of $\left( Y_{t}^{\beta ,f}\right) _{t\in \mathbb {R}_{+}}$ for any $\mathbb {P}_{\nu }$.

Therefore, for any $g\in {\mathscr {B}}^{\rho }(E)$, clearly $g^{+},g^{-}\in {\mathscr {B}}^{\rho }(E)$, and $g^{+},g^{-}\ge 0$ and the process $\left( Y_{t}^{\beta ,g}\right) _{t\in \mathbb {R}_{+}}$ has a version with càdlàg paths. The same holds true for any $n\in \mathbb {N}$ and $\beta >\omega$ for the process $\left( Z_{t}^{\beta ,n}\right) _{t\in \mathbb {R}_{+}}$. The statement of part (i) of the theorem then follows directly from the fact that the countable union of null sets is a null set.

(ii) Since *M* is supposed to be 1, we have $P(t)\rho \le \exp \left( \omega t\right) \rho$ for some $\omega \in \mathbb {R}$. Moreover, as $\rho$ is Baire-measurable, Remark 3.2 implies that for all $t\ge s \ge 0$ and for any probability measure $\nu \in \mathcal {M}^{\rho }(E)$

$$\begin{aligned} \mathbb {E}_{\nu }\left[ \left. \exp \left( -\omega t\right) \rho (\lambda _{t})\right| \mathcal {F}_{s}\right]&=\exp \left( -\omega t\right) P(t-s)\rho (\lambda _{s})\\&\le \exp \left( -\omega s\right) \rho (\lambda _{s}), \end{aligned}$$

whence $\left( \exp \left( -\omega t\right) \rho (\lambda _{t})\right) _{t\in \mathbb {R}_{+}}$ is a nonnegative $\mathbb {P}_{\nu }$-supermartingale for any $\nu \in \mathcal {M}^{\rho }(E)$. Again, by Theorem II.65.1 in [32], the stochastic process $\left( \rho (\lambda _{s}) \right) _{s\in \mathbb {Q}_{+}}$ has a càdlàg extension $\left( \overline{\rho (\lambda _{s})}\right) _{s\in \mathbb {R}_{+}}$ to $\mathbb {R}_+$ on a measurable set of probability 1 with respect to any $\mathbb {P}_{\nu }$. By setting $\overline{\rho (\lambda )}$ equal to 0 outside this set, Theorem II.66.2 in [32] yields again that $\left( \overline{\rho (\lambda _{s})}\right) _{s\in \mathbb {R}_{+}}$ is a $\mathbb {P}_{\nu }$-supermartingale for all $\nu \in \mathcal {M}^{\rho }(E)$. Note again that $\mathcal {F}_0$ is constructed appropriately for this purpose, since it is made to contain the sets from $\mathcal {F}_\infty$, which are $\mathbb {P}_\nu$-nullsets for all $\nu$. Moreover, since $t\rightarrow P(t)\rho (x)$ is right continuous for any $x\in E$ and thus, by dominated convergence, also $t \rightarrow \mathbb {E}_{\nu }[\rho (\lambda _t)]$ for any $\nu$, we obtain by Theorem II.66.2 in [32] that $\left( \overline{\rho (\lambda _{s})}\right) _{s\in \mathbb {R}_{+}}$ is a version of $\left( \rho (\lambda _{t})\right) _{t\in \mathbb {R}_{+}}$ for every $\mathbb {P}_{\nu }$.

Statement (iii) is also a bit more involved than for standard Feller theory, see, e.g., Section III.7 in [32] for comparison, but it is still a consequence of (i) and (ii).

Choose $\beta > \omega$ sufficiently large: $(f_n)_{n \in \mathbb {N}}$ spans a dense subset by assumption, so $(R(\beta ,A)f_n)_{n \in \mathbb {N}}$ spans a dense subset, too, since the range of the resolvent for sufficiently large $\beta$ is dense (which just says that the domain of the generator is dense). Whence the map $F: x \ni E \mapsto (\beta R(A,\beta ) f_n(x))_{n \in \mathbb {N}} \in \mathbb {R}^{\mathbb {N}}$, which is continuous on sets of the form $\{ \rho \le R \}$, is actually injective. Therefore, the restrictions of *F* on the compact set $\{ \rho \le R \}$ induce homeomorphisms onto their range for every $R > 0$. Similarly, as above, we consider now a measurable subset $\Omega ' \subset \mathcal {F}_\infty$ with $\mathbb {P}_{\nu }(\Omega ')=1$ for any $\nu$, where $(F(\lambda _s))_{s \in \mathbb {Q}_+}$ and $(\rho (\lambda _s))_{s \in \mathbb {Q}_+}$ have a càdlàg extension to $\mathbb {R}_+$, as proved in (i) and (ii). Outside of $\Omega '$, we set all involved processes to 0.

Since $\sup _{s \le t, s \in \mathbb {Q}_+} \rho (\lambda _s)$ is finite point by point as a consequence of the existence of a càdlàg extension for every $t \ge 0$ on $\Omega '$, we know that the coordinate process on rational times less than *t* lies point by point in a compact of *E* on $\Omega '$.

Fix now $t > 0$ and $\omega ' \in \Omega '$: then $\left( \lambda _r(\omega ') \right) _{r \le t, \, r \in \mathbb {Q}_+}$ takes values in $\{ \rho \le n(\omega ') \}$ for some $n(\omega ')$ large enough. Since *F* is a homeomorphism from compacts of the form $\{\rho \le R \}$, for any $R >0$, onto its range, and since $r \mapsto F(\lambda _r(\omega '))$ on $r \in \mathbb {Q}_+ \cap [0,t]$ has a càdlàg extension on [0, *t*], then $\left( \lambda _r(\omega ') \right) _{r \le t, \, r \in \mathbb {Q}_+}$ has an extension to [0, *t*] by inversion of *F*.

Since $t >0$ and $\omega ' \in \Omega '$ were arbitrary, we can define a càdlàg extension $\bar{\lambda }$ on $\Omega '$. The nullsets needed for the adaptedness of $\bar{\lambda }$ lie in $\mathcal {F}_0$ (as it is augmented by sets in $\mathcal {F}_{\infty }$ which are nullsets for all $\mathbb {P}_{\nu }$). The question remains whether this extension is a version of $\lambda$: This is, however, the case since the extension of $\lambda$ are done on compacts in time [0, *t*] and within compacts of the form $\{ \rho \le R \}$ in *E*, where *F* is a homeomorphism; compare Section III.7, formula (7.12) in [32]. Indeed

$$\begin{aligned} \mathbb {E}_\nu [f_1(\lambda _t)f_2(\overline{\lambda _t})]= &   \lim _{q \searrow t} \mathbb {E}_\nu [f_1(\lambda _t) f_2(\lambda _q)] = \lim _{q \searrow t} \mathbb {E}_\nu [f_1(\lambda _t) P_{q -t} f_2(\lambda _t)]\\= &   \mathbb {E}_\nu [f_1(\lambda _t)f_2(\lambda _t)] \end{aligned}$$

for all $f_1,f_2 \in {\mathscr {B}}^{\rho }(E)$, where $f_1$ is assumed to be globally bounded. By a monotone class argument, we obtain $\mathbb {E}_\nu [f(\lambda _t, \overline{\lambda _t})] = \mathbb {E}_\nu [f(\lambda _t,\lambda _t)]$ for all *f* on $E \times E$ that are bounded and Baire-measurable. From this, we can conclude that

$$\begin{aligned} \mathbb {P}_{\nu }[\lambda _t= \overline{\lambda }_t]=\mathbb {E}_\nu [1_{\{\lambda _t= \overline{\lambda }_t\}}]=\mathbb {E}_\nu [1_{\{\lambda _t= \lambda _t\}}]=1, \quad \text { for all } t \in \mathbb {R}_+ \text { and } \nu \in \mathcal {M}^{\rho }(E), \end{aligned}$$

which proves the claim. $\square$

## Extended Feller processes

We shall here introduce a new class of stochastic process, called *extended Feller processes,* which are in a different way connected to generalized Feller semigroups. As killing shall play a certain role, we recall the cemetery state $\Delta$ and equip $E\cup \left\{ \Delta \right\}$ with a topology such that

$$\begin{aligned} \mathcal {B}(E\cup \left\{ \Delta \right\} )=\sigma \left( \mathcal {B}(E),\left\{ \Delta \right\} \right) . \end{aligned}$$

Consistent with the convention made for the cemetery, we define ${\mathscr {B}}^{\rho }(E\cup \left\{ \triangle \right\} )$ as the space of maps *f* such that $\left. f\right| _{E}\in {\mathscr {B}}^{\rho }(E)$ and $f(\Delta )=0.$ The space $C_{0}\left( E\cup \left\{ \triangle \right\} \right)$ is defined in the same fashion.

### Definition 4.1

Let $\rho$ be measurable with respect to the Baire $\sigma$-algebra $\mathcal {B}_0(E)$ and let $\left( P(t)\right) _{t\in \mathbb {R}_{+}}$ be a generalized Feller semigroup on ${\mathscr {B}}^{\rho }(E)$, let $\nu$ be a probability measure on

$$\begin{aligned} \left( E\cup \left\{ \Delta \right\} ,\mathcal {B}(E\cup \left\{ \Delta \right\} )\right) \end{aligned}$$

and let $\left( \gamma _{t}\right) _{t\in \mathbb {R}_{+}}$ be an adapted stochastic process on the filtered probability space

$$\begin{aligned} \left( \left( E\cup \left\{ \triangle \right\} \right) ^{\mathbb {R}_{+}},\mathcal {B}(E\cup \left\{ \Delta \right\} )^{\mathbb {R}_{+}},\left( \mathcal {F}_{t}\right) _{t\in \mathbb {R}_{+}},\mathbb {P}'_{\nu }\right) . \end{aligned}$$

If for any $t\ge s\ge 0$ and any real-valued map *f* on $E\cup \left\{ \Delta \right\}$ that is bounded and Baire-measurable

4.1 $$\begin{aligned} \mathbb {E}_{\mathbb {P}'_{\nu }}\left[ \left. f(\gamma _{t})\right| \mathcal {F}_{s}\right] =\frac{P\left( t-s\right) \left( f\cdot \rho \right) }{\rho }(\gamma _{s}) \end{aligned}$$

holds true $\mathbb {P}'_{\nu }$-almost surely and

$$\begin{aligned} \mathbb {P}'_{\nu }\circ \gamma _{0}^{-1}=\nu , \end{aligned}$$

then $\left( \gamma _{t}\right) _{t\in \mathbb {R}_{+}}$ is called $extended$ $Feller process$ with respect to $\left( \mathcal {F}_{t}\right) _{t\in \mathbb {R}_{+}}$ and with respect to $\left( P(t)\right) _{t\in \mathbb {R}_{+}}$ with initial distribution $\nu$.

Similarly as above, we make the convention $\mathbb {P}'_{x}:=\mathbb {P}'_{\delta _{x}}$ for any $x\in E$.

The reason for the choice of the name will become clear in Theorem 5.6 which essentially states that when $\rho$ is continuous (and thus *E* locally compact) then the restriction of (4.1) to $C_0(E)$ is a Feller semigroup.

### Remark 4.2

Just like for generalized Feller processes, note that (4.1) is only required to hold for Baire-measurable functions and in general does not hold for any positive Borel-measurable function. However, the latter does hold true on metrizable spaces.

In the following theorem, we obtain existence of extended Feller processes for contractive generalized Feller semigroups.

### Theorem 4.3

Let $\rho$ be measurable with respect to the Baire $\sigma$-algebra $\mathcal {B}_{0}(E)$. Let $\left( P(t)\right) _{t\in \mathbb {R}_{+}}$ be a generalized Feller semigroup on ${\mathscr {B}}^{\rho }(E)$ such that for all $t\in \mathbb {R}_{+}$

$$\begin{aligned} \left\| P(t)\right\| _{L({\mathscr {B}}^{\rho }(E))}\le 1. \end{aligned}$$

Then for any probability measure $\nu$ on

$$\begin{aligned} \left( E\cup \left\{ \Delta \right\} ,\mathcal {B}(E\cup \left\{ \Delta \right\} )\right) \end{aligned}$$

there exists a probability measure $\mathbb {P}'_{\nu }$ on the measurable space

$$\begin{aligned} \left( \left( E\cup \left\{ \triangle \right\} \right) ^{\mathbb {R}_{+}},\mathcal {B}(E\cup \left\{ \Delta \right\} )^{\mathbb {R}_{+}}\right) \end{aligned}$$

such that for the canonical process $\left( \gamma _{t}\right) _{t\in \mathbb {R}_{+}}$ and the natural filtration $\left( \mathcal {F}_{t}^{0}\right) _{t\in \mathbb {R}_{+}}$ for any $t\ge s\ge 0$ and any real-valued map *f* on $E\cup \left\{ \Delta \right\}$ that is bounded and Baire-measurable

4.2 $$\begin{aligned} \mathbb {E}_{\mathbb {P}'_{\nu }}\left[ \left. f(\gamma _{t})\right| \mathcal {F}_{s}^{0}\right] =\frac{P\left( t-s\right) \left( f\cdot \rho \right) }{\rho }(\gamma _{s}) \end{aligned}$$

holds true $\mathbb {P}'_{\nu }$-almost surely and

$$\begin{aligned} \mathbb {P}'_{\nu }\circ \gamma _{\text {0}}^{-1}=\nu . \end{aligned}$$

If *f* is such that $f\cdot \rho \in {\mathscr {B}}^{\rho }(E\cup \left\{ \triangle \right\} )$ then Eq. (4.2) holds true also for the complete right continuous extension of the filtration.

### Remark 4.4

Note that due to the assumption $\left\| P(t)\right\| _{L({\mathscr {B}}^{\rho }(E))}\le 1$, $\rho$ is by Remark 2.10 0-superaveraging in the terminology of [11]. Moreover, the extended Feller process $\gamma$ constructed in Theorem 4.3 is closely related to Doob’s h-transform. Indeed, on locally compact metric spaces the semigroup of Doob’s h-transform is defined exactly as in (4.2) with $\rho$ taking the role of *h*. The only difference is that we here do not require $\lim _{t \rightarrow 0} P_t \rho (x) = \rho (x)$, which together with 0-superaveraging is the definition of 0-excessive functions that are used to define Doob’s *h*-transform (see, e.g., Chapter 11 in [11]).

### Proof

We fix some probability measure $\nu$ on $\left( E\cup \left\{ \Delta \right\} ,\mathcal {B}(E\cup \left\{ \Delta \right\} )\right)$ and divide the proof into three steps similarly as in the proof of Theorem 3.3. We also apply the notation introduced in the beginning of Sect. 3.

In the first step, we define a family of sub-probability measures on the space

$$\begin{aligned} \left( \left( E\cup \left\{ \Delta \right\} \right) ^{J},\left( \mathcal {B}\left( E\cup \left\{ \Delta \right\} \right) \right) ^{J}\right) _{J\subset \mathbb {R}_{+},\text { finite}}. \end{aligned}$$

and show in the second step that this family is projective. In the third step, we can then apply the generalized Kolmogorov extension theorem (Theorem 15.26 in [3]) to obtain the statement of the theorem.

*Step 1:* On $\left( \left( E\cup \left\{ \Delta \right\} \right) ^{J},\left( \mathcal {B}\left( E\cup \left\{ \Delta \right\} \right) \right) ^{J}\right) _{J\subset \mathbb {R}_{+},\text { finite}},$ define a family of probability measures via

$$\begin{aligned} \left( \left( q_{\nu }^{J}\right) \right) _{J\subset \mathbb {R}_{+},\text { finite}}. \end{aligned}$$

We fix some $s\in \mathbb {R}_{+}$, and by Lemma 2.9, we find $p(s)(x,\cdot )\in \mathcal {M}^{\rho }(E)$ such that

$$\begin{aligned} P(s)f(x)=\int _{E}f(y)p(s)(x,dy)\,\text {for all }x\in E. \end{aligned}$$

By Remark 2.10, we have $P(s)\rho (x) \le \rho (x)$ for all $x\in E$ and we define the measures $q(s)(x,\cdot )$ via

$$\begin{aligned} q(s)(x,A):=\int _{E}1_{A}(y)\frac{\rho (y)}{\rho (x)}p(s)(x,dy)\text { for }A\in \mathcal {B}(E). \end{aligned}$$

Consequently, $q(s)(x,E)\le 1$. For any $s\in \mathbb {R}_{+}$ for any $x\in E$, we define the measures $\tilde{q}(s)(x,\cdot )$ on $E\cup \left\{ \Delta \right\}$ by

$$\begin{aligned} \left. \tilde{q}(s)(x,\cdot )\right| _{\mathcal {B}(E)}:=q(s)(x,\cdot ) \end{aligned}$$

and

$$\begin{aligned} \tilde{q}(s)(x,\left\{ \Delta \right\} ):=1-q(s)(x,E). \end{aligned}$$

Furthermore,

$$\begin{aligned} \tilde{q}(s)(\Delta ,\left\{ \Delta \right\} ):=1 \end{aligned}$$

for any $s\in \mathbb {R}_{+}$. Thanks to Proposition 2.11, we can define the semigroup $\left( Q(t)\right) _{t\in \mathbb {R}_{+}}$ on the space of bounded Baire-measurable maps by

$$\begin{aligned} Q(t)f(x)=\int _{E}f(y)\tilde{q}(t)(x,dy). \end{aligned}$$

For any finite $J:=\left\{ r_{1},...,r_{n}\right\} \subset \mathbb {R}_{+}$ by Lemma A.9, there is a unique continuous map

$$\begin{aligned} j_{J,\nu }:\,{\mathscr {B}}^{\rho ^{\otimes J}}(\left( E\cup \left\{ \Delta \right\} \right) ^{J})\rightarrow \mathbb {R} \end{aligned}$$

such that

$$\begin{aligned} f_{r_{1}}\cdot ...\cdot f_{r_{n}}&\rightarrow \int _{E}Q(r_{1})\left( \left( \frac{f_{r_{1}}}{\rho _{r_{1}}}\right) \cdot ...\cdot \left( Q(r_{n-1}-r_{n-2})\left( \frac{f_{r_{n-1}}}{\rho _{r_{n-1}}}\right) \cdot \left( Q(r_{n}-r_{n-1})\left( \frac{f_{r_{n}}}{\rho _{r_{n}}}\right) \right) \right) \right) (x_{0})\nu (dx_{0}) \end{aligned}$$

for any $f\in {\mathscr {B}}^{\rho ^{\otimes J}}\left( \left( E\cup \left\{ \Delta \right\} \right) ^{J}\right)$ given by

$$\begin{aligned} f(x_{J}):=f_{r_1}(x_{r_1})\cdot \ldots \cdot f_{r_n}(x_{r_n}). \end{aligned}$$

By Theorem 2.4, there exists a unique finite positive Radon measure

$$\begin{aligned} \mu _{\nu }^{J}\in \mathcal {M}^{\rho ^{\otimes J}}\left( \left( E\cup \left\{ \Delta \right\} \right) ^{J}\right) , \end{aligned}$$

such that for any $f\in {\mathscr {B}}^{\rho ^{\otimes J}}\left( \left( E\cup \left\{ \Delta \right\} \right) ^{J}\right) .$

$$\begin{aligned} j_{J,\nu }(f)=\int _{E^{J}}f(x_{J})\mu _{\nu }^{J}(dx_{J}), \quad \text {and} \quad \int _{E^{J}}\rho ^{\otimes J}(x_{J})\mu _{\nu }^{J}(dx_{J})=1. \end{aligned}$$

We now define the family of probability measures $\left( q_{\nu }^{J}\right) _{J\subset \mathbb {R}_{+},\text { finite}}$ on

$$\begin{aligned} \left( \left( E\cup \left\{ \Delta \right\} \right) ^{J},\mathcal {B}(\left( E\cup \left\{ \Delta \right\} \right) ^{J})\right) _{J\subset \mathbb {R}_{+},\text { finite}} \end{aligned}$$

by

$$\begin{aligned} \mathcal {B}(E^{J})&\rightarrow \left[ 0,1\right] \\ A&\rightarrow \int _{A}\rho ^{\otimes J}(x_{J})\mu _{\nu }^{J}(dx_{J}). \end{aligned}$$

We observe the non-obvious fact that for any finite $J\subset \mathbb {R}_{+}$ the measure $q_{\nu }^{J}$ is a Radon measure (since the space $E^{J}$ is non-necessarily Polish). To see this, fix a finite $\tilde{J}\subset \mathbb {R}_{+}$. Let $A\in \mathcal {B}\left( E^{\tilde{J}}\right)$ and $\varepsilon >0$ be arbitrary. Then by $\underset{R>0}{\bigcup }K_{R}=E$, there exists $R_{\varepsilon }>0$ such that

$$\begin{aligned} q_{\nu }^{\tilde{J}}\left( E^{\tilde{J}}\setminus \left( K_{R_{\varepsilon }}\right) ^{\tilde{J}}\right) <\frac{\varepsilon }{2}. \end{aligned}$$

Since $\mu _{\nu }^{\tilde{J}}$ is a Radon measure, there exists $K\subset A\cap \left( K_{R_{\varepsilon }}\right) ^{\tilde{J}}$ such that

$$\begin{aligned} \mu _{\nu }^{\tilde{J}}\left( A\cap \left( K_{R_{\varepsilon }}\right) ^{\tilde{J}}\setminus K\right) <\frac{\varepsilon }{2R_{\varepsilon }}. \end{aligned}$$

Thus,

$$\begin{aligned} q_{\nu }^{\tilde{J}}\left( A\setminus K\right)&\le q_{\nu }^{\tilde{J}}\left( E^{\tilde{J}}\setminus \left( K_{R_{\varepsilon }}\right) ^{\tilde{J}}\right) +q_{\nu }^{\tilde{J}}\left( A\cap \left( K_{R_{\varepsilon }}\right) ^{\tilde{J}}\setminus K\right) \le \frac{\varepsilon }{2}+\frac{\varepsilon }{2}, \end{aligned}$$

and the probability measure $q_{\nu }^{\tilde{J}}$ is inner regular, hence a Radon measure.

*Step 2:* We now show that the family $\left( q_{\nu }^{J}\right) _{J\subset \mathbb {R}_{+},\text { finite}}$ is projective. To this end, it is sufficient to show for any finite $J:=\left\{ r_{1}, \ldots ,r_{n}\right\} \subset \mathbb {R}_{+}$ and $j\in \left\{ 1, \ldots ,n\right\}$ for any $A_{i}\in \mathcal {B}(E\cup \left\{ \Delta \right\} ){}^{r_{i}}$, $i\in \left\{ 1, \ldots ,n\right\} \setminus \left\{ j\right\}$

$$\begin{aligned} q_{\nu }^{J}(A_{1}\times \cdots \times A_{j-1}\times E_{j}\times A_{j+1}...\times A_{n})=q_{\nu }^{J\setminus \left\{ r_{j}\right\} }(A_{1}\times ...\times A_{j-1}\times A_{j+1}...\times A_{n}). \end{aligned}$$

We observe that by Corollary A.8 indicator functions of open sets can be approximated by continuous bounded maps almost surely with respect to finitely many measures. Hence, any set in the Borel $\sigma$-algebra can be approximated by continuous bounded maps almost surely with respect to finitely many measures. Approximating $A_{1}\times ...\times A_{j-1}\times E_{j}\times A_{j+1}...\times A_{n}$ as the product of *n* indicator function in such a way, projectivity of the family $\left( q_{\nu }^{J}\right) _{J\subset \mathbb {R}_{+},\text { finite}}$ follows by dominated convergence from the definition of the family $\left( \mu _{\nu }^{J}\right) _{J\subset \mathbb {R}_{+},\text { finite}}$ on the cylinder functions.

*Step 3:* As in the proof of Theorem 3.3, one can easily show that

$$\begin{aligned} \mathcal {C}:&=\left\{ C:\,C\text { compact},\,C\subset K_{R}\text { for }R\ge 0\right\} \cup \left\{ C\cup \left\{ \Delta \right\} :\,C\text { compact},\,C\subset K_{R}\text { for }R\ge 0\right\} \end{aligned}$$

is a compact class in $E\cup \left\{ \Delta \right\}$ and that for each $t\in \mathbb {R}_{+}$ and $A\in \mathcal {B}(E\cup \left\{ \Delta \right\} )$

$$\begin{aligned} \left( q_{\nu }^{\left\{ t\right\} }\right) (A)=\sup \left\{ \left( q_{\nu }^{\left\{ t\right\} }\right) (C):\,C\subset A\text {\,and }C\in \mathcal {C}\right\} . \end{aligned}$$

Therefore, we can apply Theorem 15.26 in [3]. This yields a measure $\mathbb {P}'_{\nu }$ on

$$\begin{aligned} \left( \left( E\cup \left\{ \Delta \right\} \right) ^{\mathbb {\mathbb {R}}_{+}},\left( \mathcal {B}(E\cup \left\{ \Delta \right\} )\right) {}^{\mathbb {\mathbb {R}}_{+}}\right) \end{aligned}$$

such that its projections are the family $\left( q_{\nu }^{J}\right) _{J\subset \mathbb {R}_{+},\text { finite}}$. Furthermore,

$$\begin{aligned} \mathbb {P}'_{\nu }\circ \gamma _{\text {0}}^{-1}=\nu , \end{aligned}$$

by the definition of $\mathbb {P}'_{\nu }$ via the functional $j_{\left\{ 0\right\} ,\nu }$.

Equation (4.2) follows from the fact that we can approximate bounded Baire-measurable functions by continuous bounded function and the same reasoning as in the proof of Theorem 3.3.

Finally, for *f* such that $f\cdot \rho \in {\mathscr {B}}^{\rho }(E\cup \left\{ \triangle \right\} )$ the statement concerning the right continuous extension of the filtration follows as in the proof of Theorem 3.3. $\square$

The following corollary extends the above statement from contraction semigroups to quasi-contraction semigroups.

### Corollary 4.5

Let $\rho$ be Baire-measurable and let $\left( P(t)\right) _{t\in \mathbb {R}_{+}}$ be a generalized Feller semigroup on ${\mathscr {B}}^{\rho }(E)$ such that for some $\omega \in \mathbb {R}$ and all $t\in \mathbb {R}_{+}$

$$\begin{aligned} \left\| P(t)\right\| _{L({\mathscr {B}}^{\rho }(E))}\le e^{\omega t}. \end{aligned}$$

Then for any probability measure $\nu$ on $\left( E\cup \left\{ \triangle \right\} ,\mathcal {B}(E\cup \left\{ \Delta \right\} )\right)$, there exists a probability measure $\mathbb {P}'_{\nu }$ on

$$\begin{aligned} \left( \left( E\cup \left\{ \triangle \right\} \right) ^{\mathbb {R}_{+}},\mathcal {B}(E\cup \left\{ \Delta \right\} ){}^{\mathbb {R}_{+}}\right) \end{aligned}$$

such that for the canonical process $\left( \gamma _{t}\right) _{t\in \mathbb {R}_{+}}$ for any $t\ge s\ge 0$ and any real-valued map *f* on $E\cup \left\{ \Delta \right\}$ that is bounded and Baire-measurable

4.3 $$\begin{aligned} \mathbb {E}_{\mathbb {P}'_{\nu }}\left[ \left. f(\gamma _{t})\right| \mathcal {F}_{s}^{0}\right] =\frac{e^{-\omega t}P\left( t-s\right) \left( f\cdot \rho \right) }{\rho }(\gamma _{s}) \end{aligned}$$

holds true $\mathbb {P}'_{\nu }$-almost surely (where $\left( \mathcal {F}_{t}^{0}\right) _{t\in \mathbb {R}_{+}}$ is the natural filtration) and

$$\begin{aligned} \mathbb {P}'_{\nu }\circ \gamma _{\text {0}}^{-1}=\nu . \end{aligned}$$

If *f* is such that $f\cdot \rho \in {\mathscr {B}}^{\rho }(E\cup \left\{ \triangle \right\} )$, then Eq. (4.3) holds true also for the right continuous extension of the filtration.

### Proof

Define the rescaled semigroup $\left( S(t)\right) _{t\in \mathbb {R}_{+}}$ for any $t\in \mathbb {R}_{+}$ by

$$\begin{aligned} S(t):=e^{-\omega t}P(t). \end{aligned}$$

Then clearly $\left( S(t)\right) _{t\in \mathbb {R}_{+}}$ is also a generalized Feller semigroup and satisfies the conditions of Theorem 4.3. This directly yields the statement of this corollary. $\square$

### Remark 4.6

Notice that quasi-contractivity of the generalized Feller semigroup *P*, or contractivity of the rescaled semigroup *S*, is necessary for the above conclusions: indeed take a deterministic generalized Feller semigroup, i.e., $P_tf(x) = f(\psi _t(x))$, which is not contractive, i.e., there is $x \in E$ and some $t>0$ such that $\rho (\psi _t(x)) > \rho (x)$ but $\rho (\psi _t(\cdot )) \le M \rho$ for some $M>1$ and all $t >0$, then there is obviously no probability measure $\mathbb {P}'_x$ such that

$$\begin{aligned} \mathbb {E}_{\mathbb {P}'_x}\left[ f(\gamma _{t}) \right] = \frac{f(\psi _t(x))\rho (\psi _t(x))}{\rho (x)} \, \end{aligned}$$

holds true. Compare also with Sect. 6.1.

### Remark 4.7

Note that for a monotone concave function $\rho$ and a supermartingale $\left( \lambda _{t}\right) _{t\in \mathbb {R}_{+}}$ we have by Jensen’s inequality

$$\begin{aligned} \mathbb {E}_{x}\left[ \rho \left( \lambda _{t}\right) \right] \le \rho \left( \mathbb {E}_{x}\left[ \left( \lambda _{t}\right) \right] \right) \le \rho \left( \mathbb {E}_{x}\left[ \left( \lambda _{0}\right) \right] \right) =\rho (x); \end{aligned}$$

hence, the condition

$$\begin{aligned} \left\| P(t)\right\| \le 1 \end{aligned}$$

holds true for $\left( P(t)\right) _{t\in \mathbb {R}_{+}}$ defined by $P(t):f\rightarrow \mathbb {E}_{x}\left[ f\left( \lambda _{t}\right) \right]$ for any $t\in \mathbb {R}_{+}$.

Next, we want to compare the laws induced by the corresponding canonical processes in Theorem 3.3 and Theorem 4.3. We here work with the finite time interval $I=[0,T]$ instead of $\mathbb {R}_+$.

### Proposition 4.8

Let $T>0$ and let $I=\left[ 0,T\right]$ and let $\rho$ be Baire-measurable. Let $\left( P(t)\right) _{t\in I}$ be a generalized Feller semigroup on ${\mathscr {B}}^{\rho }(E)$ such that both the conditions of Theorem 3.3 and of Theorem 4.3 are fulfilled and let $\mathbb {\mathbb {P}{}_{\nu }}$ and $\mathbb {P}'_{\nu }$ be the respective probability measures for an initial distribution $\nu$. Let $\left( \lambda _{t}\right) _{t\in I}$ and $\left( \gamma _{t}\right) _{t\in I}$ be the respective canonical processes. Then for $A\in \mathcal {B}(E)^{\left[ 0,T\right] }$

$$\begin{aligned} \mathbb {P}'_{\nu }\left[ A\right] =\mathbb {E}_{\mathbb {P}'_{\nu }}\left[ 1_{A}\right] =\mathbb {E}_{\mathbb {P}{}_{\nu }}\left[ 1_{A}\cdot \frac{\rho (\lambda _{T})}{\rho (\lambda _{0})}\right] , \end{aligned}$$

and

$$\begin{aligned} \mathbb {E}_{\mathbb {P}'_{\nu }}\left[ 1_{A}\cdot \frac{\rho (\gamma _{0})}{\rho (\gamma _{T})}\right] =\mathbb {E}_{\mathbb {P}{}_{\nu }}\left[ 1_{A}\right] =\mathbb {P}{}_{\nu }\left[ A\right] \end{aligned}$$

hold true; hence, $\left. \mathbb {P}'_{\nu }\right| _{\mathcal {B}(E)^{\left[ 0,T\right] }}$ and $\mathbb {P}{}_{\nu }$ are equivalent measures.

### Proof

Let

$$\begin{aligned} \left( p(t)\left( x,\cdot \right) \right) _{t\in I,x{\in }E} \end{aligned}$$

be the family of probability measures such that for all $x\in E$, $t\in \mathbb {R}_{+}$ and $f\in {\mathscr {B}}^{\rho }(E)$

$$\begin{aligned} P(t)f(x)=\int _{E}f(y)p(t)(x,dy). \end{aligned}$$

Just like $\left. \mathbb {P}'_{\nu }\right| _{\mathcal {B}(E)^{\left[ 0,T\right] }}$ the map

$$\begin{aligned} \mathbb {Q}_{\nu }: \mathcal {B}(E)^{\left[ 0,T\right] }&\rightarrow \mathbb {R}_+\\ A&\rightarrow \mathbb {E}_{\mathbb {P}{}_{\nu }}\left[ 1_{A}\cdot \frac{\rho (\lambda _{T})}{\rho (\lambda _{0})}\right] \end{aligned}$$

is a measure on

$$\begin{aligned} \left( E^{\left[ 0,T\right] },\mathcal {B}(E)^{\left[ 0,T\right] }\right) . \end{aligned}$$

Its mass is given by

$$\begin{aligned} \mathbb {E}_{\nu }\left[ 1_{E^{\left[ 0,T\right] }}\frac{\rho (\lambda _{T})}{\rho (\lambda _{0})}\right]&=\int _{E}\left( \int _{E}\rho (x_{T})p(T)(x_{0},dx_{T})\right) \frac{1}{\rho (x_{0})}d\nu (x_{0})\\&=\mathbb {E}'_{\nu }(1_{E}(\gamma _{T}))\\&=\mathbb {P}'_{\nu }(E^{\left[ 0,T\right] }). \end{aligned}$$

It is enough to show that $\mathbb {Q}_{\nu }$ and $\left. \mathbb {P}'_{\nu }\right| _{\mathcal {B}(E)^{\left[ 0,T\right] }}$ coincide on an intersection stable generator of $\mathcal {B}(E)^{\left[ 0,T\right] }$. This is indeed the case as for any $x_{0}\in E$ , $n\in \mathbb {N}$ , $\left\{ t_{1}, \ldots ,t_{n}\right\} \subset \left[ 0,T\right]$, and $A_{t_{1}}, \ldots ,A_{t_{n}}\in \mathcal {B}(E)$ one can approximate the indicator functions $1_{A_{t_{1}}}$, ...,$1_{A_{t_{n}}}$ and $\rho$ using Corollary A.8 by nonnegative continuous bounded functions that converge almost surely with respect to $p_{x_{0}}^{\left\{ 0,t_{1}, \ldots ,t_{n},T\right\} }$, as defined in the proof of Theorem 3.3 and $q_{x_{0}}^{\left\{ 0,t_{1}, \ldots ,t_{n},T\right\} }$, as defined in the proof of Theorem 4.3. Then one obtains by dominated convergence, and the definition of the measures $\mathbb {P}'_{\nu }$ and $\mathbb {P}{}_{\nu }$

$$\begin{aligned}&\mathbb {E}'_{\nu }\left[ 1_{E^{\left[ 0,T\right] }}\cdot 1_{A_{t_{1}}}(\gamma _{t_{1}})\cdot ...\cdot 1_{A_{t_{n}}}(\gamma _{t_{n}})\right] =\, \mathbb {E}'_{\nu }\left[ 1_{E}(\gamma {}_{T})\cdot 1_{A_{t_{1}}}(\gamma _{t_{1}})\cdot ...\cdot 1_{A_{t_{n}}}(\gamma _{t_{n}})\right] \\&\quad =\int _{E}\int _{A_{t_{1}}}...\int _{A_{t_{n}}}\left( \int _{E}\rho (x_{T})p(T-t_{n})(x_{t_{n}},dx_{T})\right) p(t_{n}-t_{n-1})(x_{t_{n-1}},dx_{t_{n}})...\frac{1}{\rho (x_{0})}d\nu (x_{0})\\&\quad = \mathbb {E}_{\mathbb {P}{}_{\nu }}\left[ 1_{E}(\gamma {}_{T})\cdot 1_{A_{t_{1}}}(\gamma _{t_{1}})\cdot ...\cdot 1_{A_{t_{n}}}(\gamma _{t_{n}})\frac{\rho \circ \lambda _{T}}{\rho \circ \lambda _{0}}\right] . \end{aligned}$$

$$\square $$

For $I=\mathbb {R}_{+}$ or $I=\left[ 0,T\right] \subset \mathbb {R}_{+}$ let $\left( P(t)\right) _{t\in \mathbb {R}_{+}}$ be a generalized Feller semigroup and $\left( \lambda _{t}^{x}\right) _{t\in I,\,x\in E}$ the family of generalized Feller processes constructed according to Theorem 3.3, such that $\mathbb {P}_x\left( \lambda _{0}^{x}=x\right) =1$ and

$$\begin{aligned} \mathbb {E}_x\left[ \left. f(\lambda ^x_{t})\right| \mathcal {F}_{s}\right] =P\left( t-s\right) f(\lambda ^x_{s}) \end{aligned}$$

holds true. Similarly we consider a family of extended Feller processes $\left( \gamma _{t}^{x}\right) _{t\in I,\,x\in E}$ such that $\mathbb {P}^{'}_x\left( \gamma _{0}^{x}=x\right) =1$ and

$$\begin{aligned} \mathbb {E}^{'}_x\left[ \left. f(\gamma ^x_{t})\right| \mathcal {F}_{s}\right] =\frac{P\left( t-s\right) (f\cdot \rho )}{\rho }(\gamma ^x_{s}) \end{aligned}$$

holds true. For convenience, we here work on one probability space $(\Omega ,\mathcal {F},\mathbb {P})$ for the whole family of $(\lambda _{t}^{x})_{t\in I,\,x\in E}$ and on $(\Omega ,\mathcal {F},\mathbb {P}^{'})$ for $(\gamma _{t}^{x})_{t\in I,\,x\in E}$, respectively. The goal is to make the measure change between $\mathbb {P}$ and $\mathbb {P}^{'}$ in the case of diffusion processes precise. Note that this can again be compared with Doob’s h-transform with an analogous change of the drift and appearance of a killing rate, see, e.g., [22].

### Proposition 4.9

Let $\left( P(t)\right) _{t\in I}$ be such that the conditions of both Theorem 3.3 and Theorem 4.3 are fulfilled. Let $I=\mathbb {R}_{+}$ or $I=\left[ 0,T\right] \subset \mathbb {R}_{+}$ and let $\left( \lambda _{t}^{x}\right) _{t\in I,x\in \mathbb {R}^{d}}$ be a family of Ito diffusions on the filtered probability space $\left( \Omega ,\mathcal {F},\left( \mathcal {F}_{t}\right) _{t\in I},\mathbb {P}\right)$ with state space $\mathbb {R}^{d}$, $d\in \mathbb {N}$, with drift $\mu$ and diffusion matrix $\sigma$ such that $\lambda _{0}^{x}=x$ $\mathbb {P}$-a.s for any $x\in \mathbb {R}^{d}$, i.e., we consider a solution to the following SDE

$$\begin{aligned} d\lambda _t^x= \mu (\lambda _t) dt + \sigma (\lambda _t^x) dW_t, \quad \lambda _0^x= x, \end{aligned}$$

where *W* is a *d*-dimensional Brownian motion, $\mu : \mathbb {R}^d \rightarrow \mathbb {R}^d$ and $\sigma : \mathbb {R}^d \rightarrow \mathbb {R}^{d \times d}$ are continuous functions. Let $\rho \in C^{2}(\mathbb {R}^{d})$ and suppose that

4.4 $$\begin{aligned} \left( \int _0^t \nabla _x^{\top } \rho (\lambda _s^x) \sigma (\lambda _s^x) dW_s\right) _{t \in I} \text { is a true martingale.} \end{aligned}$$

Moreover, let $\left( P(t)\right) _{t\in I}$ be the semigroup on ${\mathscr {B}}^{\rho }(\mathbb {R}^{d})$ defined by

4.5 $$\begin{aligned} P(t)f(x):=\mathbb {E}\left[ f(\lambda _{t}^{x})\right]  &   \text {for } f\in {\mathscr {B}}^{\rho }(\mathbb {R}^{d}). \end{aligned}$$

Moreover, let $\mathbb {P}'$ be another probability measure on $\left( \Omega ,\mathcal {F},\left( \mathcal {F}_{t}\right) _{t\in I}\right)$ such that for the family of Markov processes $\left( \gamma _{t}^{x}\right) _{t\in I,x\in \mathbb {R}^{d}}$ with $\gamma _{0}^{x}=x \mathbb {P}'$-a.s. for any $x\in \mathbb {R}^{d}$, $t\in I$ and any real-valued map *f* on $\mathbb {R}^{d}\cup \left\{ \Delta \right\}$ that is bounded and Borel-measurable

$$\begin{aligned} \mathbb {E}'\left[ f(\gamma _{t}^{x})\right] =\frac{P\left( t\right) \left( f\cdot \rho \right) }{\rho }(x) \end{aligned}$$

holds true.

Then the drift $\mu '=\left( \mu _{1}', \ldots ,\mu _{d}'\right)$ of $\left( \gamma _{t}\right) _{t\in I}$ with respect to $\mathbb {P}'$ is given by

$$\begin{aligned} \mu _{i}'=\mu _{i}+{\sum }_{j=1}^{d}\frac{d\rho }{dx_{j}}(x)\frac{\sigma _{ij}^{2}(x)}{\rho (x)}, \end{aligned}$$

the diffusion matrix is $\sigma '=\sigma$, and the killing rate $c'<0$ is

$$\begin{aligned} c'(x)&=\left( {\sum }_{i=1}^{d}\frac{d\rho }{dx_{i}}(x)\mu _{i}(x)+\frac{1}{2}{\sum }_{j=1}^{d}{\sum }_{i=1}^{d}\frac{d^{2}\rho }{dx_{i}dx_{j}}(x)\sigma _{ij}^{2}(x)\right) \frac{1}{\rho (x)}. \end{aligned}$$

### Proof

We note that due to metrizability on $\mathbb {R}^{d}\cup \left\{ \Delta \right\}$ Baire and Borel $\sigma$-algebras coincide. Furthermore, since by assumption the conditions of Theorem 4.3 are fulfilled the semigroup $\left( P(t)\right) _{t\in I}$ is contractive. By Ito’s formula and the Assumption (4.4) as well as $\left\| P(t)\right\| _{L({\mathscr {B}}^{\rho }(E))}\le 1$, we have for any $x\in E$ and $t\in I$

$$\begin{aligned} \mathbb {E}\left[ \rho (\lambda _{t}^{x})\right]&=\rho (x)+\int _{0}^{t}\left( {\sum }_{i=1}^{d}\frac{d\rho }{dx_{i}}(x)\mu _{i}(x)+\frac{1}{2}{\sum }_{j=1}^{d}{\sum }_{i=1}^{d}\frac{d^{2}\rho }{dx_{i}dx_{j}}(x)\sigma _{ij}^{2}(x)\right) ds\\&\le \rho (x). \end{aligned}$$

Hence,

$$\begin{aligned} {\sum }_{i=1}^{d}\frac{d\rho }{dx_{i}}(x)\mu _{i}(x)+\frac{1}{2}{\sum }_{j=1}^{d}{\sum }_{i=1}^{d}\frac{d^{2}\rho }{dx_{i}dx_{j}}(x)\sigma _{ij}^{2}(x)\le 0 \end{aligned}$$

which yields the sign of the killing rate $c'$. Furthermore, for any $x\in E$ and $f\in C_{c}^{2}(E)$ the infinitesimal generator $\mathscr {A}'$ of $\left( \gamma _{t}\right) _{t\in I}$ is given by

$$\begin{aligned}&\mathscr {A}'f(x)\\&\quad = \underset{t\searrow 0}{\lim }\,\frac{\mathbb {E}'\left[ f(\gamma _{t}^{x})\right] -f(x)}{t}\\&\quad = \underset{t\searrow 0}{\lim }\,\frac{1}{t}\left( \frac{P\left( t\right) \left( f\cdot \rho \right) }{\rho }(x)-f(x)\right) \\&\quad = \underset{t\searrow 0}{\lim }\,\frac{1}{t}\left( \frac{\mathbb {E}\left[ (f\cdot \rho )(\lambda _{t}^{x})\right] }{\rho (x)}-f(x)\right) \\&\quad = \underset{t\searrow 0}{\lim }\,\frac{1}{t}\left( \frac{(f\cdot \rho )(x)+\int _{0}^{t}{\sum }_{i=1}^{d}\left( \frac{d(f\cdot \rho )}{dx_{i}}\mu _{i}(\lambda _{s}^x)\right) ds+\frac{1}{2}\int _{0}^{t}{\sum }_{j=1}^{d}{\sum }_{i=1}^{d}\left( \frac{d^{2}(f\cdot \rho )}{dx_{i}dx_{j}}\sigma _{ij}^{2}(\lambda _{s}^x)\right) ds}{\rho (x)}-f(x)\right) \\&\quad = {\sum }_{i=1}^{d}\frac{d(f\cdot \rho )}{dx_{i}}(x)\frac{\mu _{i}(x)}{\rho (x)}+\frac{1}{2}\,{\sum }_{j=1}^{d}{\sum }_{i=1}^{d}\frac{d^{2}(f\cdot \rho )}{dx_{i}dx_{j}}(x)\frac{\sigma _{ij}^{2}(x)}{\rho (x)}. \end{aligned}$$

Applying the product rule and bringing $\mathscr {A}'f(x)$ into the following form

$$\begin{aligned} \mathscr {A}'f(x) = \sum _{i=1}^d \frac{df}{dx_i} \mu _i'(x) + \frac{1}{2}\sum _{i,j=1}^d \frac{d^2f}{dx_{ij}} \sigma _{ij}'(x) + f(x)c'(x) \end{aligned}$$

yields the assertion of the proposition. $\square$

As we will see in the next section, extended Feller processes are a generalization of Feller processes to more general state spaces. Thus, it is not surprising that we obtain a regularity result for their paths. In order to state this result, we consider the following space that will also be needed in the next section:

### Definition 4.10

For $\rho$ being measurable with respect to the Baire $\sigma$-algebra $\mathcal {B}_0(E)$, we define

$$\begin{aligned} \ell ^{\rho }(E):=\left\{ \frac{f}{\rho }:\,f\in {\mathscr {B}}^{\rho }(E)\right\} . \end{aligned}$$

### Remark 4.11

Note that $\ell ^{\rho }(E)$ is a Banach space with respect to $\left\| \cdot \right\| _{\infty }.$

According to Theorem 4.3, the semigroup $\left( Q(t)\right) _{t\in \mathbb {R}_{+}}$ on $\ell ^{\rho }(E)$ defined by

$$\begin{aligned} Q(t)f:=\frac{P(t)\left( f\cdot \rho \right) }{\rho } \end{aligned}$$

is strongly continuous, contractive and positive.

In order to show regularity of the paths of $f(\gamma _{t})$ for any $f\in \ell ^{\rho }(E)$, one can proceed as in the proof of Theorem 3.4. This yields the following statement:

### Theorem 4.12

Let $\left( P(t)\right) _{t\in \mathbb {R}_{+}}$ be a generalized *Feller* semigroup on ${\mathscr {B}}^{\rho }(E)$, let the conditions of Theorem 4.3 be satisfied and let $\left( \gamma _{t}\right) _{t\in \mathbb {R}_{+}}$ be the corresponding stochastic process on

$$\begin{aligned} \left( \left( E\cup \left\{ \triangle \right\} \right) ^{\mathbb {R}_{+}},\mathcal {B}(E\cup \left\{ \Delta \right\} )^{\mathbb {R}_{+}}\right) . \end{aligned}$$

Then the following hold:

(i) $\left( {f(\gamma _{t})}\right) _{t\in \mathbb {R}_{+}}$ has a version with càdlàg paths for every $f\in \ell ^{\rho }(E\cup \left\{ \triangle \right\} )$

(ii) If additionally $t\rightarrow P(t)\rho (x)$ is continuous for $\nu$-almost any $x\in E$ and there exists a countable family $\left( f_{n}\right) _{n\in \mathbb {N}}\subset \ell ^{\rho }(E\cup \left\{ \triangle \right\} )$ whose span is dense, then $\left( \gamma _{t}\right) _{t\in \mathbb {R}_{+}}$ has a version with càdlàg paths.

## Relation to Feller processes

In this section, again $\left( E,\,\rho \right)$ denotes a weighted space equipped with the Borel $\sigma$-algebra $\mathcal {B}(E)$. We investigate the relationship between generalized and extended Feller processes as of Theorems 3.3 and 4.3, respectively, and Feller processes.

We recall that a *classical Feller semigroup* $\left( Q(t)\right) _{t\in \mathbb {R}_{+}}$ is defined on a metrizable, separable, locally compact Hausdorff space *K* as a strongly continuous, positive, contractive semigroup acting on the space $C_0(K)$. A *classical Feller process* is then a Markov process such that the map

$$\begin{aligned} f \rightarrow \int _{K}f(y)p(t)(\cdot ,dy), \end{aligned}$$

is a classical Feller semigroup. As already mentioned in Introduction, we shall speak just of *Feller processes* (in contrast to *classical Feller processes* and slightly generalizing the classical definition) if the state space is locally compact but not necessarily metrizable or separable.

In the following proposition, we provide a condition under which Feller processes are generalized Feller processes.

### Proposition 5.1

Let $\left( E,\rho \right)$ be a weighted space and *E* be locally compact. Let $\left( \lambda _{t}\right) _{t\in \mathbb {R}_{+}}$ be a Feller process on *E* with semigroup of transition probabilities $\left( p(t)\right) _{t\in \mathbb {R}_{+}}$ on $\left( E,\mathcal {B}(E)\right)$ with initial distribution $\nu \in \mathcal {M}^{\rho }(E)$. Let there be $t_{0}>0$ and $C>0$ such that for all $x\in E$ and $0\le t\le t_{0}$

$$\begin{aligned} \mathbb {E}_{x}\left[ \rho (\lambda _{t})\right] \le C\rho (x). \end{aligned}$$

Then $\left( \lambda _{t}\right) _{t\in \mathbb {R}_{+}}$ is a generalized Feller process and for any $x\in E$ and initial distribution $\delta _x$ the process is also a generalized Feller process with respect to the right continuous extension of its natural filtration.

### Proof

We first show that $\left( \tilde{P}(t)\right) _{t\in \mathbb {R}_{+}}$ given by

$$\begin{aligned} \tilde{P}(t):\,\,{\mathscr {B}}^{\rho }(E)&\rightarrow {\mathscr {B}}^{\rho }(E)\\ f&\rightarrow \int _{E}f(y)p(t)(\cdot ,dy) \end{aligned}$$

is a generalized Feller semigroup knowing that $\left( P(t)\right) _{t\in \mathbb {R}_{+}}$ given by $P(t): C_{0}(E) \rightarrow C_{0}(E), \, f \rightarrow \int _{E}f(y)p(t)(\cdot ,dy)$ is a Feller semigroup. To this end, we show that $\tilde{P}(t)$ is linear bounded map satisfying $\tilde{P}(t)\left( {\mathscr {B}}^{\rho }(E)\right) ={\mathscr {B}}^{\rho }(E)$. For any $f\in {\mathscr {B}}^{\rho }(E)$ and $0\le t\le t_{0}$

$$\begin{aligned} \tilde{P}(t)f(x)&=\int _{E}f(y)p(t)(x,dy)\\&=\int _{E}\frac{f(y)}{\rho (y)}\rho (y)p(t)(x,dy)\\&\le \left\| f\right\| _{\rho }C\rho (x), \end{aligned}$$

proving that for $0\le t\le t_{0}$ $\tilde{P}(t)$ is a linear bounded map with $\left\| \tilde{P}(t)\right\| _{L\left( \mathscr {B}^{\rho }(E)\right) }\le C.$ By Lemma A.11, we know that for any $\varepsilon >0$ and any $f\in \mathscr {B}^{\rho }(E)$ there exists some $g_{\varepsilon }\in C_{0}(E)$ such that $\left\| f-g_{\varepsilon }\right\| _{\rho }<\varepsilon .$ Hence for $0\le t\le t_{0}$

$$\begin{aligned} \left\| \tilde{P}(t)f-\tilde{P}(t)g_{\varepsilon }\right\| _{\rho }<C\varepsilon \end{aligned}$$

and since $\tilde{P}(t)g_{\varepsilon }=P(t)g_{\varepsilon }\in C_{0}(E)$ it follows that $\tilde{P}(t)({\mathscr {B}}^{\rho }(E))={\mathscr {B}}^{\rho }(E).$ For any $s>0$, there is $n\in \mathbb {N}$ such that $s/n<t_{0}$ and since $\left( p(t)\right) _{t\in \mathbb {R}_{+}}$ is a semigroup of transition probabilities on $\left( E,\mathcal {B}(E)\right)$

$$\begin{aligned} \tilde{P}(s)f(x)&=\int _{E}f(y)p(\frac{s}{n}+ \cdots +\frac{s}{n})(x,dy)\\&=\left( \tilde{P}(\frac{s}{n})...\left( \tilde{P}(\frac{s}{n})f\right) \right) (x). \end{aligned}$$

Hence, $\tilde{P}(t)$ is a linear bounded map for any $t>0$ andIn order to show that $\tilde{P}(t)$ is indeed a generalized Feller semigroup we have to show the properties **P1**, ...,**P5** from Definition 2.7 hold. **P1** and **P2** follow immediately from the fact $\left( p(t)\right) _{t\in \mathbb {R}_{+}}$ is a semigroup of transition probabilities. **P4** follows by assumption and positivity (**P5**) is obvious. It remains to be shown that for all $f\in {\mathscr {B}}^{\rho }(E)$ and all $x\in E$

$$\begin{aligned} \underset{t\searrow 0}{\lim }\,\tilde{P}(t)f(x)=f(x). \end{aligned}$$

Fix $f\in {\mathscr {B}}^{\rho }(E)$ and $x\in E$. Again by Lemma A.11, we know that for any $\varepsilon >0$ there is $g_{\varepsilon }\in C_{0}(E)$ such that $\left\| f-g_{\varepsilon }\right\| _{\rho }<\varepsilon .$ As by the Feller property

$$\begin{aligned} \underset{t\searrow 0}{\lim }\ |\tilde{P}(t)g_{\varepsilon }(x)-g_{\varepsilon }(x)|=0 \end{aligned}$$

for all $x \in E$, we thus have

$$\begin{aligned} \underset{t\searrow 0}{\lim }\,\left| \tilde{P}(t)f(x)-f(x)\right|&= \underset{t\searrow 0}{\lim }\,\left| \tilde{P}(t)f(x)-\tilde{P}(t)g_{\varepsilon }(x)\right| +\underset{t\searrow 0}{\lim }\,\left| \tilde{P}(t)g_{\varepsilon }(x)-g_{\varepsilon }(x)\right| \\&\quad +\left| g_{\varepsilon }(x)-f(x)\right| \\&\le \underset{t\searrow 0}{\lim }\,\left\| \tilde{P}(t)\right\| _{L\left( \mathcal {B}^{\rho }(E)\right) }\left\| f-g_{\varepsilon }\right\| _{\rho }\rho (x) +\left| g_{\varepsilon }(x)-f(x)\right| \\&\le C\varepsilon \rho (x)+\varepsilon \rho (x). \end{aligned}$$

Since $\varepsilon >0$ was arbitrary, $\left( \tilde{P}(t)\right) _{t\in \mathbb {R}_{+}}$ is a generalized Feller semigroup. Finally, since $\left( \lambda _{t}\right) _{t\in \mathbb {R}_{+}}$ is a Markov process with respect to its natural filtration $\left( \mathcal {F}{}_{t}^{0}\right) _{t\in \mathbb {R}_{+}}$ for any initial distribution $\nu$ and $f\in {\mathscr {B}}^{\rho }(E)$ and $0\le s\le t$ it holds

$$\begin{aligned} \mathbb {E}_{\mathbb {P}_{\nu }}\left[ \left. f(\lambda _{t})\right| \mathcal {F}{}_{s}^{0}\right] =\int _{E}f(y)p(t-s)(\lambda _{s},dy)=\tilde{P}(t-s)f(\lambda _{s}) \end{aligned}$$

$\mathbb {P}_{\nu }$ -almost surely. As in the last step of the proof in Theorem 3.3, for any $x\in E$ and initial distribution $\delta _x$ this equation can be extended to the right continuous extension of $\left( \mathcal {F}{}_{t}^{0}\right) _{t\in \mathbb {R}_{+}}$. This yields the statement of the proposition. $\square$

The following proposition yields an isometric isomorphism that we use later as a tool to link generalized Feller semigroups to Feller semigroups. For its formulation, recall Definition 4.10.

### Proposition 5.2

Let $\rho$ be an admissible weight function. Define

$$\begin{aligned} \Phi :\,&L({\mathscr {B}}^{\rho }(E))\rightarrow L(\ell ^{\rho }(E))\\&P \rightarrow \frac{P\left( \left( \cdot \right) \cdot \rho \right) }{\rho }. \end{aligned}$$

Then, ${\Phi }$ is an isometric isomorphism between $L({\mathscr {B}}^{\rho }(E))$ and $L(\ell ^{\rho }(E))$.

### Proof

Clearly,$\frac{P\left( \left( \cdot \right) \cdot \rho \right) }{\rho }\in L(\ell ^{\rho }(E))$ is well defined and $\Phi$ is linear. We show first that ${\Phi }$ is an isometry. We calculate for any $f \in \ell ^{\rho }(E)$

$$\begin{aligned} \left\| \left( \Phi P\right) f\right\| _{\infty }&=\left\| P\left( f\cdot \rho \right) \right\| _{{\rho }}\\&\le \left\| P\right\| _{L\left( \mathscr {B}^{\rho }(E)\right) }\cdot \left\| f\right\| _{\infty }. \end{aligned}$$

Hence

$$\begin{aligned} \left\| \left( \Phi P\right) \right\| _{L(\ell ^{\rho }(E))}\le \left\| P\right\| _{L\left( \mathscr {B}^{\rho }(E)\right) }. \end{aligned}$$

Furthermore, for $\varepsilon >0$ let $g_{\varepsilon }\in \mathscr {B}^{\rho }(E)$ be such that

$$\begin{aligned} \left\| Pg_{\varepsilon }\right\| _{{\rho }}\ge \left( \left\| P\right\| _{L\left( \mathscr {B}^{\rho }(E)\right) }-\varepsilon \right) \left\| g_{\varepsilon }\right\| _{{\rho }}. \end{aligned}$$

Then $$\frac{g_{\varepsilon }}{\rho }\in \ell ^{\rho }(E)$$ and

$$\begin{aligned} \left\| (\Phi P)\left( \frac{g_{\varepsilon }}{\rho }\right) \right\| _{\infty }&=\left\| Pg_{\varepsilon }\right\| _{\rho }\\&\ge \left( \left\| P\right\| _{L({\mathscr {B}}^{\rho }(E))}-\varepsilon \right) \left\| \frac{g_{\varepsilon }}{\rho }\right\| _{{\infty }}, \end{aligned}$$

which shows that

$$\begin{aligned} \left\| \left( \Phi P\right) \right\| _{L(\ell ^{\rho }(E))}\ge \left\| P\right\| _{L\left( \mathscr {B}^{\rho }(E)\right) }-\varepsilon . \end{aligned}$$

Thus, $\Phi$ is an isometry. Furthermore, we note that for any $Q\in L(\ell ^{\rho }(E))$ the map

$$\begin{aligned} Q'(\cdot ):=Q\left( \frac{(\cdot )}{\rho }\right) \cdot \rho \end{aligned}$$

yields surjectivity of $\Phi$ via $\Phi \left( Q'\right) =Q$.

$$\square $$

### Corollary 5.3

There is an isometric isomorphism between contractive generalized Feller semigroups on ${\mathscr {B}}^{\rho }(E)$ and strongly continuous, contractive, positive semigroups on $\ell ^{\rho }(E)$.

### Proof

Use Proposition 5.2 above and strong continuity of generalized Feller semigroups (see Theorem 2.8). The respective required semigroup properties follow immediately. $\square$

### Lemma 5.4

If the admissible weight function $\rho$ is continuous, then $C_{0}(E)=\ell ^{\rho }(E)$.

### Proof

This follows from Lemma 2.3 (iii) and (iv). $\square$

### Corollary 5.5

If the admissible weight function $\rho$ is continuous, then there is an isometric isomorphism between contractive generalized Feller semigroups on ${\mathscr {B}}^{\rho }(E)$ and Feller semigroups on $C_{0}(E)$.

### Proof

Due to the continuity of $\rho$, *E* is locally compact (see Lemma 2.3 (i)). Therefore, Feller semigroups are well defined. The rest follows from Lemma 5.4 and Proposition 5.2. $\square$

The following theorem is the reason why $\left( \gamma _{t}\right) _{t\in \mathbb {R}_{+}}$ was named $extended Feller process$.

### Theorem 5.6

Let $\left( E,\rho \right)$ be a weighted space and let $\rho$ be continuous. Let $\left( P(t)\right) _{t\in \mathbb {R}_{+}}$ be a generalized Feller semigroup on ${\mathscr {B}}^{\rho }(E)$ and let $\omega \in \mathbb {R}$ be such that for any $t\in \mathbb {R}_{+}$

$$\begin{aligned} \left\| P(t)\right\| _{L({\mathscr {B}}^{\rho }(E))}\le e^{\omega t}. \end{aligned}$$

Then $\left( Q(t)\right) _{t\in \mathbb {R}_{+}}$ defined as

$$\begin{aligned} Q(t)f:=e^{-\omega t}\frac{P\left( t\right) \left( f\cdot \rho \right) }{\rho } \end{aligned}$$

is a strongly continuous, positive, contractive semigroup on $C_{0}(E)$^2^ and for any probability measure $\nu$ on $\left( E,\mathcal {B}(E)\right)$ there exists a probability measure $\mathbb {P}'_{\nu }$ on

$$\begin{aligned} \left( E^{\mathbb {R}_{+}},\mathcal {B}(E)^{\mathbb {R}_{+}}\right) \end{aligned}$$

and a right continuous filtration $\left( \mathcal {F}_{t}\right) _{t\in \mathbb {R}_{+}}$ such that and for any $t\ge s\ge 0$ and for any $f\in C_{0}(E)$ for the canonical process $\left( \gamma _{t}\right) _{t\in \mathbb {R}_{+}}$

5.1 $$\begin{aligned} \mathbb {E}_{\mathbb {P}'_{\nu }}\left[ \left. f(\gamma _{t})\right| \mathcal {F}_{s}\right] =Q(t-s)f(\gamma _{s}) \end{aligned}$$

holds true $\mathbb {P}'_{\nu }$ - almost surely and

$$\begin{aligned} \mathbb {P}'_{\nu }\circ \gamma _{\text {0}}^{-1}=\nu \end{aligned}$$

holds true.

### Proof

This follows from Lemma 2.3, Theorem 4.3, Corollary 4.5 and Proposition 5.2. $\square$

## Examples

This section is dedicated to specific examples ranging from deterministic Feller processes corresponding to semigroups of transport type to affine and polynomial processes. In Sect. 6.3, we also show by means of a counterexample the necessity of Condition **P4** (which is not needed for classical Feller semigroups on $C_0(E)$) to obtain strong continuity for the generalized Feller semigroup.

### Generalized Feller processes of transport type

We consider a generalized Feller semigroup such that the corresponding generalized Feller process, given by $\left( \psi _{t}\right) _{t\in \mathbb {R}_{+}}$, is deterministic. This is an important class of examples, since it connects the theory of ordinary differential equations on weighted spaces with generalized Feller processes. In other contexts, e.g., in machine learning, this is related to the so-called Koopman operators, or, e.g., in the theory of partial differential equations of transport type, to the method of characteristics.

Let us start by introducing the notion of a smooth algebra homomorphism. Notice that we cannot simply define the standard algebra homomorphism property, since ${\mathscr {B}}^{\rho }(E)$ is not an algebra. Therefore, we rather use the notion of a smooth algebra homomorphism. It serves our purpose to characterize point evaluations.

#### Definition 6.1

We call a continuous linear functional $\ell : {\mathscr {B}}^{\rho }(E) \rightarrow \mathbb {R}$ a *smooth algebra homomorphism,* if for all bounded smooth functions $\phi :\mathbb {R}^n \rightarrow \mathbb {R}$ and for all $f_1,\ldots ,f_n \in {\mathscr {B}}^{\rho }(E)$ we have that

$$\begin{aligned} \ell (\phi (f_1(\cdot ),\ldots ,f_n(\cdot ))) = \phi (\ell (f_1),\ldots ,\ell (f_n)) \,. \end{aligned}$$

Analogously, we call a continuous linear map $P: {\mathscr {B}}^{\rho }(E) \rightarrow {\mathscr {B}}^{\rho }(E)$ a *smooth operator algebra homomorphism* if for all bounded smooth functions $\phi :\mathbb {R}^n \rightarrow \mathbb {R}$ and for all $f_1,\ldots ,f_n \in {\mathscr {B}}^{\rho }(E)$ we have that

6.1 $$\begin{aligned} P(\phi (f_1(\cdot ),\ldots ,f_n(\cdot )))(\cdot ) = \phi (P(f_1)(\cdot ),\ldots ,\ell (f_n)(\cdot )) \, . \end{aligned}$$

The next lemma states that smooth algebra homomorphisms are characterized as point evaluations.

#### Lemma 6.2

Let *E* be a weighted space and $\ell : {\mathscr {B}}^{\rho }(E) \rightarrow \mathbb {R}$ be a continuous linear functional. Then $\ell$ is a smooth algebra homomorphism if and only if there is $x \in E$ such that $\ell (f) = f(x)$ for all $f \in {\mathscr {B}}^{\rho }(E)$. The point *x* is uniquely determined by $\ell$.

#### Proof

Clearly, if $\ell$ is given by a point evaluation, it also satisfies the smooth algebra homomorphism property.

For the converse direction, observe that a smooth algebra homomorphism can never be a sum of two other non-trivial continuous linear functionals with disjoint supports. Indeed assume $\ell = \ell _1 + \ell _2$ with $\ell _j \ne 0$, $j=1,2$ with disjoint supports, then with $\phi (f_1,f_2)=f_1f_2$ (for some bounded $f_1$, $f_2$), $\ell$ would need to satisfy

$$\begin{aligned} \ell _1(f_1f_2) + \ell _2(f_1f_2) = (\ell _1(f_1)+\ell _2(f_1))(\ell _1(f_2)+\ell _2(f_2)). \end{aligned}$$

This is impossible if one chooses $f_i$ with vanishing $f_1f_2$ on the support of $\ell$ such that $\ell _i(f_i)=1$ and $\ell _1(f_2)=\ell _2(f_1)=0$, since then the left-hand side gives 0 while the right-hand side is equal to 1. Note that one can always construct such $f_i$, as *E* is completely regular and $\sigma$-compact, whence normal. Therefore, the measure representing $\ell$ has to have as support only a singleton. $\square$

We can now use this to characterize smooth operator algebra homomorphisms via concatenations with maps $\psi$ whose restrictions to compact sets $K_R$ are continuous.

#### Lemma 6.3

Consider a map $P:{\mathscr {B}}^{\rho }(E) \rightarrow {\mathscr {B}}^{\rho }(E)$. Then *P* is a smooth operator algebra homomorphims if and only if there is a map $\psi : E \rightarrow E$, whose restrictions $\psi |_{K_R}$ are continuous for all $R >0$ and which satisfies

6.2 $$\begin{aligned} \sup _{x \in E} \frac{\rho \circ \psi (x)}{\rho (x)} < \infty , \end{aligned}$$

such that $Pf=f \circ \psi$. The map $\psi$ is uniquely determined by *P*.

#### Proof

Assume first that *P* is given by $Pf=f \circ \psi$. Then clearly *P* is linear and satisfies (6.1). Continuity follows since for a sequence $(f_n)$ converging to *f* in ${\mathscr {B}}^{\rho }(E)$ it holds that for every $\varepsilon >0$ there exists some *N* such that

$$\begin{aligned} \Vert Pf-Pf_N\Vert _{\rho }= &   \underset{x\in E}{\sup }\,\frac{\left| f\circ \psi _{t}(x)-f_{N}\circ \psi (x)\right| }{\rho (x)} \\  = &   \underset{x\in E}{\sup }\,\frac{\left| f\circ \psi (x)-f_{N}\circ \psi (x)\right| }{\rho \circ \psi (x)}\cdot \frac{\rho \circ \psi (x)}{\rho (x)} < \varepsilon , \end{aligned}$$

where the last inequaltiy follows from (6.2).

Conversely, if *P* is a smooth operator algebra homomorphism, then existence of $\psi$ is a consequence of the previous lemma. The proof of the continuity of $\psi |_{K_R}$ for all $R >0$ and (6.2) follows similarly as the necessity of Condition (iv) and (v) in Proposition 6.6. $\square$

#### Definition 6.4

A linear operator $A: \operatorname {dom}(A) \subset {\mathscr {B}}^{\rho }(E) \rightarrow {\mathscr {B}}^{\rho }(E)$ is called smooth derivation if for all smooth $\phi : \mathbb {R}^n \rightarrow \mathbb {R}$ with $\phi (f_1(\cdot ),\ldots ,f_n(\cdot )) \in \operatorname {dom}(A)$ for $f_1,\ldots ,f_n \in \operatorname {dom}(A)$ we have that

$$\begin{aligned} A(\phi (f_1(\cdot ),\ldots ,f_n(\cdot ))) = \sum _{i=1}^n (\partial _i \phi )(f_1(\cdot ),\ldots ,f_n(\cdot )) A f_i(\cdot ) \,. \end{aligned}$$

With this definition, we can already characterize how a generalized Feller semigroup of smooth operator algebra homomorphism is generated.

#### Proposition 6.5

Let $\left( P(t)\right) _{t\in \mathbb {R}_{+}}$ be a generalized Feller semigroup. Then *P* is a semigroup of smooth operator algebra homomorphisms if and only if the generator *A* is a smooth derivation such that for every smooth map $\phi : \mathbb {R}^n \rightarrow \mathbb {R}$ with globally bounded first derivatives and for every $f_1,\ldots ,f_n \in \operatorname {dom}(A)$ we have $\phi (f_1(\cdot ),\ldots ,f_n(\cdot )) \in \operatorname {dom}(A)$. For every $t \ge 0$, such a generalized Feller semigroup is of the form

$$\begin{aligned} P(t)(f):=f\circ \psi _{t} \end{aligned}$$

for some map $\psi _t:E \rightarrow E$ which satisfies the properties (i) - (vi) of Proposition 6.6.

#### Proof

Let *P* be a generalized Feller semigroup of smooth operator algebra homomorphisms. Consider some smooth $\phi : \mathbb {R}^n \rightarrow \mathbb {R}$ with $\phi (f_1(\cdot ),\ldots ,f_n(\cdot )) \in \operatorname {dom}(A)$ with $f_1,\ldots ,f_n \in \operatorname {dom}(A)$. Then by the smooth operator algebra homomorphism property and the chain rule, we have

$$\begin{aligned} A\phi (f_1(\cdot ),\ldots ,f_n(\cdot ))&:= \frac{d}{dt}|_{t=0}P(t) \phi (f_1(\cdot ),\ldots ,f_n(\cdot ))\\  &=\frac{d}{dt}|_{t=0}\phi (P(t)f_1(\cdot ),\ldots ,P(t)f_n(\cdot ))\\&=\sum _{i=1}^n (\partial _i \phi )(f_1(\cdot ),\ldots ,f_n(\cdot )) A f_i, \end{aligned}$$

showing that the generator *A* is a smooth derivation. If $\phi$ has globally bounded derivatives, the right-hand side lies in ${\mathscr {B}}^{\rho }(E)$, and therefore, $\phi (f_1(\cdot ),\ldots ,f_n(\cdot )) \in \operatorname {dom}(A)$, which proves the first direction.

Conversely, for fixed $t > 0$, $0 \le s \le t$, a smooth map $\phi : \mathbb {R}^n \rightarrow \mathbb {R}$ with globally bounded first derivatives, and some $f_1,\ldots ,f_n \in \operatorname {dom}(A)$ with $\phi (f_1(\cdot ),\ldots ,f_n(\cdot )) \in \operatorname {dom}(A)$ we obtain by the smooth derivation property of *A* that

$$\begin{aligned}&\frac{d}{ds} P(s)\phi (P(t-s)f_1, \ldots , P(t-s)f_n) \\&\quad = P(s) A \phi (P(t-s)f_1, \ldots , P(t-s)f_n) \\  &\quad \quad - P(s)(\sum _{i=1}^n (\partial _i \phi )(P(t-s)f_1,\ldots ,P(t-s)f_n) A P(t-s) f_i)\\&\quad =P(s)(\sum _{i=1}^n (\partial _i \phi )(P(t-s)f_1,\ldots ,P(t-s)f_n) A P(t-s) f_i) \\  &\quad \quad - P(s)(\sum _{i=1}^n (\partial _i \phi )(P(t-s)f_1,\ldots ,P(t-s)f_n) A P(t-s) f_i)=0 \, . \end{aligned}$$

Therefore, $s \mapsto P(s)\phi (P(t-s)f_1, \ldots , P(t-s)f_n)$ is constant and by setting $s=t$ and $s=0$ we get $P(t)\phi (f_1,\ldots , f_n) = \phi (P(t) f_1,\ldots , P(t) f_n)$. Since this holds on a dense subset of functions $\phi$ with globally bounded first derivatives, we can conclude.

Concerning the last assertion Lemma 6.3 yields for every fixed $t >0$ a map $\psi _t$ such that $P(t)f(x)=f \circ \psi _t(x)$. Since *P* is assumed to be a generalized Feller semigroup, the properties of $\psi _t$ follow from Proposition 6.6. $\square$

The above proposition characterizes the generators of generalized Feller semigroups of smooth operator algebra homomorphisms as smooth derivations, which are also called *transport operators* motivating the name of the current subsection. Indeed, instead of generalized Feller semigroups of smooth operator algebra homomorphisms we can thus also speak of *generalized Feller semigroup of transport type.* We shall use the notions smooth derivations and transport operators interchangeably. The following proposition establishes all properties of the transport map $\psi _t$ for $t \in \mathbb {R}_+$. It is remarkable that we only need continuity properties of $\psi$ with respect to time and space.

#### Proposition 6.6

Let $\left( \psi _{t}\right) _{t\in \mathbb {R}_{+}}$ be a family of maps from *E* to *E*. Then $\left( P(t)\right) _{t\in \mathbb {R}_{+}}$ defined as

$$\begin{aligned} P(t)(f):=f\circ \psi _{t} \end{aligned}$$

is a generalized Feller semigroup on ${\mathscr {B}}^{\rho }(E)$, if and only if the following conditions hold:

(i) $$\psi _{0}=\text {Id}$$.

(ii) For any $t_{1,}t_{2}\in \mathbb {R}_{+}$

$$\begin{aligned} \psi _{t_{1}}\circ \psi _{t_{2}}=\psi _{t_{1}+t_{2}}. \end{aligned}$$

(iii) For any $x\in E$

$$\begin{aligned} \underset{t\searrow 0}{\lim }\,\psi _{t}(x)=x. \end{aligned}$$

(iv) For any $t\in \mathbb {R}_{+}$ and any $R>0$

$$\begin{aligned} \left. \psi _{t}\right| _{K_{R}}:\,K_{R}\rightarrow E \end{aligned}$$

is continuous.

(v) For any $$t\in \mathbb {R}_{+}$$

$$\begin{aligned} \underset{x\in E}{\sup }\,\frac{\rho \circ \psi _{t}(x)}{\rho (x)}=:C_{t}<\infty . \end{aligned}$$

(vi) For some $\delta >0$ there is $C>0$ such that for all $0\le t<\delta$

$$\begin{aligned} C_{t}<C. \end{aligned}$$

Furthermore, for such a generalized Feller semigroup the identity

6.3 $$\begin{aligned} P(t)\rho (x)=\underset{\begin{array}{c} f\in C_{b}(E)\\ \left| f\right| \le \rho \end{array}}{\sup }\left| f\circ \psi _{t}(x)\right| =\rho \circ \psi _{t}(x) \end{aligned}$$

holds true.

#### Proof

We first show that the conditions (i)-(vi) are sufficient in order to obtain a generalized Feller semigroup.

Fix $t\in \mathbb {R}_{+}$. We show that *P*(*t*) is a bounded linear map from ${\mathscr {B}}^{\rho }(E)$ to ${\mathscr {B}}^{\rho }(E)$. For $f\in {\mathscr {B}}^{\rho }(E)$ and $n\in \mathbb {N}$, by the definition of ${\mathscr {B}}^{\rho }(E)$, there is $f_{n}\in C_{b}(E)$ such that

$$\begin{aligned} \left\| f-f_{n}\right\| _{\rho }<\frac{1}{n}. \end{aligned}$$

By Theorem 2.2, we obtain $f_{n}\circ \psi _{t}\in {\mathscr {B}}^{\rho }(E)$ for any $n\in \mathbb {N}$ since on the one hand $\left. f_{n}\circ \psi _{t}\right| _{K_{R}}\in C_{b}(E)$ holds for any $R>0$ and on the other hand

$$\begin{aligned} \underset{R\rightarrow \infty }{\lim }\underset{x\in E\setminus K_{R}}{\sup }\frac{\left| f_{n}\circ \psi _{t}(x)\right| }{\rho (x)}=0. \end{aligned}$$

The inequality

$$\begin{aligned} \underset{x\in E}{\sup }\,\frac{\left| f\circ \psi _{t}(x)-f_{n}\circ \psi _{t}(x)\right| }{\rho (x)}&=\underset{x\in E}{\sup }\,\frac{\left| f\circ \psi _{t}(x)-f_{n}\circ \psi _{t}(x)\right| }{\rho \circ \psi _{t}(x)}\cdot \frac{\rho \circ \psi _{t}(x)}{\rho (x)}\\&\le \frac{1}{n}\cdot C_{t} \end{aligned}$$

yields that $f\circ \psi _{t}\in {\mathscr {B}}^{\rho }(E)$ as a limit of functions in ${\mathscr {B}}^{\rho }(E).$ Moreover,

$$\begin{aligned} \left\| f\circ \psi _{t}\right\| _{\rho }&=\underset{x\in E}{\sup }\,\frac{\left| f\circ \psi _{t}(x)\right| }{\rho \circ \psi _{t}(x)}\cdot \frac{\rho \circ \psi _{t}(x)}{\rho (x)}\\&\le \left\| f\right\| _{\rho }\cdot C_{t}, \end{aligned}$$

hence *P*(*t*) is a linear bounded operator on ${\mathscr {B}}^{\rho }(E)$. Moreover, the Properties **P1, P2, **and** P5 **of generalized Feller semigroups are easy to check. For Property **P4**, we see that for all $0\le t<\delta$

$$\begin{aligned} \left\| P(t)\right\| \le C_{t}\le C. \end{aligned}$$

Regarding Property **P3,** we observe that for any $x\in E$ and any $0\le t<\delta$ the inequality

$$\begin{aligned} \rho \circ \psi _{t}(x)&\le C_{\delta }\cdot \rho (x)=:R_{x} \end{aligned}$$

holds true. Therefore, $\psi _{t}(x)\in K_{R_{x}}$ for $t\in \left[ 0,\delta \right)$ and because of $\left. f\right| _{K_{R_{x}}}\in C_{b}(E)$ for all $f\in {\mathscr {B}}^{\rho }(E)$ (see Theorem 2.2) we obtain

$$\begin{aligned} \underset{t\searrow 0}{\lim }\,f\circ \psi _{t}(x)=f(x) \end{aligned}$$

for any $$x\in E.$$

Next, we show that if $\left( P(t)\right) _{t\in \mathbb {R}_{+}}$ is a generalized Feller semigroup, then Properties (i)-(vi) and Eq. (6.3) hold true. Property (i) follows from $P(0)=\text {Id}$ which yields

6.4 $$\begin{aligned} f\circ \psi _{0}=f\,\text { for all }f\in {\mathscr {B}}^{\rho }(E). \end{aligned}$$

So by contradiction, if there was some $x\in E$ such that $\psi _{0}(x)\ne x$, then by the definition of completely regular spaces, one could find some map $f_{x}\in C_{b}(E)\subset {\mathscr {B}}^{\rho }(E)$ such that $f_{x}(x)=1$ and $f\circ \psi _{0}(x)=0$. But this would contradict Equation 6.4. Regarding Property (ii), as in the proof of Property (i) we obtain

6.5 $$\begin{aligned} f\circ \left( \psi _{t_{1}}\circ \psi _{t_{2}}\right) =f\circ \left( \psi _{t_{1}+t_{2}}\right) \,\text {for all }f\in {\mathscr {B}}^{\rho }(E) \end{aligned}$$

and as above by contradiction, if Property (ii) did not hold, then one could find a map in ${\mathscr {B}}^{\rho }(E)$ that would contradict Eq. (6.5). Property (iii) can be shown in the same way, since by the definition of generalized Feller semigroups

$$\begin{aligned} \underset{t\searrow 0}{\lim }\,f\circ \psi _{t}(x)=f(x) \end{aligned}$$

holds for any $x\in E$ and any $f\in {\mathscr {B}}^{\rho }(E)$. In order to show Property (iv), we fix some $R>0$ and some arbitrary open set *O* in *E*. We have to show that $\psi _{t}^{-1}(O)$ is open in $K_{R}$ with respect to the subspace topology. We know by Theorem 2.2 that $\left. f\circ \psi _{t}\right| _{K_{R}}$ is continuous for any $f\in {\mathscr {B}}^{\rho }(E)$. For any $x\in O$, by the definition of completely regular spaces, we know that we can find $f_{x}\in C_{b}\left( E\right)$ such that

$$\begin{aligned}  &   \left| f_{x}\right| \le 1, \\  &   f_{x}(x)=1, \end{aligned}$$

and

$$\begin{aligned} f_{x}(E\setminus O)\subset \left\{ 0\right\} . \end{aligned}$$

Clearly,

$$\begin{aligned} \underset{x\in O}{\bigcup }\left( \left. f_{x}\circ \psi _{t}\right| _{K_{R}}\right) ^{-1}\left( 0,2\right) \end{aligned}$$

is open in $K_{R}$ with respect to the subspace topology. On the other hand,

$$\begin{aligned} \underset{x\in O}{\bigcup }\left( f_{x}\right) ^{-1}\left( 0,2\right) =O. \end{aligned}$$

Thus,

$$\begin{aligned} \left. \psi _{t}\right| _{K_{R}}^{-1}(O)&=\left. \psi _{t}\right| _{K_{R}}^{-1}\left( \underset{x\in O}{\bigcup }\left( f_{x}\right) ^{-1}\left( 0,2\right) \right) \\&=\underset{x\in O}{\bigcup }\left( \left. f_{x}\circ \psi _{t}\right| _{K_{R}}\right) ^{-1}\left( 0,2\right) \end{aligned}$$

is open in $K_{R}$ with respect to the subspace topology. Regarding Eq. (6.3), by Remark 2.10$P(t)\rho (x)$ is given for any $x\in E$ by

$$\begin{aligned} P(t)\rho (x)&=\underset{\begin{array}{c} f\in C_{b}(E)\\ \left| f\right| \le \rho \end{array}}{\sup }\left| f\circ \psi _{t}(x)\right| . \end{aligned}$$

We observe that for any $y\in E$ and any $n\in \mathbb {N}$ there is an open neighborhood $O_{n,y}$ of *y* such that

$$\begin{aligned} \rho (x)>\rho (y)-\frac{1}{n} \end{aligned}$$

holds true for any $x\in O_{n,y}$. On $E\setminus O_{n,y}\cup \left\{ y\right\}$, we define the function

$$\begin{aligned} g_{n,y}(x):={\left\{ \begin{array}{ll} \rho (y)-\frac{1}{n} &  \text {for }x=y\\ 0 &  \text {for }x\in E\setminus O_{n,y}, \end{array}\right. } \end{aligned}$$

and by Proposition A.6, we can extend $g_{n,y}$ to $f_{n,y}\in C_{b}(E)$ such that $\left| f_{n,y}\right| <\rho$ and $\rho (y)-f_{n,y}(y)=\frac{1}{n}$. Hence, for any $x\in E$

$$\begin{aligned} \underset{\begin{array}{c} f\in C_{b}(E)\\ \left| f\right| \le \rho \end{array}}{\sup }\left| f\circ \psi _{s}(x)\right| =\rho \circ \psi _{s}(x). \end{aligned}$$

Finally, Property (v) and (vi) follow since for any $x\in E$

$$\begin{aligned} \rho \circ \psi _{t}(x)=P(t)\rho (x), \end{aligned}$$

and by Theorem 2.8, the estimate $\left\| P(t)\right\| \le Me^{\omega t}$ holds true for some $M\ge 1$ and $\omega \in \mathbb {R}$. Thus, Remark 2.10 implies that for any $x\in E$

$$\begin{aligned} \rho \circ \psi _{t}(x)\le \rho (x)Me^{\omega t}. \end{aligned}$$

$$\square $$

Combining the above assertions yields the following corollary.

#### Corollary 6.7

A family $(P(t))_{t \in \mathbb {R}_+}$ is a generalized Feller semigroup of smooth operator algebra homomorphism if and only if there exists a family of maps $\left( \psi _{t}\right) _{t\in \mathbb {R}_{+}}$ from *E* to *E* satisfying the conditions of Proposition 6.6 such that $P(t)(f)=f\circ \psi _{t}$. The family of maps $(\psi _{t})_{t\in \mathbb {R}_{+}}$ is exactly the generalized Feller process constructed in Theorem 3.3.

#### Proof

The first direction was already stated in Proposition 6.5. The other one is a consequence of Proposition 6.6 which yields a generalized Feller semigroup which clearly satisfies the property of being a smooth operator algebra homomorphism. The last assertion just follows from (3.2). $\square$

#### Remark 6.8

The combination of Propositions 6.5 and 6.6 tells that any semiflow satisfying the properties of Proposition 6.6 on a weighted space *E* can be associated with a unique transport operator, i.e., has a version of a tangent direction at any point in time.

Conversely, given a transport operator which generates a generalized Feller semigroup, the associated Feller process constructed in Theorem 3.3 corresponds to $(\psi _t)_{t \in \mathbb {R}_+}$. For conditions when a transport operator generates a generalized Feller semigroup, we refer to Theorem 3.3 in [19], which provides a reformulation of the Lumer–Philips theorem for the quasi-contractive case. Notice, however, that a transport semigroup does not need to be quasi-contractive.

### Polynomial and affine processes

For the definition and theory of polynomial and affine processes, we refer to [13, 23] and [20]. For $n\in \mathbb {N}$, we denote by *E* be a closed subset of $\mathbb {R}^n$. To introduce polynomial processes, we let $\mathcal {P}_{m}$ be the space of polynomials on *E* up to degree $m \in \mathbb {N}$. For (*m*-)polynomial processes with state space *E*, we always consider a version with càdlàg paths and denote by $\left( P(t)\right) _{t\in {\mathbb {R}_+}}$ its Markovian semigroup and by $\left( p(t)\right) _{t\in {\mathbb {R}_+}}$ its semigroup of transition probabilities. Note that we here always consider polynomial processes whose solution to the martingale problem is unique assuring the Markov property. We refer to [27] for examples where this is not the case, i.e., where the law is not determined by the (extended) infinitesimal generator.

We start with the following lemma which is essentially a consequence of the definition of polynomial processes.

#### Lemma 6.9

Let $\left( \lambda _{t}\right) _{t\in \mathbb {R}_{+}}$ be an *m*-polynomial process and let $\rho \in \mathcal {P}_{k}$ for some $k\in \left\{ 0, \ldots ,m\right\}$. Then there is a bounded linear map $A_{k}$ on $\mathcal {P}_{k}$ and $C>0$ such that for all $x\in E$ and $t\in \mathbb {R}_{+}$

$$\begin{aligned} P(t)\rho (x)=\mathbb {E}_{x}\left[ \rho (\lambda _{t})\right] =\left( e^{tA_{m}}\rho \right) (x)\le C e^{t\left\| A_{m}\right\| } \rho (x) \end{aligned}$$

holds true and $\mathbb {E}_{\nu }\left[ \rho (\lambda _{t})\right] <\infty$ for all $t\in \mathbb {R}_{+}$ and for any probability measure $\nu \in \mathcal {M}^{\rho }(E)$.

#### Proof

This follows directly from Theorem 2.7 (ii) in [13]. $\square$

Next we establish the generalized Feller property for polynomial processes under a continuity assumption on the semigroup.

#### Proposition 6.10

For $m\in \mathbb {N}$, let $\left( \lambda _{t}\right) _{t\in \mathbb {R}_{+}}$ be an *m*-polynomial process and let $\rho \in \mathcal {P}_{m}$ be an admissible weight function on *E*. For any $f\in C_{b}(E)$ and any $t\in \mathbb {R}_{+}$ let $\left. P(t)f\right| _{K_{R}}$ be continuous for any $R>0.$ Then $\left( \lambda _{t}\right) _{t\in \mathbb {R}_{+}}$ is a generalized Feller process on $\left( E,\rho \right)$.

#### Proof

We have to show that $\left( \lambda _{t}\right) _{t\in \mathbb {R}_{+}}$ is the stochastic process constructed in Theorem 3.3. By definition of the Markov process $\left( \lambda _{t}\right) _{t\in \mathbb {R}_{+}}$ for any $t\ge s\ge 0$, any probability measure $\nu \in \mathcal {M}^{\rho }(E)$ and any measurable map $f:\,E\rightarrow \mathbb {\mathbb {R}}_{+}$

6.6 $$\begin{aligned} \mathbb {E}_{\mathbb {P}_{\nu }}\left[ \left. f(\lambda _{t})\right| \mathcal {F}_{s}\right] =P\left( t-s\right) f(\lambda _{s}) \end{aligned}$$

holds true $\mathbb {P}_{\nu }$-almost surely and

$$\begin{aligned} \mathbb {\mathbb {P}_{\nu }}\circ \lambda _{0}^{-1}=\nu . \end{aligned}$$

By Lemma 6.9

$$\begin{aligned} \mathbb {E}_{\mathbb {P}_{\nu }}\left[ \left. f(\lambda _{t})\right| \mathcal {F}_{s}\right] =P\left( t-s\right) f(\lambda _{s}) \end{aligned}$$

holds true $\mathbb {P}_{\nu }$ -almost surely for all $f\in \mathscr {B}^{\rho }(E)$ as well. In order to show that $\left( \lambda _{t}\right) _{t\in \mathbb {R}_{+}}$ is a generalized Feller process we still have to prove that $\left( P(t)\right) _{t\in \mathbb {R}_{+}}$ is a generalized Feller semigroup. We fix some $t\in \mathbb {R}_{+}$ and first show that $f\in {\mathscr {B}}^{\rho }(E)$ implies $P\left( t\right) f\in {\mathscr {B}}^{\rho }(E)$. By Lemma 6.9 for any $f \in {\mathscr {B}}^{\rho }(E)$ the map

$$\begin{aligned} x&\rightarrow \int _{E}f(y)p(t)(x,dy) \end{aligned}$$

is well defined and for some $C>0$

$$\begin{aligned} P\left( t\right) f(x)&=\int _{E}f(y)p(t)(x,dy)\\&\le C e^{t\left\| A_{m}\right\| }\left\| f \right\| _{\rho } \rho (x). \end{aligned}$$

In order to show $P\left( t\right) f\in {\mathscr {B}}^{\rho }(E)$ for any $f\in {\mathscr {B}}^{\rho }(E)$, by continuity of $P\left( t\right)$ with respect to $\left\| \cdot \right\| _{\rho }$ and density of $C_{b}(E)$ in ${\mathscr {B}}^{\rho }(E)$ it is sufficient to show that $P\left( t\right) f\in {\mathscr {B}}^{\rho }(E)$ holds true for any $f\in C_{b}(E)$. By Theorem 2.2, this is the case since *P*(*t*)*f* is clearly bounded and by assumption $\left. P(t)f\right| _{K_{R}}$ is continuous for any $R>0$.

Regarding the properties of generalized Feller semigroups in Definition 2.7, **P1**, **P2** and positivity **(P5**) are clearly satisfied for $\left( P(t)\right) _{t\in \mathbb {R}_{+}}$. Property **P4** holds true due to Lemma 6.9. Regarding **P3**, since the paths of $\left( \lambda _{t}\right) _{t\in \mathbb {R}_{+}}$ are càdlàg for all $f\in C_b(E)$ and all $x\in E$ we obtain by dominated convergence

$$\begin{aligned} \underset{t\searrow 0}{\lim }\,P(t)f(x)=\underset{t\searrow 0}{\lim }\,\mathbb {E}_{x}\left[ f(\lambda _{t})\right] =f(x). \end{aligned}$$

Hence, **P3** follows from density of $f\in C_b(E)$ in ${\mathscr {B}}^{\rho }(E)$ and from Lemma 6.9. Thus, $\left( P(t)\right) _{t\in \mathbb {R}_{+}}$ is a generalized Feller semigroup and $\left( \lambda _{t}\right) _{t\in \mathbb {R}_{+}}$ is a generalized Feller process on $\left( E,\rho \right) .$ $\square$

We now turn to affine processes. For their definition on general state spaces $E\subset \mathbb {R}^n$, we refer to [16]. Following Theorem 5.8 in [16], we write the part of the differential semimartingale characteristics of the affine process that corresponds to the compensator of the jump measure as $K(x,d{\xi })$, its linear part according to Theorem 6.4 in [16] as

$$\begin{aligned} \mu (x,d{\xi })=x_{1}\mu _{1}(d{\xi })+\cdots +x_{n}\mu _{n}(d{\xi }), \end{aligned}$$

and its constant part as $m(x,d{\xi })$. As in Example 3.1 in [13], one can show the following lemma.

#### Lemma 6.11

Consider a state space $E\subset \mathbb {R}^{n}$ that contains $n+1$ elements $x_{1}, \ldots ,x_{n+1}$ such that for every $j\in \left\{ 1, \ldots ,n+1\right\}$ the set

$$\begin{aligned} \left( x_{1}-x_{j}, \ldots ,x_{j-1}-x_{j},x_{j+1}-x_{j}, \ldots ,x_{n+1}-x_{j}\right) \end{aligned}$$

is linearly independent. Then an affine process $\left( \lambda _{t}\right) _{t\in \mathbb {R}_{+}}$ is *r*-polynomial with $r\ge 2$ if the killing rate is constant, and

$$\begin{aligned} \int _{\left\| \xi \right\| >1}\left\| \xi \right\| ^{r}m\left( d\xi \right) <\infty , \end{aligned}$$

and for any $i\in \left\{ 1, \ldots ,n\right\}$

$$\begin{aligned} \int _{\left\| \xi \right\| >1}\left\| \xi \right\| ^{r}\mu _{i}\left( d\xi \right) <\infty . \end{aligned}$$

#### Proof

From Theorem 6.4 in [16], it follows that there is $C>0$ such that

$$\begin{aligned} \int _{\mathbb {R}^{d}}\left\| \xi \right\| ^{r}K\left( \lambda _{t},d\xi \right) \le C\left( 1+\left\| \lambda _{t}1_{\left\{ \left. t\in \mathbb {R}_{+}\right| \lambda _{t}\ne \Delta \right\} }\right\| ^{r}\right) . \end{aligned}$$

The lemma follows then from Theorem 2.15 in [13]. $\square$

Under the conditions of the above lemma and a continuity assumption on the semigroup, we now also obtain the generalized Feller property for affine processes.

#### Corollary 6.12

Consider a state space $E\subset \mathbb {R}^{n}$ that satisfies the conditions of Lemma 6.11. Let $r\ge 2$ and assume that the linear part of the killing rate vanishes and that

$$\begin{aligned} \int _{\left\| \xi \right\|>1}\left\| \xi \right\| ^{r}m\left( d\xi \right)<\infty , \qquad \text {and} \qquad \int _{\left\| \xi \right\| >1}\left\| \xi \right\| ^{r}\mu _{i}\left( d\xi \right) <\infty , \quad i\in \left\{ 1, \ldots ,n\right\} . \end{aligned}$$

Let $\rho \in \mathcal {P}_{r}$ be an admissible weight function on *E*. If for any $f\in C_{b}(E)$ and any $t\in \mathbb {R}_{+}$, $\left. P(t)f\right| _{K_{R}}$ is continuous for any $R>0$, then the affine process $\left( \lambda _{t}\right) _{t\in \mathbb {R}_{+}}$ is a generalized Feller process on $\left( E,\rho \right) .$

#### Proof

Combine Proposition 6.10 and Lemma 6.11. $\square$

### Necessity of condition **P4**

For classical Feller semigroups, it is sufficient to require the properties **P1**, **P2**, **P3** and **P5** of a generalized Feller semigroup in order to obtain a strongly continuous semigroup on $C_0(E)$. For generalized Feller semigroups, we need to require additionally **P4**. Indeed, subsequently we construct a semigroup on $\mathscr {B}^{\rho }(E)$ that fulfills **P1**, **P2**, **P3** and **P5** but not **P4** and that is *not* strongly continuous. To this end, we consider $E = \mathbb {N}$ as state space and for $n \ge 0$ take $\rho (n) = \exp (n^2)$. The point 0 is an absorbing state. We construct a Markov chain with discrete state space and continuous time whose transition rate “matrix” $A=(a_{ij})_{i,j \in \mathbb {N}}$ is given by

$$\begin{aligned}&a_{n,n + 1} = n^\alpha \exp (- n), \, a_{n,n} = - n^\alpha , a_{n,0} = n^\alpha (1 - \exp (- n)),&\quad \text {for { n} odd,} \\&a_{n,n} = - (n-1)^{\alpha }, \, a_{n,0} = (n-1)^{\alpha }&\quad \text {for { n} even,} \end{aligned}$$

and 0 otherwise, where $\alpha > 1$. The process jumps from odd numbers with high intensity and low probability up to the next even number and then with high intensity down to 0 which it the absorbing state. From even numbers, it jumps directly down to the absorbing state.

In the following proposition, we show the form of the corresponding Markov semigroup and that it is well defined on $\mathscr {B}^{\rho }(\mathbb {N})$.

#### Proposition 6.13

The Markov semigroup $P(t) = \exp (At)$ is well defined on $\mathscr {B}^{\rho }(\mathbb {N})$. Furthermore, for odd *n* we have for $t \ge 0$

$$\begin{aligned} P(t) \rho (n)&= \rho (n) \exp (- n^\alpha t) \\  &\quad + \rho (n+1) n^{\alpha } t \exp (- n^{\alpha } t) \exp (-n) \\  &\quad + \rho (0) \left( 1 - \exp (- n^\alpha t) -\exp (- n) n^\alpha t \exp (- n^{\alpha } t)\right) . \end{aligned}$$

#### Proof

Solving the Kolmogorov forward equations for the transition probabilities, i.e.,

$$\begin{aligned} \partial _t p_{i,j}(t)= \sum _{k} p_{i,k}(t) a_{k,j} \end{aligned}$$

via the matrix exponential $\exp (At)$ yields for even *n*

$$\begin{aligned} p_{n,n} (t) = \exp (- (n-1)^{\alpha } t), \quad p_{n,0} (t) = 1 - \exp (-(n-1)^{\alpha } t). \end{aligned}$$

and for odd *n*

$$\begin{aligned} p_{n,n} (t)&= \exp (- n^\alpha t), \\ p_{n,n+1} (t)&=\exp (-n) n^\alpha t \exp (-n^{\alpha }t) \\ p_{n,0} (t)&= 1 - \exp (- n^\alpha t) -\exp (-n) n^\alpha t \exp (-n^{\alpha }t). \end{aligned}$$

All other quantities are zero except of $p_{0,0}$ which is equal to 1, as 0 is an absorbing state. This implies existence of the Markov process with transition rate matrix *A*. Furthermore, for every *t* it follows that $P(t) \rho \le C_t \rho$ , whence the semigroup is well defined on $B^{\rho }(\mathbb {N})$. Due to the topology on $\mathbb {N}$, the semigroup is also well defined on $\mathscr {B}^{\rho }(\mathbb {N})$. $\square$

For this semigroup, the validity of **P1**, **P2**, **P3** and **P5** is clear. We show that **P4** does not hold. We define for any $t\in \mathbb {R}_+$

$$\begin{aligned} s(t):=\sup _{n \in \mathbb {N}} \frac{ \rho (n+1) n^{\alpha } t \exp (- n^{\alpha } t) \exp (-n)}{\rho (n)}. \end{aligned}$$

Maximizing this for $n \in \mathbb {R}_+$ using standard methods yields for the maximizing $n_t$ the condition

$$\begin{aligned} n_t^{\alpha }&=\frac{n_t}{\alpha t}+\frac{1}{t}. \end{aligned}$$

Thus, $t\rightarrow 0$ implies $n_t \rightarrow \infty$ and we can estimate for large $n_t$

$$\begin{aligned} s(t)&\ge e^1\left( \lfloor n_t \rfloor ^{\alpha }t\right) \cdot \exp \left( \lfloor n_t \rfloor -\lfloor n_t \rfloor ^{\alpha }t\right) \\&\ge e^1\left( \frac{n_t}{\alpha }+1-(n_t^{\alpha }-\lfloor n_t \rfloor ^{\alpha })t\right) \cdot \exp \left( -2-\frac{n_t}{\alpha } +n_t\right) \\&\ge e^1\left( \frac{n_t}{\alpha }+1-\frac{\alpha (\alpha +1)}{n_t}n_t^{\alpha }t\right) \cdot \exp \left( -2-\frac{n_t}{\alpha } +n_t\right) , \end{aligned}$$

where we used a Taylor expansion in the last step. Using again the expression for $n_t^{\alpha }$, we obtain that $t\rightarrow 0$ implies $s(t)\rightarrow \infty$. Hence, **P4** does not hold. In particular, this calculation shows that for any $f\in \mathscr {B}^{\rho }(\mathbb {N})$ that behaves like $\rho$ around a large enough $n\in \mathbb {N}$ and is 0 elsewhere *P*(*t*)*f* does not converge in norm to *f* for $t \rightarrow 0$. In other words, the semigroup is not strongly continuous. However, when identifying the state 0 with the cemetery $\Delta$, the semigroup has the classical Feller property. Note that Proposition 5.1 does not apply here, precisely because **P4** does not hold true.

## Data Availability

Data sharing is not applicable to this article as no datasets were generated or analyzed during the current study.

## Appendix

We collect in this appendix on the one hand some functional analytic assertions used throughout the paper and on the other hand some lemmas for $\mathscr {B}^{\rho }(E)$ functions.

### Functional analytic tools

#### Definition A.1

Let $\Omega \ne \emptyset$. A set $O\subset \Omega$ is called $C_{b}(\Omega )$-$open$, if there exists a sequence $\left( f_{n}\right) _{n\in \mathbb {N}}\subset C_{b}(\Omega )$ such that pointwise $f_{n}\nearrow 1_{O}$. The system of sets that are $C_{b}(\Omega )$-open is called $\mathcal {G}\left( C_{b}(\Omega )\right)$ .

We recall here the following lemma, which can be found in M. Schweizer’s lecture notes “Measure and Integration” (version July 22, 2017), see Lemma IV.1.11.

#### Lemma A.2

Let $\Omega \ne \emptyset$ and let $\mathcal {G}\left( C_{b}(\Omega )\right)$ be the system of sets that are $C_{b}(\Omega )$-open. Then

$$\begin{aligned} \sigma \left( \mathcal {G}\left( C_{b}(\Omega )\right) \right) =\sigma \left( \left. f\right| \,f\in C_{b}(\Omega )\right) , \end{aligned}$$

is the smallest $\sigma$-algebra such that all maps in $C_{b}(\Omega )$ are measurable.

#### Definition A.3

The smallest $\sigma$-algebra such that all maps in $C(\Omega )$ (or all maps in $C_b(\Omega )$) are measurable is called $Baire \sigma$-$algebra$ and is denoted by $\mathcal {B}_{0}(\Omega )$.

Making slight adjustments in the proof of ([10], §5, Proposition 5), one obtains a version of the proposition that holds on $C_{b}\left( X \right)$:

#### Proposition A.4

Let *X* be a completely regular space and $\ell :\,C_{b}\left( X\right) \rightarrow \mathbb {R}$ be a continuous linear map. There exists a signed Radon measure $\mu$ on *X* such that for all $f\in C_{b}\left( X \right)$

$$\begin{aligned} \ell (f)=\int _{X}f(x)\mu (dx), \end{aligned}$$

if and only if for each $\varepsilon >0$ there exists a compact set $K_{\varepsilon }\subset X$ such that for any function $f\in C_{b}\left( X \right)$ with $\left| f\right| \le 1$ and $\left. f\right| _{K_{\varepsilon }}=0$, we have $\left| \ell (f)\right| <\varepsilon$. The signed Radon measure is unique.

#### Definition A.5

A family $\mathcal {C}$ of subsets of a space *X* is called *compact* *class* if for any sequence $\left( C_{n}\right) _{n\in \mathbb {N}}\subset \mathcal {C}$ such that the intersection $\underset{n\in \mathbb {N}}{\bigcap }C_{n}$ is empty, already some finite intersection $\underset{i\in I,\text { finite}}{\bigcap }C_{i}$ is empty.

For a completely regular space statements, similar to Tietze–Urysohn extension theorem and Urysohn’s lemma can be shown.

#### Proposition A.6

Let *E* be completely regular and $K\subset E$ compact. Then a real-valued continuous function $f\in C\left( K \right)$ on *K* can be extended to a continuous function $F\in C\left( E \right)$ on all of *E*. If additionally $\left| f\right|<C<\infty$ then there is an extension $F\in C\left( E \right)$ such that $\left| F\right|<C<\infty$.

#### Proof

We would like to apply the Tietze–Urysohn extension theorem. However, it allows the extension only on normal spaces. But since a space is completely regular if and only if it is a subspace of a compact space (cf. Proposition 3 in Chapter IX of [9]), we can embed *E* by an embedding *i* in a compact Hausdorff set *N* which is normal. Then *i*(*K*) is also compact on *N* with respect to the subspace topology $\tau (i(E))$ on *N*, hence compact with respect to the topology of *N*. Since *N* is Hausdorff, the compact set *i*(*K*) is closed. We can apply the Tietze–Urysohn extension theorem on *N* to extend the function $f\circ i^{-1}\in C(i(K))$ to a continuous function $G\in C(N)$ such

$$\begin{aligned} \left. f\circ i^{-1}\right| _{i(K)}=\left. G\right| _{i(K)} \end{aligned}$$

and $\left| G\right| \le C$ if $\left| f\right| \le C$. Therefore, $F:=G\circ i$ possesses the desired properties. $\square$

#### Proposition A.7

(Urysohn’s Lemma in the completely regular case) Let *E* be completely regular, $K\subset E$ compact, $A\subset E$ closed and $A\cap K=\emptyset$. Then there is a continuous function $f:\,E\rightarrow \left[ 0,1\right]$ such that $f(K)=\left\{ 0\right\}$, $f(A)=\left\{ 1\right\}$.

#### Proof

As in Proposition A.6, we embed *E* in a compact Hausdorff set *N* by an embedding denoted by *i*. Then, *i*(*K*) is compact, hence closed in the compact Hausdorff space *N*. Since *i*(*A*) is closed in the subspace topology $\tau (i(E))$, there is a closed set $B\subset N$ such that $B\cap i(E)=i(A)$ and clearly $B\cap i(K)=\emptyset .$ Applying Urysohn’s lemma in the normal space *N*, we see that there is a continuous function $g:\,N\rightarrow \left[ 0,1\right]$ with $g(i(K))=\left\{ 0\right\}$ and $g(B)=\left\{ 1\right\}$. Setting $f:=g\circ i$, we conclude. $\square$

#### Corollary A.8

Let *E* be a completely regular space, $\mathcal {B}(E)$ its Borel $\sigma$-algebra, $m\in \mathbb {N}$, $\mu _1,\ldots ,\mu _m$ a family of measures on $\left( E,\mathcal {B}(E)\right)$ and $B\in \mathcal {B}(E)$. If there is a sequence of compact sets $\left( K_{n}\right) _{n\in \mathbb {N}}$ and of open sets $\left( O_{n}\right) _{n\in \mathbb {N}}$ such that $K_{n}\subset B\subset O_{n}$ for any $n\in \mathbb {N}$ and if for $i\in {1,\ldots ,m}$

$$\begin{aligned} \underset{n\rightarrow \infty }{\lim }\mu _i(O_{n}\setminus K_{n})=0, \end{aligned}$$

then there exists a sequence $\left( f_{n}\right) _{n\in \mathbb {N}}$ of nonnegative continuous functions with $f_{n}\le 1_{O_{n}}$ for any $n\in \mathbb {N}$ such that

$$\begin{aligned} \underset{n\rightarrow \infty }{\lim }f_{n}=1_{B} \end{aligned}$$

in $L^{1}\left( E,\mu _i\right)$ for any $i\in {1,\ldots ,m}$. If $\mu _i$, $i\in {1,\ldots ,m}$ is $\sigma$-finite, then this convergence holds true also $\mu _i$-almost surely.

#### Proof

Thanks to Urysohn’s lemma in the complete regular case, there is a sequence $\left( g_{n}\right) _{n\in \mathbb {N}}$ of nonnegative continuous functions with $1_{K_{n}}\le g_{n}\le 1_{O_{n}}$ for any $n\in \mathbb {N}$ such that $g_{n}\rightarrow 1_{B}$ in $L^{1}\left( E,\mu _i\right)$ for $i\in {1,\ldots ,m}$. Thus, if $\mu _i$ is $\sigma$-finite then there exists a subsequence $\left( g_{n_{k}}\right) _{k\in \mathbb {N}}$ such that $g_{n_{k}}\rightarrow 1_{B} \mu _i$-almost surely. $\square$

### Some lemmas for $\mathscr {B}^{\rho }(E)$ functions

The following lemma is needed for the proof of the existence of generalized Feller processes in Theorem 3.3 and is shown by adapting the reasoning of Example 1.13 in [7].

#### Lemma A.9

Let $n\in \mathbb {N}$ and let $\left( E_{i},\rho _{i}\right)$ $i\in \left\{ 1, \ldots ,n\right\}$ be weighted spaces and

$$\begin{aligned} \rho \left( x_{1}, \ldots ,x_{n}\right) :=\rho _{1}\left( x_{1}\right) \cdot \cdot \cdot \rho _{n}\left( x_{n}\right) . \end{aligned}$$

Then the linear map

$$\begin{aligned} \Psi :\,\,\,\,\,{\mathscr {B}}^{\rho _{1}}(E_{1})\otimes \cdots \otimes {\mathscr {B}}^{\rho _{n}}(E_{n})&\rightarrow {\mathscr {B}}^{\rho }(E_{1}\times \cdots \times E_{n})\\ f_{1}\otimes \cdots \otimes f_{n}&\rightarrow f_{1}\cdot \cdot \cdot f_{n} \end{aligned}$$

is injective and its image is a dense linear subspace of ${\mathscr {B}}^{\rho }(E_{1}\times \cdots \times E_{n}).$

#### Proof

By Lemma 2.1, $\left( E_{1}\times \cdots \times E_{n},\rho \right)$ is indeed a weighted space. Furthermore, for $f_{i}\in {\mathscr {B}}^{\rho _{i}}(E_{i})$, $i\in \left\{ 1, \cdots ,n\right\}$ the map

$$\begin{aligned} \left( x_{1}, \ldots ,x_{n}\right) \rightarrow f_{1}(x_{1})\cdot \cdot \cdot f_{n}(x_{n}) \end{aligned}$$

is in ${\mathscr {B}}^{\rho }(E_{1}\times \cdots \times E_{n})$ which follows from continuity of multiplication.

By definition of the tensor product, the linear map $\Psi$ exists. It is injective since for $0\ne u\in {\mathscr {B}}^{\rho _{1}}(E_{1})\otimes \cdots \otimes {\mathscr {B}}^{\rho _{n}}(E_{n})$ there is $m\in \mathbb {N}$ such that we can choose a representation

$$\begin{aligned} u={\sum _{j=1}^{m}}f_{1}^{j}\otimes \cdots \otimes f_{n}^{j}, \end{aligned}$$

with $\left\{ f_{i}^{j}\right\} _{j\in \left\{ 1, \ldots ,m\right\} }\subset {\mathscr {B}}^{\rho _{i}}(E_{i})$ for any $i\in \left\{ 1, \ldots ,n\right\}$ and $\left\{ f_{1}^{j}\right\} _{j\in \left\{ 1, \ldots ,m\right\} }, \ldots ,\left\{ f_{n}^{j}\right\} _{j\in \left\{ 1, \ldots ,m\right\} }$ linearly independent which implies that for any $i\in \big \{ 1, \ldots ,n-1\big \}$ there is $z_{i}\in E_{i}$ such that $f_{i}^{1}(z_{i})\ne 0$, hence by linear independence of $\big \{ f_{n}^{j}\big \} _{j\in \big \{ 1, \ldots ,m\big \} }$

$$\begin{aligned} {\sum _{j=1}^{m}}f_{1}^{j}(z_{1})\cdot \cdot \cdot f_{n-1}^{j}(z_{n-1})f_{n}^{j}\ne 0. \end{aligned}$$

Density of the image of $\Psi$ follows directly from Stone–Weierstrass for ${\mathscr {B}}^{\rho }$-spaces (Proposition 2.6) as the image is an algebra that separates points and contains $1_{E_{1}\times \cdots \times E_{n}}$. $\square$

The following lemma also needed for Theorem 3.3 states that the composition of a ${\mathscr {B}}^{\rho }(E)$-function with a continuous bounded function is a continuous map.

#### Lemma A.10

Let $h\in C_{b}(\mathbb {R})$ and $f\in {\mathscr {B}}^{\rho }(E)$. Then

$$\begin{aligned} {\mathscr {B}}^{\rho }(E) \rightarrow {\mathscr {B}}^{\rho }(E), \quad f \rightarrow h\circ f \end{aligned}$$

is a continuous map.

#### Proof

Since $\left. h\circ f\right| _{K_{R}}$ is continuous for any $R>0$, by Theorem 2.2$h\circ f\in {\mathscr {B}}^{\rho }(E)$ and the map is well defined. Let $g\in {\mathscr {B}}^{\rho }(E)$, $\varepsilon >0$ and choose $R_{\varepsilon }>\frac{2\left\| h\right\| _{\infty }}{\varepsilon }$. Then

$$\begin{aligned} \left\| h\circ f-h\circ g\right\| _{\rho } \le \varepsilon +\frac{1}{\underset{x\in E}{\inf }\rho (x)}\cdot \left\| \left. \left( h\circ f-h\circ g\right) \right| _{K_{R_{\varepsilon }}}\right\| _{\infty }. \end{aligned}$$

Let $\left[ a,b\right]$ be some interval such that $f(K_{R_{\varepsilon }})\subset \left[ a,b\right]$. There is $\delta >0$ such that $\left| x_{1}-x_{2}\right| <\delta$ implies $\left| h(x_{1})-h(x_{2})\right| <\varepsilon$ for any $x_{1},x_{2}\in \left[ a-1,b+1\right]$. Choosing *g* such that $\left\| f-g\right\| _{\rho }<\frac{\delta }{R_{\varepsilon }}$ yields

$$\begin{aligned} \left\| \left. \left( f-g\right) \right| _{K_{R_{\varepsilon }}}\right\| _{\infty }\le \left\| f-g\right\| _{\rho }\cdot R_{\varepsilon }<\delta , \end{aligned}$$

and consequently,

$$\begin{aligned} \left\| \left. \left( h\circ f-h\circ g\right) \right| _{K_{R_{\varepsilon }}}\right\| _{\infty }<\varepsilon . \end{aligned}$$

$$\square $$

For a locally compact space *E*, the space ${\mathscr {B}}^{\rho }(E)$ is already given by the closure of $C_c(E)$, which is subject of Lemma A.11. This is needed to establish a relationship between generalized Feller and Feller processes in Sect. 5.

#### Lemma A.11

Let *E* be locally compact. Then $C_{c}(E)$ is dense in ${\mathscr {B}}^{\rho }(E)$ with respect to $\left\| \cdot \right\| _{\rho }.$

#### Proof

By definition of ${\mathscr {B}}^{\rho }(E)$, we only need to show that $C_{c}(E)$ is dense in $C_{b}(E)$ with respect to $\left\| \cdot \right\| _{\rho }.$ Let $f\in C_{b}(E)$ and $\varepsilon >0$. Choose $R_{\varepsilon }:=\frac{\left\| f\right\| _{\infty }}{\varepsilon }$. By local compactness, each element in $K_{R_{\varepsilon }}$ has a compact neighborhood hence by compactness of $K_{R_{\varepsilon }}$ finitely many such compact neighborhood cover $K_{R_{\varepsilon }}$. The union of these finitely many neighborhoods is a compact neighborhood $U_{K_{R_{\varepsilon }}}\supset K_{R_{\varepsilon }}.$ Let $V_{K_{R_{\varepsilon }}}\subset U_{K_{R_{\varepsilon }}}$ be an open neighborhood of $K_{R_{\varepsilon }}$ and define the map $\tilde{g}_{\varepsilon }\in C_{b}(K_{R_{\varepsilon }}\cup (U_{K_{R_{\varepsilon }}}\setminus V_{K_{R_{\varepsilon }}}))$ as

$$\begin{aligned} \tilde{g}_{\varepsilon }:&={\left\{ \begin{array}{ll} f &  \text {on }K_{R_{\varepsilon }}\\ 0 &  \text {on }U_{K_{R_{\varepsilon }}}\setminus V_{K_{R_{\varepsilon }}}. \end{array}\right. } \end{aligned}$$

By normality of compact sets and the Tietze–Urysohn theorem (see, e.g., [28]), this map can be extended to $g'_{\varepsilon }\in C_{b}(U_{K_{R_{\varepsilon }}})$ such that $\left\| g'_{\varepsilon }\right\| _{\infty }=\left\| f\right\| _{\infty }$. Subsequently the map $g'_{\varepsilon }$ can be extended to $g_{\varepsilon }\in C_{c}(E)$ with $\left\| g_{\varepsilon }\right\| _{\infty }=\left\| f\right\| _{\infty }$ by stetting $g_{\varepsilon }\equiv 0$ on $E\setminus U_{K_{R_{\varepsilon }}}$. Then

$$\begin{aligned} \left\| g_{\varepsilon }-f\right\| _{\rho }&\le \underset{x\in K_{R_{\varepsilon }}}{\sup }\frac{\left| g_{\varepsilon }(x)-f(x)\right| }{\rho (x)}+\underset{x\in E\setminus K_{R_{\varepsilon }}}{\sup }\frac{\left| g_{\varepsilon }(x)-f(x)\right| }{\rho (x)}\\&\le 0+\frac{2\left\| f\right\| _{\infty }}{R_{\varepsilon }}\\&=2\varepsilon , \end{aligned}$$

which proves the lemma since $\varepsilon >0$ was arbitrary. $\square$

## Footnotes

1 We use here some generic $\sigma$-algebra $\mathcal {E}$ which does not necessarily correspond to the Borel $\sigma$-algebra. Later on it will usually be either the Baire (see Definition A.3) or the Borel $\sigma$-algebra.

2 In contrast to the standard literature on classical Feller semigroups, here *E* is neither separable nor metrizable.
